# Oxidation-resistant AgRuIr alloy nanocages for efficient and enduring oxygen evolution in proton exchange membrane electrolysis

**DOI:** 10.1038/s41467-026-71943-6

**Published:** 2026-04-15

**Authors:** Xiaoxiao Wang, Peiping Yu, Moxuan Liu, Lei Wang, Fanfan Shang, Fangpu Zhang, Zhaojun Liu, Yuke Bai, Kai Liu, Liang Zhang, Shengchun Yang, Qing Zhang, Tao Cheng, Chuanbo Gao

**Affiliations:** 1 Sate Key Laboratory of Multiphase Flow in Power Engineering, Frontier Institute of Science and Technology, Xi’an Jiaotong University, Xi’an, China; 2 Institute of Functional Nano & Soft Materials (FUNSOM), Jiangsu Key Laboratory for Carbon-Based Functional Materials and Devices, Soochow University, Suzhou, China; 3 Jiangsu Key Laboratory of Advanced Negative Carbon Technologies, Soochow University, Suzhou, China; 4 MOE Key Laboratory for Nonequilibrium Synthesis and Modulation of Condensed Matter, School of Physics, Xi’an Jiaotong University, Xi’an, China; 5 Center for High-resolution Electron Microscopy (CħEM), School of Physical Science and Technology, ShanghaiTech University, Shanghai, China; 6 Shanghai Key Laboratory of High-resolution Electron Microscopy, ShanghaiTech University, Shanghai, China

**Keywords:** Hydrogen energy, Electrocatalysis, Heterogeneous catalysis, Electrocatalysis

## Abstract

The global transition to a hydrogen economy relies on efficient and durable catalysts for the oxygen evolution reaction within proton exchange membrane water electrolysis. Conventional metal oxide catalysts, particularly RuO_2_, suffer from overoxidation and corrosion under acidic and high-potential conditions, limiting operational lifetime. Here we report a metallic catalyst composed of metastable AgRuIr alloy nanocages that challenge the prevailing view that metallic materials are unsuitable for this reaction. Mechanistically, the filled *d* orbitals of Ag reduce the oxophilicity of the alloy, weakening oxygen adsorption and preventing oxygen incorporation into the metal lattice. As a result, the nanocages exhibit higher activity than Ru/Ir oxides while maintaining a metallic state at high potentials, thereby fundamentally suppressing overoxidation. In a water electrolysis cell, the catalyst delivers 1 A cm^−2^ at a cell voltage of 1.73 V and operates stably for 1500 hours with negligible voltage increase (0.93 μV h^−1^) and minimal metal dissolution (0.5–0.7% kh^−1^). These results redefine the potential of metallic catalysts for oxygen evolution in proton exchange membrane water electrolysis systems toward large-scale hydrogen production.

## Abstract (web summary)

Conventional oxide catalysts for water splitting often undergo overoxidation and dissolution at high potentials. Here, the authors report a metallic AgRuIr catalyst that resists oxidation and dissolution, providing a robust alternative to oxide-based systems for stable water electrolysis.

## Introduction

Proton exchange membrane water electrolysis (PEMWE) is a key technology for converting intermittent renewable energy resources into hydrogen, which is crucial for transitioning from traditional fossil fuels to a clean and sustainable energy system^1–3^. However, the catalysts used for the oxygen evolution reaction (OER) at the anode present a significant obstacle. Currently, ruthenium (Ru) and iridium (Ir)-based oxides are the most extensively explored catalysts for this reaction^4,5^. While Ir-based catalysts offer greater stability than Ru, they face limited reserves and high costs, and tend to overoxidize into high-valent soluble IrO_3_ species during the OER process. In contrast, Ru-based catalysts exhibit higher intrinsic activity but are more prone to overoxidation into high-valent soluble RuO_4_ and RuO_5_^2−^ species, severely limiting the lifespan of PEMWE systems^6–8^. To date, considerable efforts have been made to enhance the stability of Ru/Ir oxides during the OER. For example, conjugating or doping SnO_2_^9,10^, Pt^11^, Nb^12^, or Mn^13^ has been shown to increase the electron density around the Ru/Ir sites in the oxides, thereby suppressing their overoxidation into soluble species. Moreover, it has been discovered that when lattice oxygen in Ru/Ir oxides participates in the OER via the lattice oxygen oxidation mechanism (LOM)^14,15^, oxygen vacancies form within the oxide, which destabilize the crystal structure and accelerate the catalyst corrosion^16–21^. To inhibit the LOM pathway, various elements, including Er^22,23^, Ti^24^, V^25^, Mn^26^, Ir^27^, Ni^28^, Zn^29^, Sn^30^, and Pb^31^, have been doped into RuO_2_, which stabilizes lattice oxygen by weakening the covalency of the Ru–O bond, thereby suppressing its involvement in the OER. Additionally, reducing Ru–Ru distances in oxide catalysts has also been proposed to facilitate direct O–O coupling to form O_2_ without involving lattice oxygen^32–34^. Although these strategies have improved the stability of Ru/Ir oxide catalysts, many studies still report a gradual but continuous voltage increase to maintain a high current density, suggesting persistent catalyst degradation. Therefore, developing highly stable OER catalysts with minimal Ru/Ir loss at industrial current densities remains a central focus of ongoing research.

**Fig. 1:**
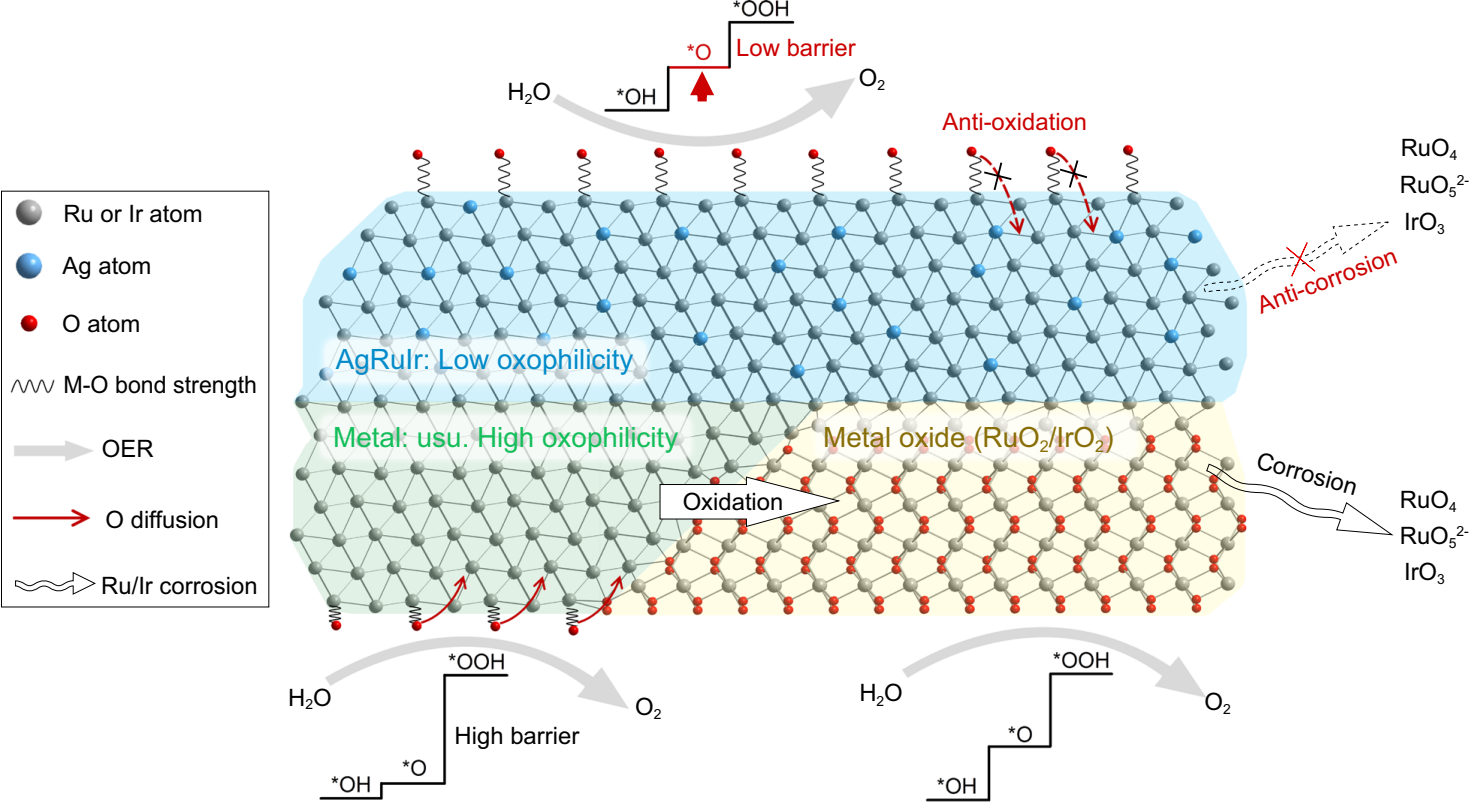
Schematic diagram illustrating the design of an efficient and corrosion-resistant metallic OER catalyst. **Green zone:** Metal nanocrystals with high oxophilicity typically exhibit high energy barriers, resulting in sluggish OER kinetics. **Yellow zone:** Metal nanocrystals are usually converted into metal oxides, such as RuO_2_ and IrO_2_, during the OER. These metal oxides experience continuous overoxidation and corrosion into soluble high-valent oxygenated species, leading to a short lifespan. **Blue zone:** AgRuIr alloy catalyst. Alloying Ag into RuIr reduces the oxophilicity of the metal nanocrystals. This reduced oxophilicity destabilizes *O and lowers the energy barrier for the OER. More importantly, it effectively prevents *O from entering the metal lattice, allowing the AgRuIr nanocrystals to retain their bulk metallic state throughout the OER, reliably preventing overoxidation into soluble high-valent oxygenated species.

To date, most research on acidic OER catalysts has centered on Ru/Ir-based oxides, while metallic catalysts are often excluded as a viable option due to their susceptibility to oxidation under the high potential required for the OER^35–37^. Historically, metals such as Ru and Ir have been explored as OER catalysts for decades; yet, thin surface oxides have been universally observed on these metals during operation (Fig. 1, green to yellow zones)^20,21^. These in situ-formed oxides, amorphous in nature, often corrode even faster than their crystalline rutile counterparts^38,39^. Certain noble metals, including Pt and Au, can maintain a metallic bulk while forming surface oxide monolayers (*OH, *O, and *OOH species) within a specific potential window; however, they exhibit high OER overpotentials (Fig. 1, green zone). Theoretical studies attributed these high overpotentials to intrinsic constraints imposed by the scaling relationship among oxygenated intermediates on these surfaces^40–43^. Collectively, these prior studies contributed to the prevailing notion that metallic catalysts are intrinsically unsuitable for OER. Here, we propose that metallic catalysts remain promising for OER in acidic media. First, the design space can be broadened from monometallic systems to multimetallic alloys, in which electronic hybridization can be leveraged to enhance corrosion resistance. Second, the widely accepted theoretical limitation, i.e., the scaling relationship, was established under the assumption of zero oxygen coverage. In realistic OER conditions, however, metallic surfaces are partially covered by oxygen, depending on their composition. In such cases, the scaling relationship may no longer be valid (as hinted in ref. ^40^), potentially enabling much faster OER kinetics.

In this work, we present an efficient and corrosion-resistant metallic catalyst, AgRuIr alloy nanocrystals, for the acidic OER, which shows enhanced activity and stability relative to established Ru/Ir-based oxide catalysts (Fig. 1, blue zone). Thermodynamically, alloying Ag with RuIr is challenging due to their intrinsic immiscibility^44,45^. We overcome this limitation through a kinetic approach that involves a vacancy-diffusion-assisted alloying mechanism. Specifically, Ag@RuIr core–shell nanocrystals are first synthesized and subsequently subjected to selective Ag etching (Fig. S1). The etching process introduces numerous vacancies, whose extensive diffusion promotes full atomic exchange among Ag, Ru, and Ir, yielding a homogeneous, metastable alloy phase^46,47^. The introduction of Ag, with its fully filled d orbitals, reduces the oxophilicity of the alloy. We find that this modification lowers the OER energy barrier on the metal surface, and more importantly, improves the oxidation resistance of the nanocrystals. As a result, the AgRuIr alloy nanocrystals form only an oxygenated surface layer or an ultrathin oxide layer after extended durations, while maintaining a metallic bulk, even though none of their individual components could withstand the elevated potential alone. The suppression of oxygen incorporation into the metal lattice is confirmed by X-ray photoelectron spectroscopy (XPS) and in situ Raman analyses. This effectively prevents overoxidation into high-valent oxygenated species, resulting in minimal metal loss and enhanced durability compared to conventional oxide-based catalysts. In a PEMWE device, the AgRuIr alloy nanocages deliver a current density of 1 A cm^−2^ at a low potential of 1.73 V and operate stably for 1500 h with negligible voltage degradation (total voltage degradation, ~1 mV; degradation rate, 0.93 μV h^−1^) and minimal metal dissolution (<5.1 ppb in electrolyte; corrosion rate, 0.5 ~ 0.7% kh^−1^). These metrics suggest that the nanocages deliver competitive performance relative to reported OER catalysts.

## Results

### Characterization of metastable AgRuIr alloy nanocages

**Fig. 2:**
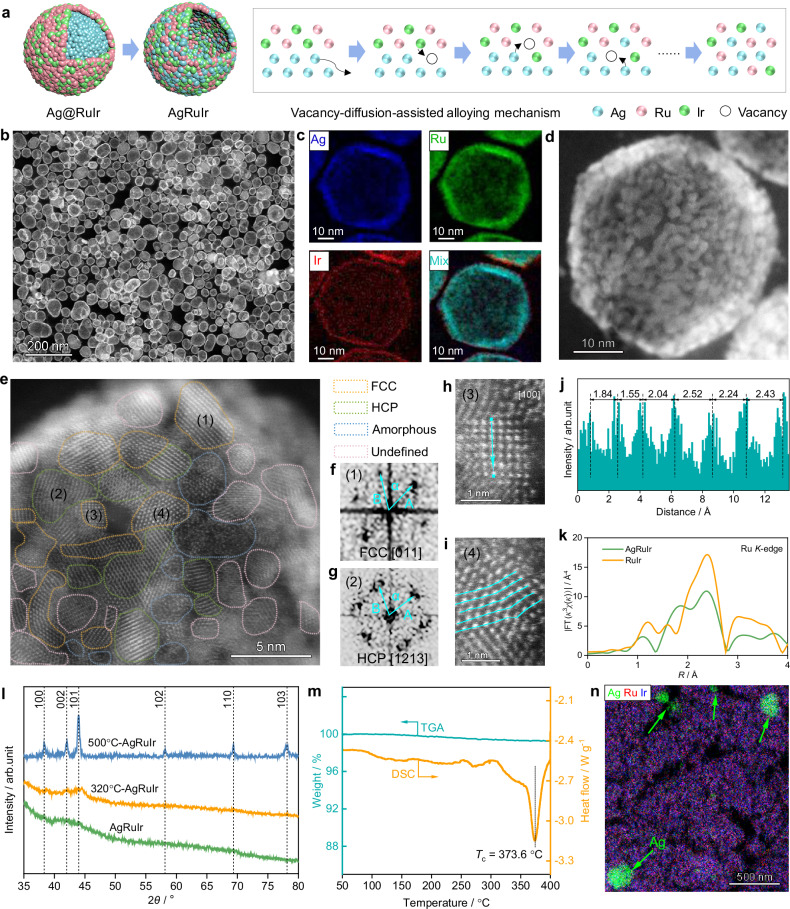
Characterization of the metastable Ag_0.19_Ru_1_Ir_0.48_ alloy nanocages. **a** Schematic illustration of the vacancy-diffusion-assisted alloying mechanism. **b** Low-magnification HAADF-STEM image. **c** EELS mappings of Ag, Ru, and Ir. **d**, **e** *C*_*s*_-corrected high-resolution HAADF-STEM images of a single nanocage. The zones enclosed by yellow, green, blue, and pink dotted lines in (**e**) correspond to FCC, HCP, amorphous, and undefined (structure not resolved) crystallites, respectively. **f**, **g** Fourier diffractograms of crystallites numbered (1) and (2) in (**e**), corresponding to FCC (*B*/*A* = 1.156, *α* = 54.7°) and HCP (*B*/*A* = 1.139, *α* = 64.0°) phases, respectively. **h**, **i** Magnified HAADF-STEM image of the crystallites numbered (3) and (4) in (**e**). **j** Intensity profile along the arrow in (g), showing varying atomic distances along the [001] direction. **k** Ru FT-EXAFS of the AgRuIr alloy nanocages and the RuIr alloy nanoparticles. *R*, radial distance. **l** Temperature-dependent XRD patterns of the AgRuIr alloy nanocages, scan rate, 5° min^−1^. **m** DSC and TGA profiles of the AgRuIr alloy nanocages, scan rate, 10 °C min^−1^. **n** EDS mapping of Ag (green), Ru (red), and Ir (blue) for the nanocages after calcination at 500 °C for 4 h, showing Ag/RuIr phase separation. Source data are provided as a Source Data file.

We synthesized AgRuIr alloy nanocages via a vacancy-diffusion-assisted alloying mechanism (Fig. 2a). In this process, the diffusion of Ag vacancies—generated by HNO_3_ etching—facilitates the full atomic mixing of the constituent metals. This mechanism effectively overcomes the intrinsic immiscibility of the elements, driving the transformation of the initial Ag@RuIr core–shell nanostructures into homogeneous AgRuIr alloy nanocages. In a typical procedure, the Ag@RuIr core-shell nanostructures were first prepared by the epitaxial growth of Ru and Ir on Ag nanospheres in a polyol system. These nanostructures were then subjected to an optimized etching process in 15 wt% HNO_3_ at 25 °C for 30 min, after which the resulting nanocages were collected by centrifugation and washed with deionized water. Control experiments (Fig. S2) demonstrated that under milder conditions (e.g., lower HNO_3_ concentrations), the Ag cores cannot be effectively removed. Conversely, under more aggressive conditions (e.g., higher HNO_3_ concentrations, elevated temperatures, or prolonged etching time), the AgRuIr nanocages became excessively thin, suggesting a continuous loss of Ag from the alloy lattice. Consequently, the etching conditions were optimized to ensure the complete removal of the Ag cores for the formation of the AgRuIr alloy nanocages while preventing extensive dissolution of Ag. This approach allows for a wide composition window, enabling the optimization of catalytic performance.

Figure 2b presents a high-angle annular dark-field scanning transmission electron microscopy (HAADF-STEM) image of the AgRuIr alloy nanocages, revealing the hollow structure resulting from the selective etching. Electron energy loss spectroscopy (EELS) mapping confirmed that the nanocages were composed of Ag, Ru, and Ir, with overlapping distributions, indicating the successful formation of a homogeneous AgRuIr alloy (Fig. 2c). The atomic mixing of the alloy components, facilitated by the vacancy-diffusion-assisted alloying mechanism, maximizes inter-component electronic interactions and ensures homogeneous physicochemical properties throughout the nanocages, thereby enhancing catalytic performance (a similar case in RuIr oxide system, see ref. ^48^). The alloy composition was quantitatively determined to be Ag_0.19_Ru_1_Ir_0.48_ by inductively coupled plasma–mass spectrometry (ICP–MS) (Table S1). Notably, the Ag content in the alloy phase reached 11.4%, demonstrating the effectiveness of our synthesis strategy in overcoming the intrinsic immiscibility of the elements and promoting the formation of a homogeneous, metastable alloy phase. The nanocages consist of numerous crystallites, typically smaller than 2 nm (Fig. 2d, e). Notably, these crystallites exhibit a range of crystalline structures, including face-centered cubic (FCC), hexagonal closed-packed (HCP), and amorphous forms (Fig. 2e), as confirmed by Fourier diffractograms (Fig. 2f, g; additional images, Fig. S3). These crystallites arise from the Volmer–Weber or island growth mode of RuIr on Ag during the synthesis of the Ag@RuIr core–shell nanocrystals, driven by the significant lattice mismatch between RuIr and Ag^49,50^. These island-like nanostructures are retained during the etching-induced alloying process. The small crystallite size increases surface energy, reducing the bulk energy contribution to the overall free energy of the crystallites. This minimizes the impact of bulk energy differences between the alloyed and phase-segregated states of immiscible Ag, Ru, and Ir, thereby stabilizing the metastable alloy phase^51^. This also mitigates the effects of bulk energy differences among FCC, HCP, and amorphous structures, resulting in randomly distributed crystallite forms. Additionally, the HAADF-STEM images reveal disrupted atomic arrangements in the crystallites (Fig. 2h, i) and significant variation in atomic spacings (Fig. 2j). Fourier transform extended X-ray absorption fine structure (FT-EXAFS) shows a broader distribution of Ru and Ir coordination distances in the AgRuIr alloy nanocrystals than in the RuIr alloy nanocrystals, confirming varying atomic arrangements throughout the sample (Figs. 2k, S4). These disrupted atomic arrangements can be attributed to the significant differences in atomic radii among the constituent elements: Ag (1.53 Å), Ru (1.26 Å), and Ir (1.37 Å).

Despite being metastable due to the immiscibility of Ag with both Ru and Ir, the AgRuIr alloy nanocrystals present a sufficiently high energy barrier that prevents their transition to a stable segregated phase. Temperature-dependent X-ray diffraction (XRD) revealed that the AgRuIr alloy nanocages, initially exhibiting low crystallinity, underwent a phase transformation into an HCP phase at approximately 320 °C (Fig. 2l). Differential scanning calorimetry (DSC) analysis confirmed this transition with an exothermic peak at 373.6 °C, while thermogravimetric analysis (TGA) showed no indication of organic species oxidation, indicating that the phase transition occurred at this temperature (Fig. 2m). EDS mapping confirmed the segregation of the AgRuIr alloy into separate Ag and RuIr regions after thermal treatment (Figs. 2n, S5 and S6). These results demonstrate that the AgRuIr alloy is an intrinsically metastable phase with a high phase change barrier, ensuring its retention in practical applications.

### Electrochemical OER activity

**Fig. 3:**
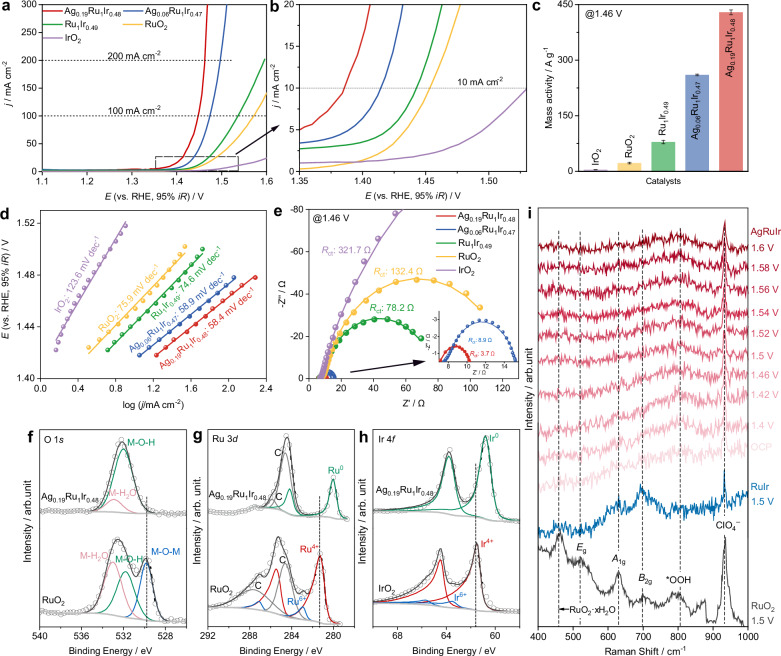
Electrochemical OER activity of the metastable Ag_0.19_Ru_1_Ir_0.48_ alloy nanocages. Catalysts for comparison: Ag_0.06_Ru_1_Ir_0.47_ alloy nanocages, Ru_1_Ir_0.49_ alloy nanoparticles, and commercial RuO_2_ and IrO_2_. **a** LSV curves in O_2_-saturated 0.1 M HClO_4_ (pH = 1.0 ± 0.01) at 25 °C, scan rate: 10 mV s^−1^. *iR* compensation level: 95%. Non-*iR* compensated curves, Fig. S52. Catalyst loading: 0.134 mg cm^−2^, except for 0.3 mg cm^−2^ for IrO_2_. Electrode area: 0.36 cm^2^. Solution resistance: 5.0 ± 0.01 Ω. **b** LSV curves at low current densities, as labeled by the rectangle zone in (**a**). **c** Comparison of the mass activities of the catalysts at 1.46 V. Error bars indicate standard deviations from three parallel measurements. **d** Tafel plots, including Tafel slopes for each catalyst. Spherical symbols represent experimental Tafel data points, and solid lines correspond to the fitted Tafel curves. **e** Nyquist EIS plots, spherical symbols, experimental EIS data points, lines and the fitted EIS curves. Inset: Magnified plots in the low *Z*’ and *Z*” zone. **f**–**h** O 1 *s*, Ru 3 *d*, and Ir 4 *f* XPS spectra of Ag_0.19_Ru_1_Ir_0.48_ compared with those of RuO_2_ and IrO_2_, acquired after chronoamperometric testing at 1.74 V for 2 h. **i** In situ Raman spectra of the Ag_0.06_Ru_1_Ir_0.47_ alloy nanocages at different potentials (OCP–1.6 V) of OER in 0.1 M HClO_4_, compared with the spectra of RuIr alloy and RuO_2_ at 1.5 V. The Raman peak of ClO_4_^−^ is from the electrolyte. The in situ Raman spectroelectrochemical cell was custom-made by GaossUnion (Model: 031; setup, Fig. S53). Source data are provided as a Source Data file.

The metastable Ag_0.19_Ru_1_Ir_0.48_ alloy nanocages demonstrate enhanced catalytic activity for the acidic OER (Fig. 3a–e), showing competitive performance against Ag-deficient Ag_0.06_Ru_1_Ir_0.47_ alloy nanocages (Fig. S7), Ru_1_Ir_0.49_ alloy nanocrystals (Fig. S8), and well-established commercial RuO_2_ and IrO_2_ benchmarks (Fig. S8). Optimization of the shell thickness (≈3.8 nm) and the Ir/Ru ratio (≈0.5) is critical to ensuring the optimal OER performance. Specifically, a sufficient shell thickness is required to maintain a robust nanocage architecture throughout the catalytic process. A control sample with a thinner shell (≈1.6 nm) exhibited rapidly degrading catalytic activity within 30 h during chronopotentiometric testing at 50 mA cm^−2^ (Figs. S9, S10). Additionally, the Ir/Ru ratio within the AgRuIr nanocages significantly influences their OER activity. Control experiments revealed a volcano-shaped dependence of OER activity on the Ir/Ru ratio, with maximum activity achieved at Ir/Ru ≈0.5 (Figs. S11–S13). This trend can be attributed to the electronic interactions between Ru and Ir, which effectively modulate the adsorption energies of oxygenated intermediates and optimize the overall OER kinetics. Consequently, Ag_0.19_Ru_1_Ir_0.48_ nanocages with an average shell thickness of 3.8 nm were selected to demonstrate the competitive OER performance of this AgRuIr alloy catalyst system.

Figure 3a presents linear sweep voltammetry (LSV) curves of the catalysts in O_2_-saturated 0.1 M HClO_4_. Among all the catalysts investigated, the Ag_0.19_Ru_1_Ir_0.48_ alloy nanocages exhibited the highest OER activity. The overpotentials for Ag_0.19_Ru_1_Ir_0.48_ to achieve current densities of 10, 100, and 200 mA cm^−2^ were 160, 215, and 233 mV, respectively, which were lower than those of Ag_0.06_Ru_1_Ir_0.47_, Ru_1_Ir_0.49_, RuO_2_, IrO_2_, and state-of-the-art catalysts (Fig. 3b; detailed comparison, Table S2, S3)^22,27,28,52–54^. The mass activities at 1.46 V vs. the reversible hydrogen electrode (RHE) increased in the following order: IrO_2_ (4.63 ± 0.05 A g^−1^) < RuO_2_ (22.4 ± 2.1 A g^−1^) < Ru_1_Ir_0.49_ (79.2 ± 4.6 A g^−1^) < Ag_0.06_Ru_1_Ir_0.47_ (260.2 ± 2.2 A g^−1^) < Ag_0.19_Ru_1_Ir_0.48_ (429.1 ± 6.5 A g^−1^) (Fig. 3c; values expressed as “mean ± standard deviation” calculated from three parallel measurements). The Ag_0.19_Ru_1_Ir_0.48_ catalyst exhibited the lowest Tafel slope (58.4 mV dec^−1^) (Fig. 3d) and the lowest charge transfer resistance (*R*_ct_), as derived from electrochemical impedance spectroscopy (EIS) (Fig. 3e, Table S4), reflecting enhanced OER kinetics among the investigated catalysts. The higher activity of Ag_0.19_Ru_1_Ir_0.48_ than that of RuO_2_ and IrO_2_ validates the potential of metal nanocrystals as promising OER catalysts, challenging traditional views. Compared with the Ag-deficient Ag_0.06_Ru_1_Ir_0.47_ and Ag-free Ru_1_Ir_0.49_ catalysts, its improved performance highlights the critical role of Ag in enhancing the OER activity of the RuIr-based metal catalysts.

The active species in the metastable AgRuIr alloy nanocages were investigated via X-ray photoelectron spectroscopy (XPS) (Fig. 3f–h). Before analysis, the Ag_0.19_Ru_1_Ir_0.48_ alloy nanocages underwent chronoamperometric operation at 1.74 V for 2 h to ensure that the XPS data reflected the true active species present during the OER. The O 1 *s* XPS can be fitted by two peaks at 532.0 and 532.9 eV, corresponding to surface hydroxyl groups (M–OH) and physisorbed H_2_O (M–H_2_O), respectively (Fig. 3f). Notably, there is no detectable signal at 529.8 eV for lattice oxygen (M–O–M), which contrasts with the O 1 *s* spectrum of a typical metal oxide, RuO_2_^55–58^. Additionally, the Ru 3 *d* and Ir 4 *f* spectra show peaks for Ru^0^ (3*d*_5/2_ at 280.1 eV) and Ir^0^ (4*f*_7/2_ at 60.8 eV), with no signals for their oxides (Fig. 3g, h). The Auger electron spectrum confirms the presence of Ag^0^ rather than Ag oxide (Fig. S14). Collectively, these results demonstrate that the metastable AgRuIr alloy nanocages remain in their metallic state, rather than oxidizing into metal oxides. The active species for the OER can therefore be identified as metallic AgRuIr, or more precisely, metallic AgRuIr with an oxygenated surface layer at the operational potentials.

We further employed in situ Raman spectroscopy to identify the active species in the metastable AgRuIr alloy nanocages during the OER (Fig. 3i). For comparison, RuO_2_ and RuIr alloy nanoparticles were also investigated. The catalysts were deposited onto a glassy carbon slice, which was then placed onto a transparent plastic window inside a flow cell filled with 0.1 M HClO_4_. The flow cell was connected to an electrochemical workstation using a three-electrode configuration. The catalysts were held at different potentials for 5 min before Raman analysis. At the open-circuit potential (OCP), RuO_2_ exhibited three prominent Raman signals at 518, 642, and 702 cm^−1^, corresponding to the *E*_g_, *A*_1g_, and *B*_2g_ vibration modes of the rutile phase, respectively, and an additional peak at 805 cm^−1^, attributed to the OER intermediate *OOH (Fig. S15). At 1.5 V, an additional peak at 460 cm^−1^ appeared, indicating the formation of amorphous hydrated RuO_2_ species (RuO_2_· x H_2_O) on the surface^59,60^. In contrast, the Ag_0.19_Ru_1_Ir_0.48_ alloy nanocages only displayed the Raman signal of *OOH within the potential range from OCP to 1.6 V, with no oxide-related peaks (i.e., surface hydrated or rutile RuO_2_) detected. The RuIr alloy nanoparticles at 1.5 V exhibited oxide-related peaks, including *A*_1g_ and B_2g_ of a rutile-type oxide, as well as amorphous RuO_2_· x H_2_O, suggesting the oxidation of Ru to RuO_2_ at the elevated potential of the OER. These findings confirm that the Ag_0.19_Ru_1_Ir_0.48_ alloy retains its metallic state at elevated potentials for the OER, with Ag playing a pivotal role in enhancing the oxidation resistance of the alloy nanocrystals. The in situ Raman spectroscopy analysis further supports the conclusion that a metal oxide phase did not form to serve as active sites for the OER. Instead, the real active sites are metallic AgRuIr with an oxygenated surface layer, which aligns with the XPS observations.

### Oxidation and corrosion resistance in OER

**Fig. 4:**
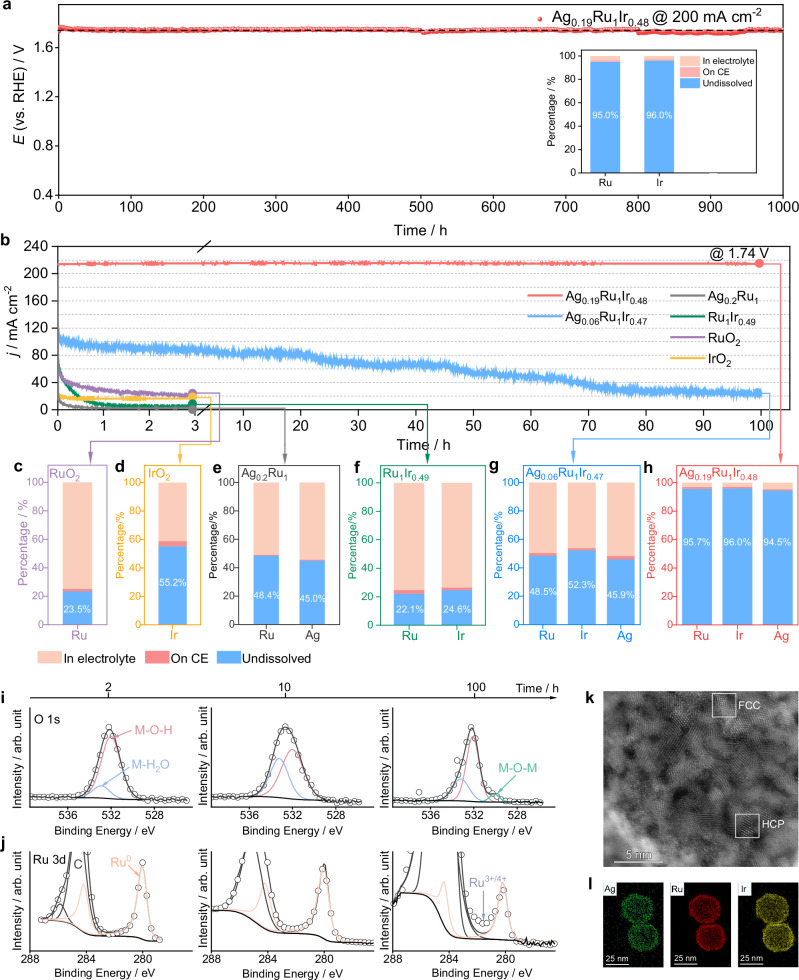
Oxidation and corrosion resistance of the metastable Ag_0.19_Ru_1_Ir_0.48_ alloy nanocages during the OER. **a** Chronopotentiometric curve of the Ag_0.19_Ru_1_Ir_0.48_ catalyst at 200 mA cm^−2^ in 0.1 M HClO_4_ (pH = 1.0 ± 0.01) at 25 °C. Catalyst loading, 0.934 mg cm^−2^. Non-*iR* correction is applied. Inset: Percentages of dissolved Ru and Ir after 1000 h of chronopotentiometric testing. **b** Chronoamperometric curves of the Ag_0.19_Ru_1_Ir_0.48_ catalyst at 1.74 V in 0.1 M HClO_4_ (pH = 1.0 ± 0.01) at 25 °C, compared with those of Ag_0.06_Ru_1_Ir_0.47,_ Ru_1_Ir_0.49_, RuO_2_, and IrO_2_. Catalyst loading, 0.536 mg cm^−2^. Non-*iR* correction is applied. **c–h** Percentages of dissolved metals from different catalysts after the chronoamperometric testing. Test durations: Ag_0.19_Ru_1_Ir_0.48_ and Ag_0.06_Ru_1_Ir_0.47_, 100 h; Ru_1_Ir_0.49_, RuO_2_, and IrO_2_: 3 h. The yellow, pink, and blue bars in (**a**, **c**–**h**) represent the percentages of metals dissolved in the electrolyte, redeposited on the counter electrode (CE), and undissolved metals, respectively. **i**, **j** O 1 *s* and Ru 3 *d* XPS of the Ag_0.19_Ru_1_Ir_0.48_ catalyst after chronoamperometric testing at 1.74 V for 2, 10, and 100 h. **k**, **l** *C*_s_-corrected high-resolution HAADF-STEM image and EDS elemental mappings of the Ag_0.19_Ru_1_Ir_0.48_ catalyst after chronopotentiometric testing at 200 mA cm^−2^ for 200 h. Source data are provided as a Source Data file.

We investigated the catalytic stability of the metastable Ag_0.19_Ru_1_Ir_0.48_ alloy nanocages through chronopotentiometric testing at a high current density of 200 mA cm^−2^ (catalyst loading, 0.934 mg cm^−2^) (Fig. 4a). Remarkably, the catalyst maintained a stable potential over 1000 h of investigation, showing no detectable potential increase (initial: 1.748 V; at 1000 h: 1.742 V, even lower than the initial potential). In stark contrast, RuO_2_, IrO_2_, Ru_1_Ir_0.49_, and Ag_0.06_Ru_1_Ir_0.47_ all exhibited rapid potential increases within 50 h, even at a lower current density of 10 mA cm^−2^ (Fig. S16). This sustained stability, with negligible decay over 1000 h at 200 mA cm^−2^, was achieved under conditions of low catalyst loading and high potential, which typically correlates with increased corrosion rates of OER catalysts^61,62^. This positions Ag_0.19_Ru_1_Ir_0.48_ as a competitive catalyst among reported state-of-the-art catalysts (Fig. 4a left inset and Table S2)^10,27,28,63–65^.

To quantify metal loss during long-term operation, we employed ICP-MS to measure both high-valent soluble metal species in the electrolyte and metallic deposits on the counter electrode (CE) in a half-cell testing (Fig. S17). Undissolved metals, including those retained on the working electrode (WE) and those detached as pristine nanocages (Fig. S18), were determined by mass balance. Over 1000 h of chronopotentiometric testing at 200 mA cm^−2^, the Ag_0.19_Ru_1_Ir_0.48_ alloy nanocages exhibited minimal Ru and Ir losses of 5.0% and 4.0%, respectively (Fig. 4a, right inset, and Table S5; the metal loss includes dissolved metal species in the electrolyte and those redeposited on the counter electrode). Additionally, post-catalytic characterization showed negligible changes in average particle size, consistent with the minimal metal losses detected via ICP-MS (Figs. S19, S20). In contrast, RuO_2_, IrO_2_, and Ag-free Ru_1_Ir_0.49_ suffered significant metal losses of 72.9%, 49.1%, and 74.6% (Ru)/66.1% (Ir), respectively, within less than 30 min (Figs. S21, S22, and Table S5). It is worth noting that the catalyst dissolution observed in half-cell measurements using acidic electrolytes (e.g., 0.1 M HClO_4_) can overestimate the actual metal dissolution rate in PEMWE by more than two orders of magnitude (confirmed experimentally later)^66,67^. The current half-cell data already demonstrate excellent corrosion resistance of the AgRuIr alloy nanocages in acidic OER conditions.

Since metal corrosion rates are correlated with the applied potential, we further evaluated the stability of the Ag_0.19_Ru_1_Ir_0.48_ alloy nanocages through chronoamperometric testing at 1.74 V (Figs. 4b–h, S23 and Table S6). While many reported OER catalysts were evaluated at lower potentials (typically <1.5 V)^27,32,55,68,69^, our testing at the elevated potential provides a more rigorous assessment of the intrinsic corrosion resistance of the catalysts. The Ag_0.19_Ru_1_Ir_0.48_ catalyst exhibited a nearly constant current density (~136 mA cm^−2^) for over 100 h with minimal metal loss detected by ICP-MS (Ag: 5.5%; Ru: 4.3%; Ir: 4.0%) (Fig. 4b, h). Notably, control experiments confirmed that the carbon cloth substrate provided a negligible contribution to the observed OER current densities (Figs. S24, S25). In contrast, RuO_2_ and IrO_2_ rapidly deactivated within 3 h, accompanied by significant metal loss (Ru: 76.5%; Ir: 45.8%), highlighting the enhanced corrosion resistance of the AgRuIr alloy catalyst relative to oxide catalysts (Fig. 4b–d). Additionally, the Ag-deficient Ag_0.06_Ru_1_Ir_0.47_ catalyst showed a continuous current density decline from 112 to 24 mA cm^−2^ and significant metal loss (Ag: 55.1%; Ru: 51.5%; Ir: 47.7%) over 100 h (Fig. 4b, g). The Ag-free Ru_1_Ir_0.49_ catalyst exhibited even more rapid degradation and significant metal loss (Ru: 78.9%; Ir: 75.4%) within 3 h (Fig. 4b, f). These findings indicate that the corrosion behavior and catalytic stability of the AgRuIr alloy catalyst are highly sensitive to the Ag content. Effective corrosion resistance is only realized when a sufficiently high fraction of Ag is incorporated into the alloy lattice. Collectively, these results highlight the critical role of Ag in mitigating oxidative dissolution and enhancing the overall durability of RuIr-based metallic catalysts under acidic OER conditions.

The high corrosion resistance of the Ag_0.19_Ru_1_Ir_0.48_ catalyst is directly linked to its anti-oxidation properties (Fig. 4i–l, Tables S13 and S14). O 1 *s* XPS analysis revealed the presence of surface M–OH and M–H_2_O and the absence of lattice oxygen (M–O–M) in the Ag_0.19_Ru_1_Ir_0.48_ catalyst after chronoamperometric testing at 1.74 V for 2 and 10 h (Fig. 4i). Even after extended operation for 100 h, surface M–OH and M–H_2_O remained the dominant oxygen species (Table S7). Spectroscopic evaluations via Ru 3 *d*, Ir 4 *f* XPS, and Ag Auger electron spectroscopy indicated that Ru and Ag primarily retain their metallic state during the OER, with only IrO_2_ formation after prolonged catalysis (Figs. 4j, S26, S27, Tables S8–S10). Notably, the metallic Ru^0^ state was preserved even after 1000 h of chronopotentiometric testing at 200 mA cm^−2^ (Fig. S28). Bulk-sensitive characterizations confirm the persistence of the metallic framework. Raman spectra of the Ag_0.19_Ru_1_Ir_0.48_ catalyst after 100 h of chronopotentiometric testing at 200 mA cm^−2^ exhibited no oxide-related peaks (i.e., hydrated or rutile RuO_2_), confirming high oxidation resistance at the high OER potentials (Fig. S29). Post-catalytic HAADF-STEM and EDS mapping corroborated the preservation of the metallic crystalline structure and uniform elemental distribution (Figs. 4k, l, S30 and S31). Furthermore, post-catalytic Ru FT-EXAFS provided definitive bulk-sensitive evidence for the retention of the metallic alloy state during the OER process (Fig. S32). In contrast, both Ag-deficient (Ag_0.06_Ru_1_Ir_0.47_) and Ag-free (Ru_1_Ir_0.49_) catalysts exhibited lattice oxygen signals in the O 1 *s* XPS within only 2 h of chronoamperometric testing at a lower potential of 1.6 V, with the lattice oxygen content increasing over time, suggesting continuous metal oxidation (Figs. S33–S35, Table S11–S13). This comparison highlights the critical role of Ag in enhancing the oxidation resistance of the AgRuIr alloy. Crucially, the persistence of metallic Ru alongside the in situ formation of IrO_2_ after extended catalysis suggests a synergistic mechanism between the two phases. The electronic interaction at the interface of the stable metallic Ru and the protective IrO_2_ layer likely provides the necessary environment to sustain high OER activity while effectively shielding the Ru from excessive oxidation and subsequent dissolution.

### Role of Ag in enhancing OER activity and oxidation resistance

The role of Ag can be understood through the concept of oxophilicity (*ϴ*)^70^. Both Ru and Ir exhibit high oxophilicity (*ϴ* ≈ 0.4), making them prone to oxidation at elevated potentials. We hypothesize that alloying RuIr with a metal that has low oxophilicity could not only modulate the adsorption energies of oxygenated intermediates involved in the OER, optimizing the reaction energy barrier, but also decrease the tendency for oxygen to diffuse into the metal lattice, thereby suppressing the formation of metal oxides and subsequent soluble high-valent oxygenated species during the OER. To validate this hypothesis, we assessed the OER activities and corrosion resistance of various metastable MRuIr (M = Ag, Cu, Ni, Co) alloy nanocages (Figs. S36–S42, Tables S14, S15). Among these metals, Ag and Cu exhibit lower oxophilicity (*ϴ* ≈ 0.2) and fully filled d orbitals, effectively enhancing the OER activity and corrosion resistance. In contrast, Ni, which has low oxophilicity (*ϴ* ≈ 0.2) but partially filled d orbitals, and Co, with high oxophilicity (*ϴ* ≈ 0.4), showed less promising results. The catalytic activity and corrosion resistance of the MRuIr alloy catalysts correlate closely with the oxophilicity of M, following the order Co < Ni < Cu < Ag, thus validating our hypothesis. It is noteworthy that Ir is also essential for achieving corrosion resistance in the AgRuIr alloy catalyst, as the Ir-free Ag_0.2_Ru_1_ counterpart rapidly degrades during the OER (Fig. 4c, f). We propose that Ir elevates the reduction potentials of the alloy components through electronic hybridization (standard reduction potentials for pure Ru, Ag, and Ir are 0.455, 0.800, and 1.156 V, respectively), thus synergistically enhancing the oxidation resistance of the alloy nanocages. To validate this mechanism, we utilized in situ Raman spectroscopy to monitor the potential-dependent surface oxidation of a binary RuIr alloy system compared to pure Ru (Fig. S43). For Ru/C, characteristic vibrational modes corresponding to rutile-type oxide species and amorphous RuO_2_·H_2_O began to emerge at potentials as low as ~1.0 V vs. RHE. In contrast, the RuIr/C catalyst remained relatively featureless until significantly higher potentials were reached (~1.5 V vs. RHE). This clear delay in the formation of surface oxides provides direct experimental evidence that Ir elevates the reduction potentials of the alloy surface, maintaining thermodynamic stability under demanding OER conditions.

Beyond oxophilicity, we considered the influence of geometric factors like lattice strain and structural disorder. While Ag introduces tensile strain, our comparative studies with CuRuIr, which impose compressive strain due to the smaller radii of Cu, also showed enhanced OER activity compared with RuIr (Fig. S41). This suggests that the specific nature of lattice strain is not the primary driver of performance. Furthermore, while the structural disorder inherent in these metastable alloys likely provides a beneficial distribution of active sites, only the AgRuIr system demonstrated high durability (Fig. S42). These results indicate that while lattice distortion may contribute to the enhanced activity, the modulation of surface oxophilicity remains the decisive mechanism for long-term stability.

**Fig. 5:**
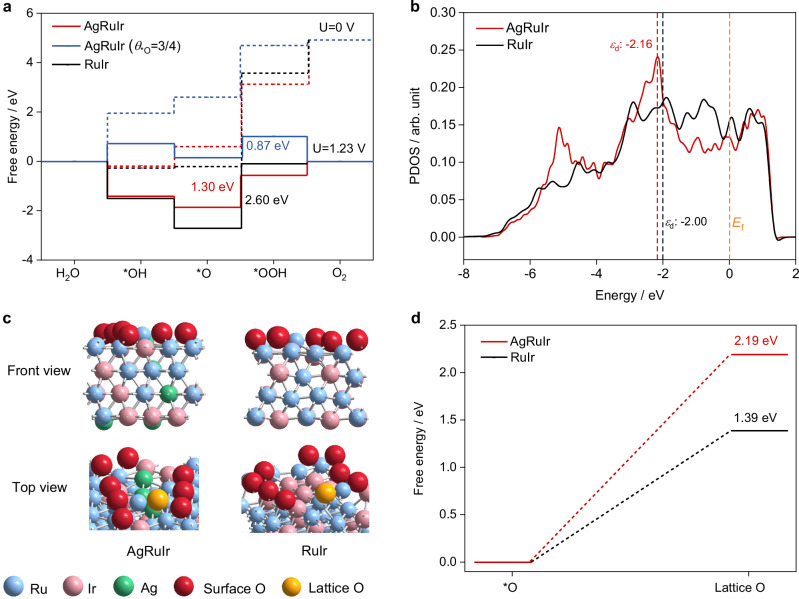
DFT calculations. **a** Gibbs free energy profile of the OER on AgRuIr and RuIr surfaces. **b** PDOS analysis of the Ru and Ir active sites on the AgRuIr and RuIr surfaces. *E*_f_, Fermi level. *ε*_d_, d-band center. **c** Atomic model diagrams of AgRuIr and RuIr with surface and lattice oxygen atoms. **d** Calculated formation energy for lattice oxygen in the AgRuIr and RuIr lattices. Source data are provided as a Source Data file.

We further conducted density functional theory (DFT) calculations to evaluate the role of Ag in the AgRuIr alloy nanocages (Fig. 5). Two models were constructed via enumerating quasi-random structures: Ag_8_Ru_38_Ir_18_ representing the Ag_0.19_Ru_1_Ir_0.48_ alloy nanocages, and Ru_43_Ir_21_ depicting the Ru_1_Ir_0.49_ alloy nanoparticles (Fig. S44). The optimized structures of AgRuIr and RuIr models are provided in Supplementary Data 1. It is important to note that deviations of these models from real catalytic surfaces are inevitable due to the dynamic nature of catalysts under complex OER conditions. Therefore, these calculations are intended to provide qualitative trends rather than exact quantitative predictions of the catalytic process.

The widely accepted adsorbate evolution mechanism (AEM) was considered to analyze the elementary reactions (Figs. 5a, S45)^71^. On the RuIr surface, the *O → *OOH step was identified as the potential-determining step (PDS) with an energy increase of 3.83 eV at *U* = 0 and 2.60 eV at *U* = 1.23 V, which is consistent with the sluggish OER kinetics typically observed for metallic catalysts. On the AgRuIr surface, the energy increase for the PDS of the *O → *OOH step decreased significantly to 2.53 eV at *U* = 0 and 1.30 eV at *U* = 1.23 V. This reduction in the energy increase might be attributed to the destabilization of divalent *O relative to monovalent *OOH on the AgRuIr surface. Projected density of states (PDOS) analysis suggested that incorporating Ag into the RuIr lattice caused a shift in the d band center (*ε*_d_) away from the Fermi level (*E*_f_), weakening the *O adsorption energy (Fig. 5b). These results are consistent with our hypothesis that the low oxophilicity of Ag modulates the adsorption energies of OER intermediates, accelerating the reaction kinetics. Additionally, the surface oxygen coverage (θ_*O_) was also found to be an important factor influencing OER kinetics, aligning with the theoretical framework established by Nørskov and co-workers^40^. Given the high energy increase required for *O → *OOH, *O species tend to accumulate on the metal surface, reaching high coverage before the OER proceeds. Our DFT calculations suggest that with increasing θ_*O_, the energy increase for the PDS (*O → *OOH) decreases (Fig. S46). Specifically, at θ_*O_ = 3/4, the energy increase for this step reaches 2.10 eV at *U* = 0 and 0.87 eV at *U* = 1.23 V. This reduction in oxygen coverage may stem from the differential modulation of adsorption energies: increasing surface oxygen coverage destabilizes *O more significantly than *OOH. This relative destabilization reduces the energy increase required for *OOH formation, thereby promoting the overall OER kinetics. These results suggest that AgRuIr, characterized by a spontaneously formed surface *O layer at operating potentials, is highly efficient for OER catalysis.

According to the universal scaling relationship, metallic nanocrystals are generally considered less effective OER catalysts than metal oxides, when the free energy difference between *OH and *OOH (ΔG_*OH/*OOH_) is constant^40,43^. However, our calculations suggest that ΔG_*OH/*OOH_ decreases in the order RuIr (3.85 eV) > AgRuIr (3.31 eV) > AgRuIr (θ_*O_ = 3/4) (2.85 eV), indicating a deviation from the expected scaling relationship (Fig. 5a). This deviation can be attributed to two key factors. First, alloying could have created heterogeneous surface binding sites, allowing intermediates to bind at distinct sites for their conversion^72,73^. Second, the presence of surface *O may exert a nonlinear influence on the adsorption energies of different oxygenated intermediates^40^. Both effects may have disrupted the scaling relationship, leading to enhanced OER activities.

Previous studies have shown that the corrosion of metallic Ru/Ir catalysts occurs primarily via overoxidation into high-valent oxygenated species rather than direct demetallation into cations^20,39,74^. Based on this understanding, we explored the role of Ag in enhancing the corrosion resistance of the AgRuIr alloy nanocrystals by investigating the formation energy of lattice oxygen (Fig. 5c, d). Two models, i.e., AgRuIr and RuIr alloys with surface *O adsorption, were considered in the DFT calculations (Fig. 5c). In both models, a surface *O atom that detaches and bonds to two Ru atoms is defined as a lattice oxygen atom (M–O–M). Since the Ru–O bond is longer than the Ru–M bond, the formation energy of lattice oxygen was determined through relaxation between surface *O and Ru atoms. The calculations revealed that the formation energy of the lattice oxygen in the AgRuIr alloy is 2.19 eV, which is significantly greater than the value of 1.39 eV observed for the RuIr alloy, consistent with the low oxophilicity of Ag (Fig. 5d). This increase in the formation energy effectively inhibits the incorporation of surface *O into the metallic lattice, thereby suppressing the conventional corrosion pathway through overoxidation.

### PEMWE performance

**Fig. 6:**
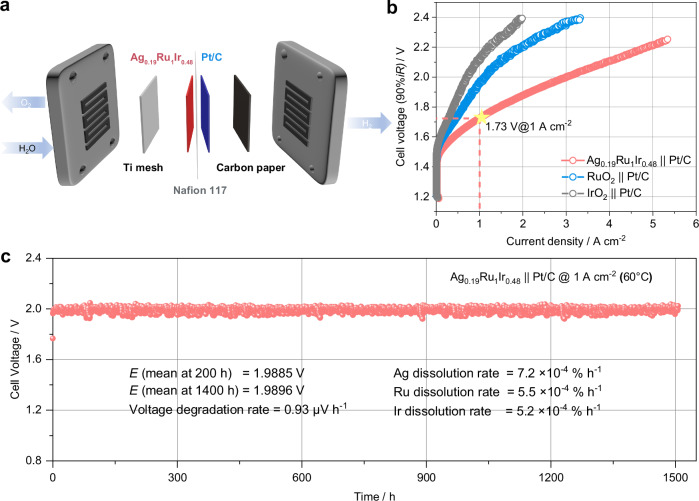
PEMWE performance of the Ag_0.19_Ru_1_Ir_0.48_ catalyst. **a** Schematic illustration of the PEM electrolyzer (proton exchange membrane type: Nafion 117). **b** Polarization curves of PEMWE in pure water at 60 °C using Ag_0.19_Ru_1_Ir_0.48_, RuO_2_, and IrO_2_ as the anode catalyst and commercial Pt/C as the cathode catalyst (*iR* compensation, 90%; scan rate, 10 mV s^−1^). Non-*iR* compensated curves, Fig. S54. **c** Chronopotentiometric curves of Ag_0.19_Ru_1_Ir_0.48_ | |Pt/C operated at 1 A cm⁻^2^ in pure water. Catalyst loadings: 1 mg cm^−2^ Ag_0.19_Ru_1_Ir_0.48_ at the anode, and 1 mg_Pt_ cm^−2^ commercial Pt/C at the cathode. Non-*iR* correction is applied. Source data are provided as a Source Data file.

To evaluate the practical applicability of our catalyst under realistic operating conditions, a PEMWE cell was assembled using Ag_0.19_Ru_1_Ir_0.48_ as the anode catalyst (loading: 1 mg cm^−2^), commercial Pt/C (40 wt.%, Sigma) as the cathode catalyst (loading:1 mg_Pt_ cm^−2^), and Nafion 117 as the proton exchange membrane (Figs. 6a, S47). As shown in Fig. 6b, the current–voltage (*I*–*V*) polarization curves (90% iR-corrected; temperature, 60 °C) reveal that the Ag_0.19_Ru_1_Ir_0.48_||Pt/C electrolyzer exhibits enhanced water electrolysis activity, achieving a current density of 1 A cm^−2^ at 1.73 V, a substantially lower cell voltage than the voltage required by RuO_2_||Pt/C (1.96 V) and IrO_2_||Pt/C (2.11 V) using commercial catalysts (PEMWE performance at 80 °C, Figs. S48, Table S16). The long-term operational durability was assessed at a constant current density of 1 A cm^−2^ (Fig. 6c). The cell voltage in this steady-state test (~2.0 V) is slightly higher than that observed in the transient polarization testing (1.73 V), which may arise from differences in surface intermediate coverage, time-dependent mass-transport phenomena (such as bubble accumulation), and variations in the uncompensated resistance of the distinct measurement systems employed. Remarkably, the cell voltage remained virtually constant throughout 1500 h of continuous operation, with a total voltage increase of only ~1 mV. The voltage degradation rate was calculated to be 0.93 μV h^−1^, which performs competitively with state-of-the-art catalysts reported in the literature (Table S17). This sustained stability highlights the high corrosion resistance of the AgRuIr alloy catalyst for the OER under practical PEMWE conditions.

Following the 1500 h durability test, ICP–MS was performed on the flowing water feed (volume fixed to 1 L; partial consumption of deionized water during electrolysis was compensated) to quantify the dissolution of Ru, Ir, and Ag from the metallic catalyst during long-term operation (Table S18). The concentrations of dissolved Ru, Ir, and Ag species were 5.1, 2.2, and 1.2 ppb, respectively. Based on these values, the dissolution rates of Ru, Ir, and Ag were calculated to be 5.5 × 10^−4^, 5.2 × 10^−4^, and 7.2 × 10^−4^ % h^−1^, respectively, corresponding to approximately 0.5–0.7% metal loss per 1000 h. The stability number (*S*-number), defined as the number of O_2_ molecules generated per Ru or Ir atom lost^58^, was calculated to be 2.3 × 10^8^ for Ag_0.19_Ru_1_Ir_0.48_, approximately six orders of magnitude higher than those of Ru_1_Ir_0.49_, RuO_2_, and IrO_2_ (Table S19). These results unequivocally demonstrate the corrosion resistance of the metastable AgRuIr alloy nanocages under the high anodic potentials of the OER, which accounts for their long-term operational stability in PEMWE testing.

To evaluate practical potential, the Ag_0.19_Ru_1_Ir_0.48_ alloy nanocages were subjected to a dynamic start-stop protocol in a PEMWE single cell, alternating between 1 A cm^−2^ and open-circuit potential to mimic renewable energy fluctuations (Figs. S49–S51, Table S20). The catalyst maintained stable cell voltage and high-frequency resistance, which confirms the structural and electrical integrity of the MEA. Post-cycling analysis via TEM and EDS mapping verified that the hollow morphology and atomic mixing were preserved, while XPS showed that the metals remained predominantly metallic with a low lattice oxygen content of 9.6%. These results provide a proof of concept for an oxidation-resistant metallic catalyst for the OER capable of withstanding non-steady-state operational stress.

## Discussion

In summary, we have developed a metallic catalyst for the OER, challenging the long-standing dominance of oxide-based catalysts. Our AgRuIr alloying strategy addresses the critical challenge of overoxidation faced by the conventional oxide catalysts, and shows even enhanced catalytic activity, contradicting traditional perceptions that metallic catalysts are inherently unsuitable for the OER. Therefore, this work establishes an alternative direction for designing efficient and enduring OER catalysts for PEMWE hydrogen production. The general principles uncovered here may be applicable to the rational design of metallic catalysts in a broad range of electrochemical processes that require high stability under harsh conditions, such as oxygen reduction in fuel cells.

## Methods

### Chemicals

Ruthenium (III) chloride (RuCl_3_, 99.9%, Sigma), iridium (III) chloride (IrCl_3_, 99.9%, Sigma), silver nitrate (AgNO_3_, 99.8%, Sigma), nickel nitrate hexahydrate (Ni(NO_3_)_2_, 99%, Sigma), cobalt carbonyl (Co_2_(CO)_8_, 98%, Sigma), chloroauric acid (HAuCl_4_, 99.99%, Sigma), cupric acetate monohydrate (Cu(CH_3_COO)_2_, 98%, Sigma), sodium borohydride (NaBH_4_, 98%, Aladdin), L-ascorbic acid (99.8%, Sigma), trisodium citrate (TSC, 99.5%, Sigma), polyvinylpyrrolidone (PVP, 99%, Sigma), oleic acid (85%, Aladdin), dioctylamine (98%, Sigma), sodium dodecyl sulfate (SDS, 99%, Sigma), ethylene glycol (EG, 98%, Aladdin), 1,2,3,4-tetrahydronaphthalene (95%, Acmec), acetonitrile (99.8%, Aladdin), isopropyl alcohol (99.7%, Aladdin), methanol (99.9%, Aladdin), ethanol (absolute, 99.8%, Aladdin), perchloric acid (HClO_4_, 70%, trace-metal grade, Aladdin), nitric acid (HNO_3_, 68%, Sinopharm), hydrochloric acid (HCl, 37%, Sinopharm), sulfuric acid (H_2_SO_4_, 98%, Sinopharm), Nafion solution (5 wt.%, Sigma), Nafion 117 membrane (DuPont), commercial Pt/C (40 wt.%, Sigma). All chemicals were used as purchased without further purification. Deionized water (18.2 MΩ·cm) was used for all aqueous solution preparations.

### Synthesis of metastable AgRuIr alloy nanocages

(1) Ag nanospheres (~29 nm) were first synthesized by a seeded growth method^75^. An Ag seed solution was first prepared by injecting 1.2 mL of ice-cold NaBH_4_ (0.1 M) into 30 mL of an aqueous solution containing 0.5 wt.% polyvinylpyrrolidone (PVP, *M*_W_ 360,000) and 1 mM AgNO_3_ under vigorous stirring. The seed solution was aged at 25 °C for 6 h before use. A growth solution was prepared by dissolving 3 mL of PVP (5 wt.%, *M*_W_ 360,000), 1 mL of acetonitrile, 400 μL of AgNO_3_ (0.1 M), and 300 μL of L-ascorbic acid (0.1 M) in 5 mL of H_2_O. Then, 16 mL of the Ag seed solution was injected into the growth solution under vigorous stirring. After stirring for 30 min, Ag nanospheres were recovered by centrifugation and redispersed in 43 mL of ethylene glycol (EG) (Ag: 1.28 mM, assuming 100% yield). (2) Next, RuIr grew on Ag nanospheres to form a core-shell structure. Typically, 7 mL of PVP (2.86 wt.%, *M*_W_ 360,000) in a vial was heated at 175 °C for 30 min to remove any moisture impurities. At 175 °C, 2 mL of the Ag nanospheres was added to the above solution. After that, 0.71 mL of RuCl_3_ (0.12 mM) was added dropwise into the solution, stirred for 1 h, and this procedure was repeated another 4 times to reach a total RuCl_3_ solution volume of 3.55 mL. Then, 0.67 mL of IrCl_3_ (0.08 mM) was added dropwise into the solution, stirred for 1 h, and this procedure was repeated another 3 times to reach a total IrCl_3_ solution volume of 2.68 mL. After stirring at 175 °C for 2 h, the solution was cooled down and stayed at 25 °C overnight. The resulting Ag@RuIr core-shell nanospheres were collected by centrifugation, washed with H_2_O three times, and redispersed in 3 mL of H_2_O. (3) Metastable AgRuIr alloy nanocages were synthesized by an etching-induced vacancy-diffusion-driven alloying mechanism. Typically, 3 mL of HNO_3_ (30 wt.%) was added to the Ag@RuIr dispersion (final HNO_3_ concentration: 15 wt.%). After 30 min at 25 °C, Ag_0.19_Ru_1_Ir_0.48_ alloy nanocages were collected by centrifugation, washed with H_2_O, and redispersed in H_2_O. When Ag-deficient Ag_0.06_Ru_1_Ir_0.47_ alloy nanocages were targeted, the etching was conducted in 6 mL of HNO_3_ (33.5 wt.%) at an elevated temperature of 80 °C for 48 h.

### Synthesis of RuIr/C nanoparticles

Carbon-supported RuIr alloy nanoparticles (RuIr/C, 41 wt%) were prepared by an impregnation method. Typically, 6.9 mL of RuCl_3_ (0.012 mM in ethanol) and 4.8 mL of IrCl_3_ (0.008 mM in ethanol) were mixed with 22.6 mg of carbon black (Vulcan XC-72). After drying at 25 °C, the powder was transferred to a tube finance and calcined at 500 °C for 5 h under a 5% H_2_ + 95% N_2_ atmosphere.

### Synthesis of CuRuIr alloy nanocages

Cu nanospheres (~95 nm) were first synthesized via a seeded growth method^76^. An Au seed solution was prepared by rapidly injecting 600 μL of ice-cold NaBH_4_ (0.01 M) into 40 mL of an aqueous solution containing 0.25 mM TSC and 0.25 mM HAuCl_4_ under vigorous stirring. The resulting seed solution was aged at 25 °C for 6 h before use. Subsequently, 520 μL of the Au seed solution was added to a mixture of 7 mL polyvinylpyrrolidone (PVP, 10 wt.%, *M*_W_ 40,000) and 200 μL Cu(CH_3_COO)_2_ (78.74 mM). Then, 4 mL of L-ascorbic acid (0.25 M) was rapidly injected under vigorous stirring. After 30 min, the Cu nanoparticles were collected by centrifugation and redispersed in 12.3 mL of EG, yielding a Cu concentration of 1.28 mM (assuming 100% yield). The CuRuIr alloy nanocages were subsequently synthesized following the same procedures used for AgRuIr alloy nanocages, except that 2 mL of Cu nanospheres was used in place of 2 mL of Ag nanospheres in the standard synthesis.

### Synthesis of NiRuIr alloy nanocages

Ni nanospheres (~97 nm) were first synthesized following previously reported protocols^77,78^. Typically, 10 mL of SDS (0.1 M), 1 mL of oleic acid (0.01 M in methanol), and 10 mL of Ni(NO_3_)_2_ (0.01 M) were dissolved in 80 mL of H_2_O. After vigorous stirring for 30 min, 27.6 mg (0.73 mmol) of NaBH_4_ was added. The mixture was stirred for 1 h, after which the Ni nanospheres were collected by centrifugation and redispersed in 78 mL of EG, affording a Ni concentration of1.28 mM (assuming 100% yield). Next, NiRuIr alloy nanocages were synthesized following the same procedures used for AgRuIr alloy nanocages, except that 2 mL Ni nanospheres was used in place of 2 mL of Ag nanospheres in the standard synthesis.

### Synthesis of CoRuIr alloy nanocages

Co nanospheres (~82 nm) were first synthesized by following a previously reported protocol^79^. Typically, 0.4 mL of oleic acid, 1 mL of dioctylamine, and 100 μL of Co_2_(CO)_8_ (0.3 M in 1,2,3,4-tetrahydronaphthalene) were dissolved in 36 mL of 1,2,3,4-tetrahydronaphthalene. The solution was refluxed at 210 °C for 1 h. The Co nanospheres were collected by centrifugation and redispersed in 23.4 mL of EG, yielding a Co concentration of 1.28 mM (assuming 100% yield). Next, CoRuIr alloy nanocages were synthesized following the same procedures as those for AgRuIr alloy nanocages, except that 2 mL of Co nanospheres was used in place of 2 mL of Ag nanospheres in the standard synthesis.

### Characterization

Atomic-resolution imaging and EELS mapping were carried out at 300 kV on a JEM-ARM300F (GrandARM) system, which utilized double spherical aberration correctors, ADF/BF/SAAF detectors, and an EELS spectrometer with a K2 direct detection camera. Complementary HRTEM and EDS mapping were executed at 200 kV using Talos F200X and JEOL JEM-F200 (HR) microscopes, while a Hitachi HT-7700 (100 kV) was utilized for low-magnification TEM imaging. XRD patterns of the materials were obtained on a Rigaku SmartLab powder X-ray diffractometer, which utilized a Cu *K*α radiation source. EXAFS was analyzed at beamline 13SSW of Shanghai Synchrotron Facility. The data were processed and normalized by the ATHENA software included in the IFEFFIT software package. For the fitting and simulation of EXAFS data, the ARTEMIS software and FEFF8.5 code were used. ICP-MS measurements were conducted on an Agilent 7800 (MS). TGA and DSC data were acquired at 10 °C min^−1^ on NETZSCH simultaneous thermal analyzer (STA 449 F3 Jupiter). Oxidation states were probed via XPS using an ESCALAB XI+ spectrometer, which utilized a monochromatic Al *K*α radiation source. The Ru 3 *d* and Ir 4 *f* XPS spectra were analyzed by nonsymmetric peak fitting^80,81^. In situ Raman spectra were obtained on a Renishaw inVia Qontor confocal Raman microscope.

### Electrochemical OER measurements

Electrochemical OER measurements were performed using a standard three-electrode system connected to a Shanghai Chenhua CHI760 electrochemical workstation. EIS was measured on an Autolab PGSTAT302 electrochemical workstation with a built-in EIS analyzer. A carbon cloth was used as the working electrode, and the carbon cloth was treated in a mixed acid [HCl (37 wt.%)/H_2_SO_4_ (98.3 wt.%) = 3:1 by volume] for 24 h before use. A carbon rod was used as the counter electrode. An Ag/AgCl (KCl: saturated) electrode was used as the reference electrode. The reference electrode was calibrated by determining the reversible hydrogen electrode (RHE) potential in H_2_-saturated 0.1 M HClO_4_. Two Pt foils were used as the working and counter electrodes, respectively, with an Ag/AgCl electrode serving as the reference. CV was performed at a scan rate of 1 mV s^−1^. The potential at which the CV curve intersects *j* = 0 corresponds to the potential difference between Ag/AgCl and RHE (measured value, −0.256 V). After calibration, the potential was converted using *E*_RHE_ = *E*_Ag/AgCl_ + 0.256 V, and the overpotential was calculated as *η* = *E*_RHE_ – 1.23 V.

O_2_-saturated 0.1 M HClO_4_ was used as the electrolyte, which should be freshly prepared before use (pH, Table S21). The electrolyte was stirred using a magnetic stirrer to facilitate the detachment of gas bubbles from the carbon cloth. Typically, 1 mg of metal catalyst was dispersed in 1 mL of a mixed solution (isopropyl alcohol/H_2_O/Nafion-117 (5 wt.%) = 1:1:0.004 by volume). The dispersion was sonicated for 1 h until a homogeneous ink was formed. A certain volume of the catalyst ink was drop-cast on carbon cloth and dried at 25 °C. The catalyst loading was 0.134 mg cm^−2^ (50 μL ink on 0.36 cm^2^ carbon cloth) for LSV measurements and chronopotentiometric testing at 10 mA cm^−2^, 0.536 mg cm^−2^ (193 μL ink on 0.36 cm^2^ carbon cloth) for chronoamperometric testing at 1.74 V, or 0.934 mg cm^−2^ for chronopotentiometric testing at 200 mA cm^−2^. Before electrocatalytic measurements, the working electrode was first activated by potential sweeping in 250 mL 0.1 M HClO_4_ from 1.0 to 1.6 V vs. RHE at a rate of 50 mV s^−1^ for 10 cycles. The LSV curves were obtained by potential sweep from 1.0 to 1.6 V vs. RHE at a rate of 10 mV s^−1^ at an *iR* compensation level of 95%. The catalytic stability of the catalysts was evaluated by chronoamperometric testing at 1.74 V vs. RHE and chronopotentiometric testing at 10 and 200 mA cm^−2^. EIS of the catalysts was performed at 1.46 V vs. RHE from 0.1 Hz to 100 kHz, and the results were presented in the form of a Nyquist plot.

### PEMWE testing

In a typical PEMWE setup, a proton exchange membrane (Nafion 117) was used as the electrolyte. The Nafion 117 membrane (1.5 cm × 1.5 cm, thickness, 183 μm) was pretreated as follows: (1) hydrothermal treatment in 5% H_2_O_2_ at 80 °C for 1 h; (2) rinsing in deionized water at 80 °C for 1 h; (3) hydrothermal treatment in 0.5 M H_2_SO_4_ at 80 °C for 1 h; (4) rinsing in deionized water at 80 °C for 1 h; and (5) soaking in 0.5 M H_2_SO_4_ at room temperature for 24 h, followed by natural air-drying prior to use. The Ag_0.19_Ru_1_Ir_0.48_ alloy nanocages were spray-coated on one side (spraying area: 1 cm × 1 cm) of the Nafion 117 at a loading of 1 mg cm^−2^ to serve as the OER anode, and commercial Pt/C (40 wt%, Sigma) was spray-coated onto the other side at a loading of 1 mg_Pt_ cm^−2^ to serve as the HER cathode. The catalyst was sprayed using an ultrasonic precision sprayer (Siansonic UC320). After drying at 25 °C, the anode was covered with a Ti mesh and the cathode was covered with carbon paper, both serving as gas diffusion layers. The electrodes were then pressed at 25 °C under 0.5 MPa using a standard hot-press machine (120 °C). The current-voltage curve of the PEMWE device was measured on a Gamry electrochemical workstation (Gamry Interface 5000E). The chronopotentiometry curve of the PEMWE cell was determined using a battery testing system (Neware BTS-5V6A). During PEMWE testing, the active area is 1 × 1 cm^−2^ and 60 °C DI water was fed to the anode at a flow rate of 100 mL min^−1^ by a peristaltic pump.

### Catalyst dissolution analysis in half cells

After stability testing, the electrolyte was collected, volumetrically fixed, and analyzed by ICP-MS to determine the quantity of dissolved metals in the electrolyte. The surface carbon on the carbon rod (counter electrode) was scraped off and collected. The carbon powder was calcined at 650 °C in air for 4 h, microwave digested at 180 °C in concentrated HNO_3_ (67 wt.%) for 2 h, and analyzed by ICP-MS to determine the quantity of metals redeposited on the counter electrode. The dissolved metals were calculated by summing the quantities in the electrolyte and redeposited on the carbon rod. To quantify the catalysts retained on the carbon cloth, the carbon cloth was ultrasonicated in HNO_3_ (67 wt.%) to collect the metal catalysts. Then, the carbon cloth was calcined at 650 °C in air for 4 h and microwave digested in HNO_3_ (67 wt.%). The two HNO_3_ solutions were merged and analyzed by ICP-MS. The quantity of peeled catalysts from the working electrode was calculated by mass balance.

### Catalyst dissolution analysis in PEMWE

The metal dissolution rate in PEMWE was determined by quantifying the dissolved metal species in the flowing water feed using ICP-MS. The voltage degradation rate was evaluated via chronopotentiometric testing of the PEMWE device at 1 A cm^−2^. Specifically, it was obtained by dividing the difference between the average electrolyzer voltages at 200 h (mean of 12,000 data points collected from 150 to 250 h; acquisition interval: 30 s) and 1400 h (mean of 12,000 data points from 1350 to 1450 h) by the elapsed time of 1200 h. The stability number (*S-*number) was calculated according to equation^58^: *S*-number = *n*_O2_/[*n*_Ru(dissolved) +_ *n*_Ir(dissolved)_]. Here, *n*_O2_ is the quantity of oxygen evolved over a given period, given by *n*_O2_ = *jAt*/(*nF*), where *j* is the current density, *A* is the electrode area, *t* is the period of time, *n* is the number of electron transfer for the formation of one O_2_ molecule (*n* = 4), and *F* is the Faraday constant. *n*_Ru(dissolved)_ and *n*_Ir(dissolved)_ are the amounts of dissolved Ru and Ir species measured by ICP-MS.

### DFT calculations

An Ag_8_Ru_38_Ir_18_ alloy structure was generated by the Alloy-Theoretic Automated Toolkit (ATAT) code (https://alum.mit.edu/www/avdw/atat/). The Ru_43_Ir_21_ alloy structure was randomly generated with lower energy based on the HCP Ru (101) facet structure and experimental XRD data of Ru_1_Ir_0.49_. The two surfaces were modeled using slab geometries with a four-layer (4 × 4) unit cell. The OER steps follow the adsorbate evolution mechanism. The overpotential of the OER was calculated by *η =  ΔG*_*rds*_*/e* – 1.23 V, where *ΔG*_*rds*_ is the free reaction energy of the rate-determining step. DFT calculations were performed with the Vienna ab initio simulation package (VASP) at a version of 5.4.4 with the projector augmented wave (PAW) method and a plane wave basis set^82–84^. An energy cutoff of 400 eV accounts for core-valence interactions. The approach employed was DFT utilizing generalized gradient approximations (GGAs) from the Perdew−Burke−Ernzerhof (PBE) functional^85^. D3 van der Waals correction was used in all calculations^86^. The structures of all reaction intermediates during the OER were fully relaxed until the force on each atom is less than 0.01 eV Å^−1^. The self-consistent electronic step was considered converged when the change of total energy and the change of eigenvalues between two steps were both smaller than 1 × 10^−6^ eV. For the optimization of the crystals, reciprocal space was sampled using the 3 × 3 × 1 Monkhorst-Pack sampled *k*-points, with the bottom two layers of atoms fixed. Free energies for all structures were computed using standard statistical mechanics formulae that account for translation, rotational, vibrational, and electronic degrees of freedom^87,88^. The Gibbs free energy was calculated by *G* = *E*_0_ + *E*_sol_ + *E*_ZPE_ – *TS*, where the total energies (*E*_0_) of each adsorbed state and gas molecules were yielded from DFT calculation. Zero-point energy (*E*_ZPE_) and the entropy (*S*) correction were also obtained by using the VASPKIT code for a postprocessing of the VASP calculated data^89^. Solvation energy (*E*_sol_) was obtained by implicit solvation model correction^90–92^.

## Supplementary information

Supplementary Information

Peer Review File

Description of Additional Supplementary Files

Supplementary Data 1

## Source data

Source Data

## Data Availability

All data are available in the main text or the supplementary materials. Source data are provided with this paper.
