# Supplementary material for: Oxidation-resistant AgRuIr alloy nanocages for efficient and enduring oxygen evolution in proton exchange membrane electrolysis
Source: Nat Commun. 2026 Apr 15;17:5195. doi: 10.1038/s41467-026-71943-6 (PMC13254247; doi:10.1038/s41467-026-71943-6)
Supplement: Supplementary file 1 — Supplementary Information [file 41467_2026_71943_MOESM1_ESM.pdf]

Supplementary Information

Oxidation-Resistant AgRuIr Alloy Nanocages for Efficient and Enduring Oxygen Evolution in Proton Exchange Membrane Electrolysis

Xiaoxiao Wang[1†], Peiping Yu[2,3†], Moxuan Liu[1†], Lei Wang[2,3], Fanfan Shang,[4] Fangpu Zhang[1], Zhaojun Liu[1], Yuke Bai[1], Kai Liu[1], Liang Zhang[2,3], Shengchun Yang[4], Qing Zhang[5,6], Tao Cheng[2,3], and Chuanbo Gao[1]*

[1]Sate Key Laboratory of Multiphase Flow in Power Engineering, Frontier Institute of Science and Technology, Xi'an Jiaotong University, Xi'an 710049, China.

[2]Institute of Functional Nano & Soft Materials (FUNSOM), Jiangsu Key Laboratory for Carbon-Based Functional Materials and Devices, Soochow University, Suzhou 215123, China.

[3]Jiangsu Key Laboratory of Advanced Negative Carbon Technologies, Soochow University, Suzhou 215123, China.

[4]MOE Key Laboratory for Nonequilibrium Synthesis and Modulation of Condensed Matter, School of Physics, Xi'an Jiaotong University, Xi'an 710049, China.

[5]Center for High-resolution Electron Microscopy (CħEM), School of Physical Science and Technology, ShanghaiTech University, Shanghai 201210, China.

[6]Shanghai Key Laboratory of High-resolution Electron Microscopy, ShanghaiTech University, Shanghai 201210, China.

[†]X.W., P.Y., and M.L. contributed equally to this work.

*Corresponding author. Email: gaochuanbo@mail.xjtu.edu.cn

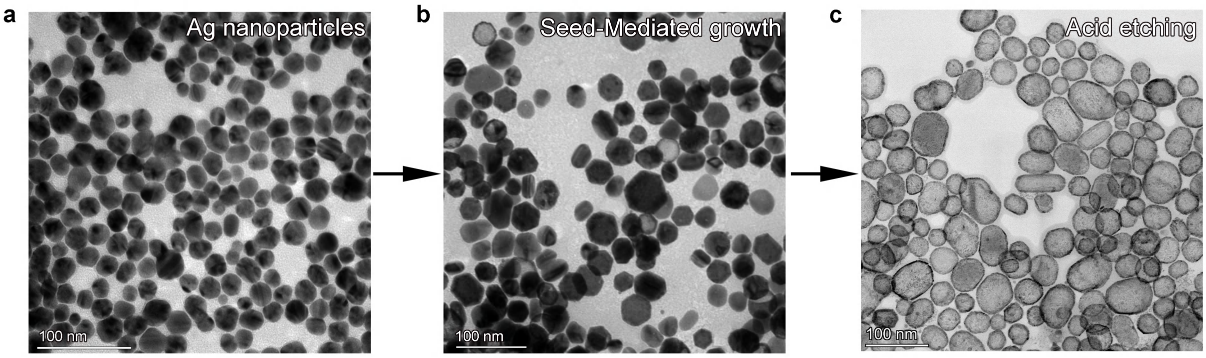

Fig. S1. Synthesis of metastable AgRuIr alloy nanocages. (a) TEM image of Ag nanoparticles. **(b)** TEM image of Ag@RuIr core-shell nanocrystals. **(c)** TEM image of $Ag_{0.19}Ru_1Ir_{0.48}$ alloy nanocages.

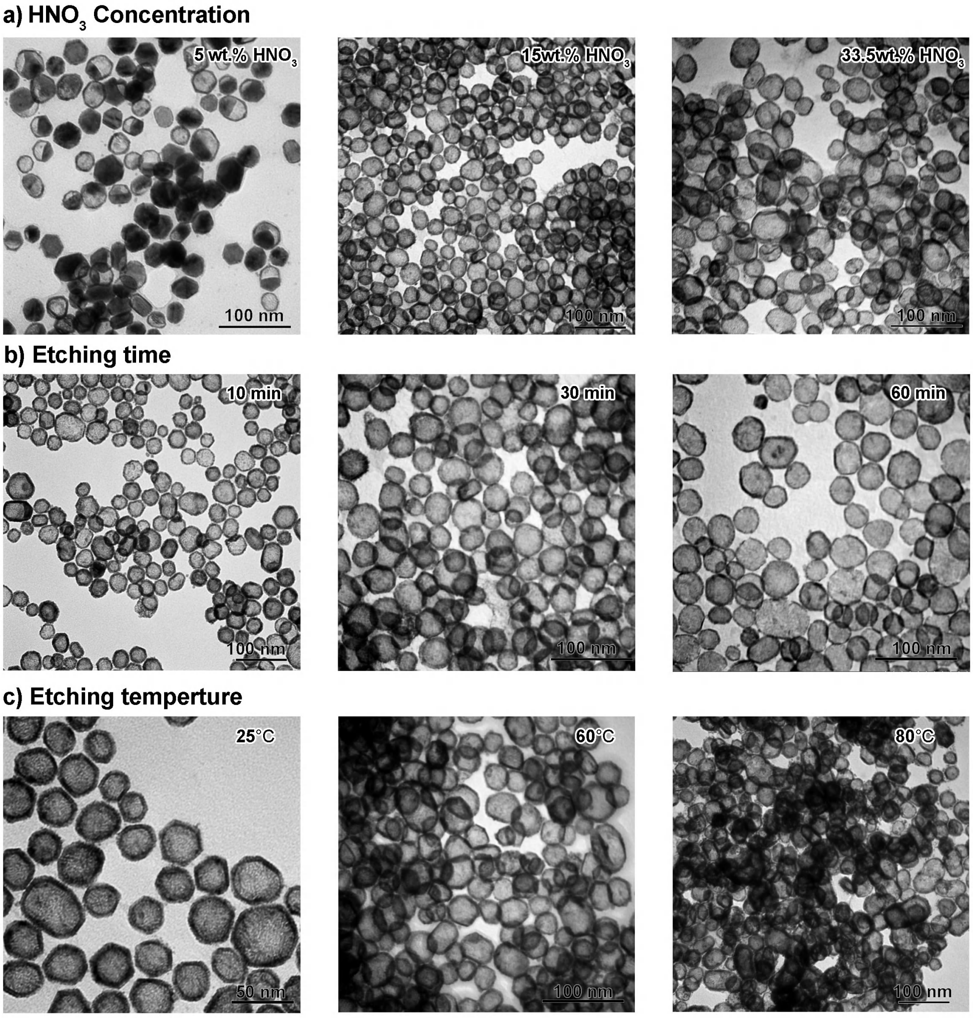

Fig. S2. Optimization of etching conditions. TEM images of products obtained by varying key etching parameters: (**a**) HNO_3 concentration, (**b**) etching duration, and (**c**) etching temperature. The images illustrate the structural evolution from incomplete core removal to excessive shell thinning.

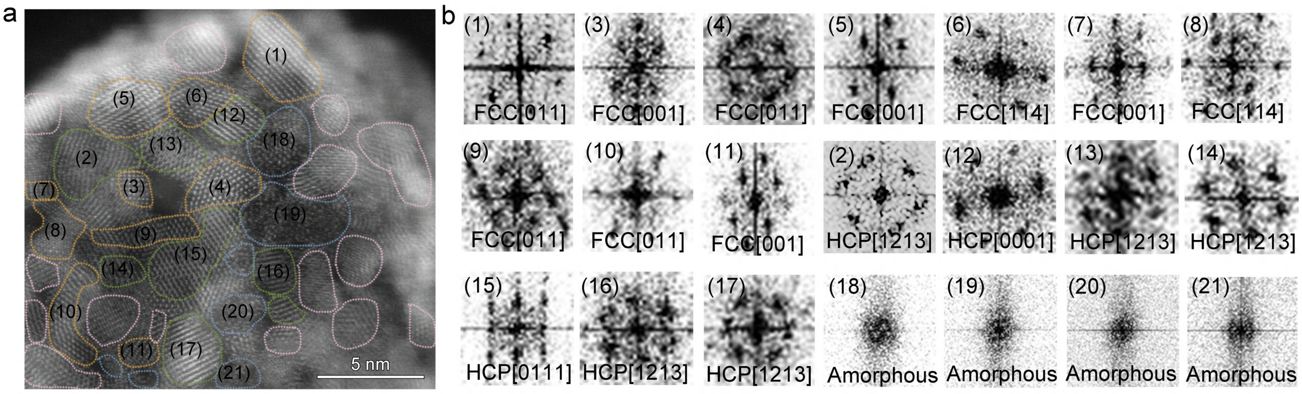

Fig. S3. Phase analysis of crystallites in $Ag_{0.19}Ru_1Ir_{0.48}$ alloy nanocages. (a) HAADF-STEM image of a representative nanocage. The yellow, green, blue, and pink dotted lines delineate the FCC, HCP, amorphous, and undefined (structure not resolved) crystallites, respectively. **(b)** FFT diffraction patterns corresponding to the FCC, HCP, and amorphous crystallites as labeled in (a).

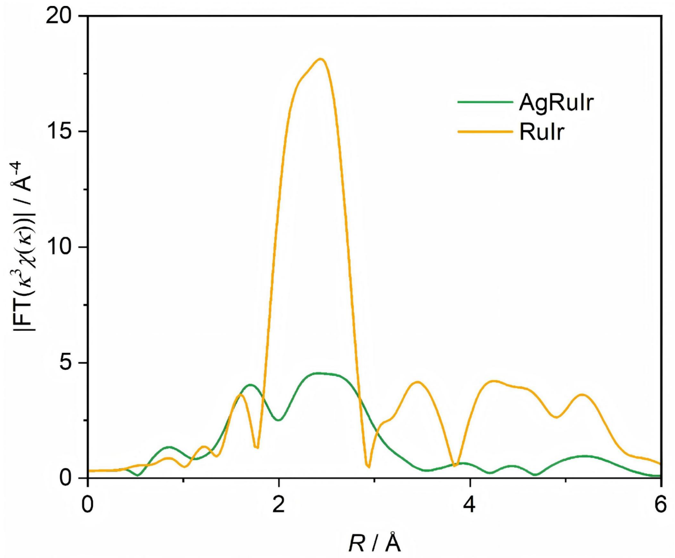

Fig. S4. Ir L_3-edge FT-EXAFS of the AgRuIr alloy nanocages and the RuIr alloy nanoparticles. *R*, radial distance. Source data are provided as a Source Data file.

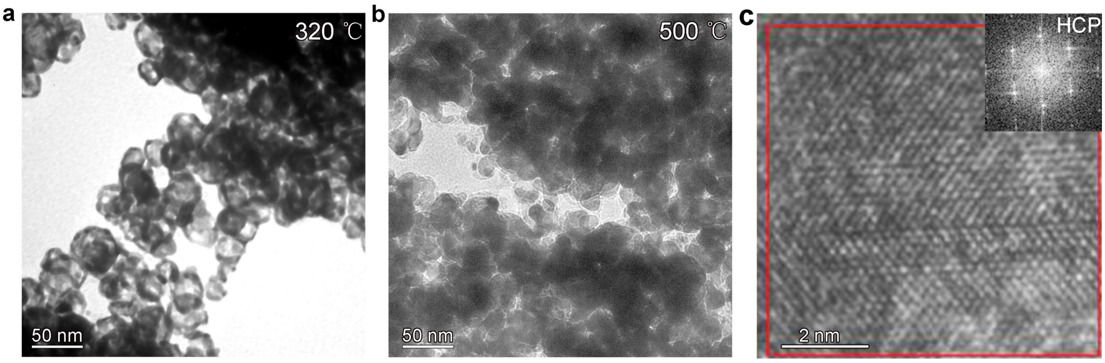

Fig. S5. Structure of $Ag_{0.19}Ru_1Ir_{0.48}$ after calcination. (a) TEM image of $Ag_{0.19}Ru_1Ir_{0.48}$ after calcination at 320 °C. **(b)** TEM image of $Ag_{0.19}Ru_1Ir_{0.48}$ after calcination at 500°C. **(c)** HRTEM image of $Ag_{0.19}Ru_1Ir_{0.48}$ after calcination at 500 °C. Inset: Fourier diffraction pattern, showing the HCP crystal phase.

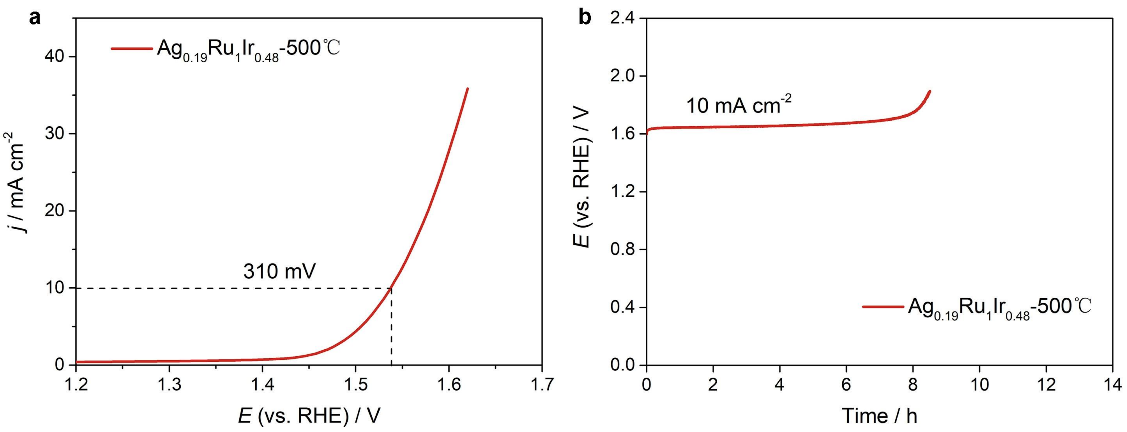

Fig. S6. OER performance of the phase-separated Ru-Ir/Ag catalyst obtained by annealing $Ag_{0.19}Ru_1Ir_{0.48}$ alloy nanocages at 500 °C. (**a**) LSV curves in O_2-saturated 0.1 M $HClO_4$ (pH = 1 ± 0.01) at 25 °C, scan rate: 10 mV s^{-1}. *iR* compensation level: 95%. Catalyst loading: 0.134 mg cm^{-2}. (**b**) Chronopotentiometric curve of the catalyst at 10 mA cm^{-2} 0.1 M $HClO_4$ (pH = 1 ± 0.01) at 25 °C. Source data are provided as a Source Data file.

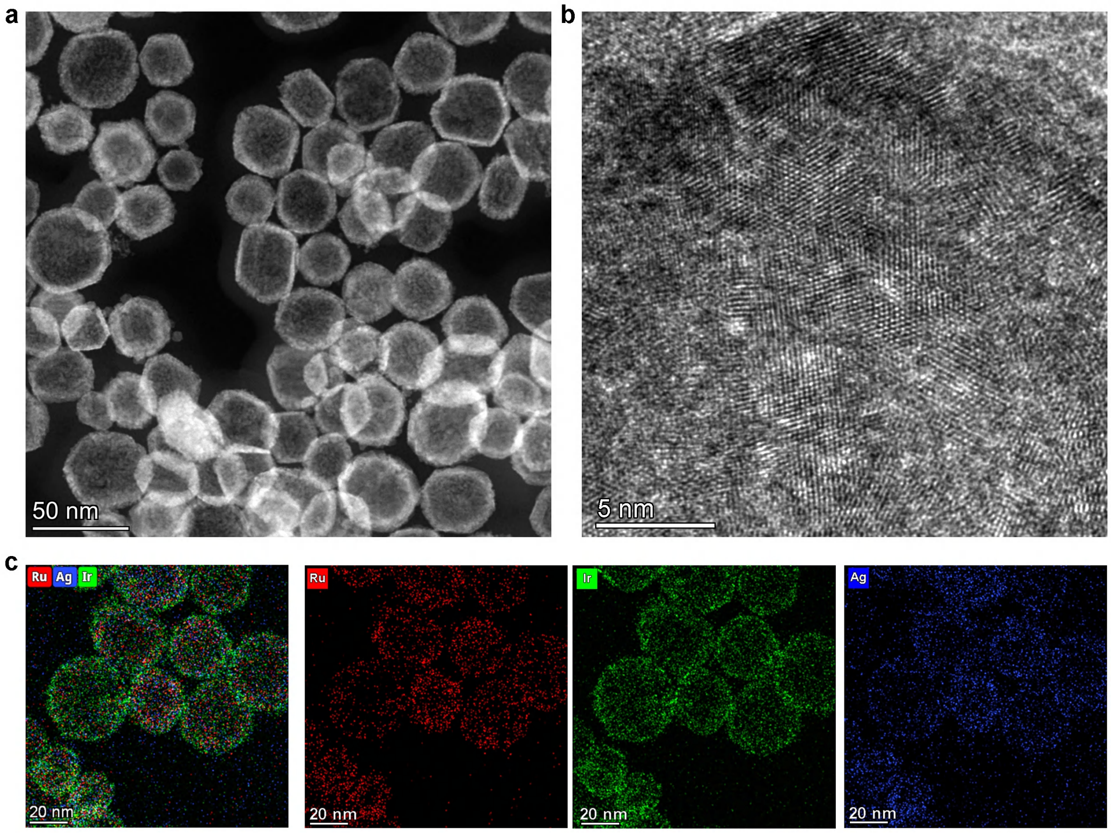

Fig. S7. Characterization of Ag-deficient $Ag_{0.06}Ru_{1}Ir_{0.47}$ alloy nanocages. (a, b) HAADF-STEM and HRTEM images. **(c)** EDS elemental mappings.

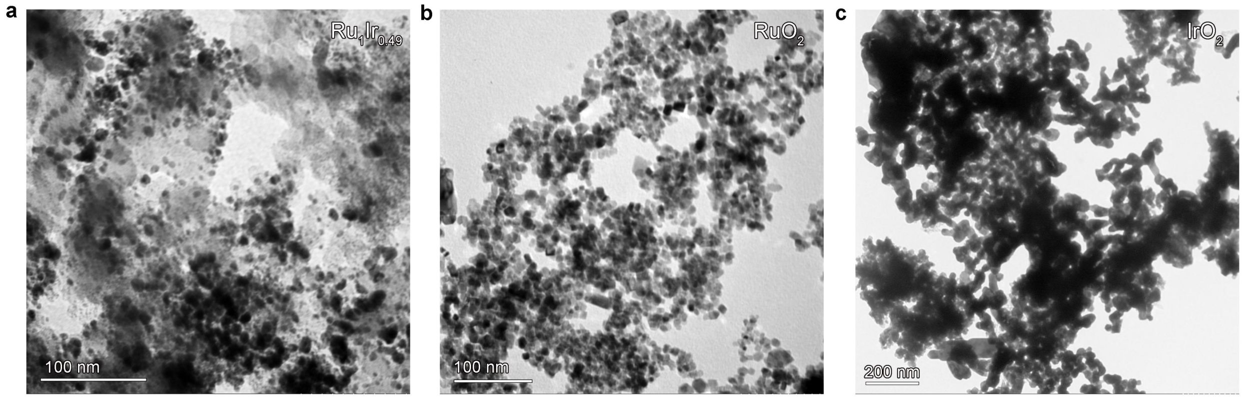

Fig. S8. TEM images of control catalysts for the OER. (a) Carbon-supported $Ru_1Ir_{0.49}$ alloy nanoparticles synthesized by impregnation method. **(b)** Commercial RuO_2. **(c)** Commercial IrO_2.

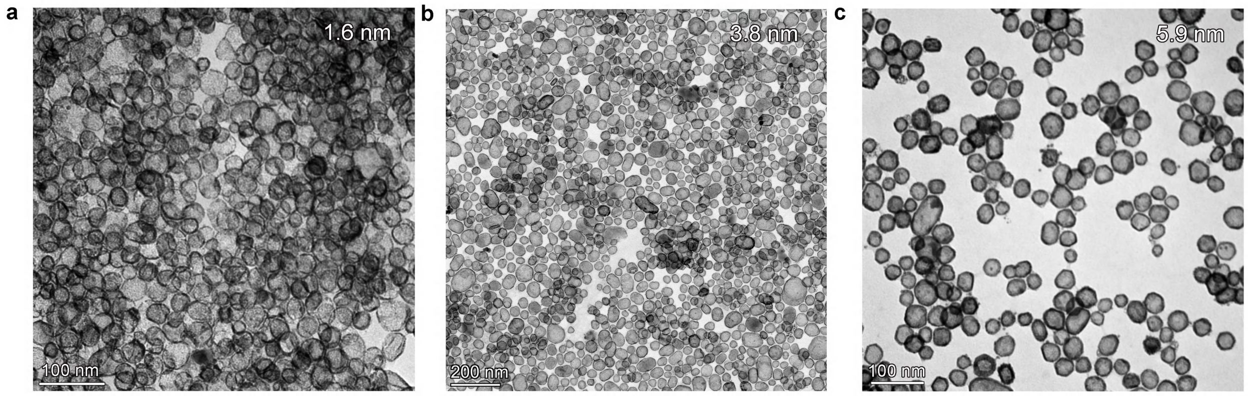

Fig. S9. Tuning of the shell thickness of AgRuIr alloy nanocages. (a–c) TEM images of nanocages with average shell thicknesses of 1.6, 3.8, and 5.9 nm, respectively. The shell thickness was modulated by controlling the (Ru + Ir)/Ag molar ratio in the synthesis precursor, which was set to 0.30, 0.59, and 0.81, respectively.

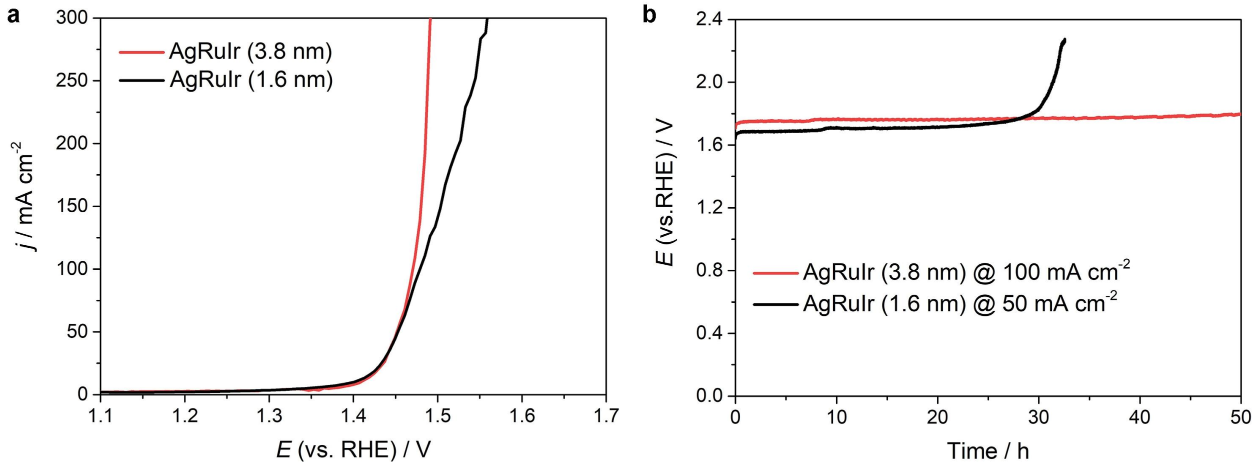

Fig. S10. OER performance of AgRuIr alloy catalysts with different shell thicknesses. (a) LSV curves in O_2-saturated 0.1 M $HClO_4$ (pH = 1 ± 0.01) at 25 °C, scan rate: 10 mV s^{-1} with 95% *iR* compensation. **(b)** Chronopotentiometric testing in 0.1 M $HClO_4$ (pH = 1 ± 0.01) at 25 °C. Catalyst loading: 0.134 mg cm^{-2}. Source data are provided as a Source Data file.

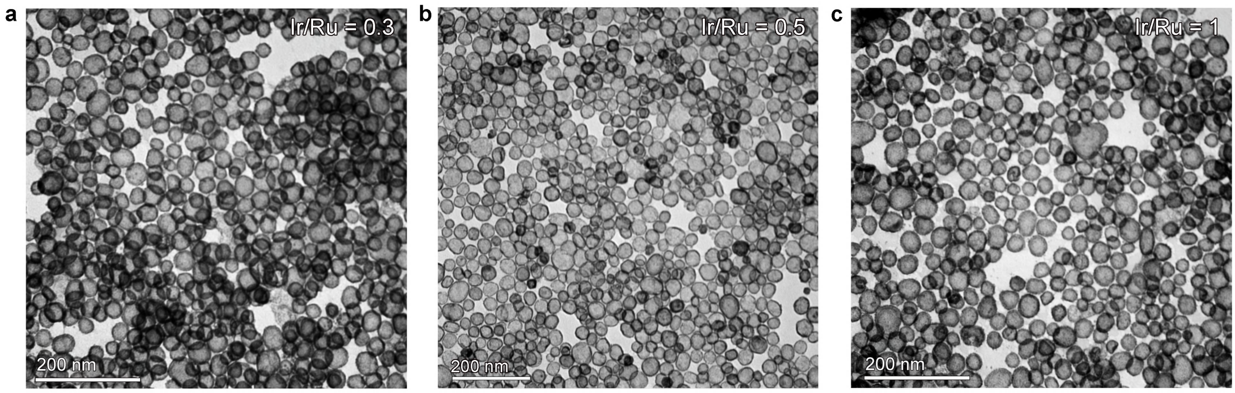

Fig. S11. Tuning of Ir/Ru ratio of AgRuIr alloy cages. (a-c) TEM images of nanocages with Ir/Ru ratio of 0.3, 0.5 and 1, respectively.

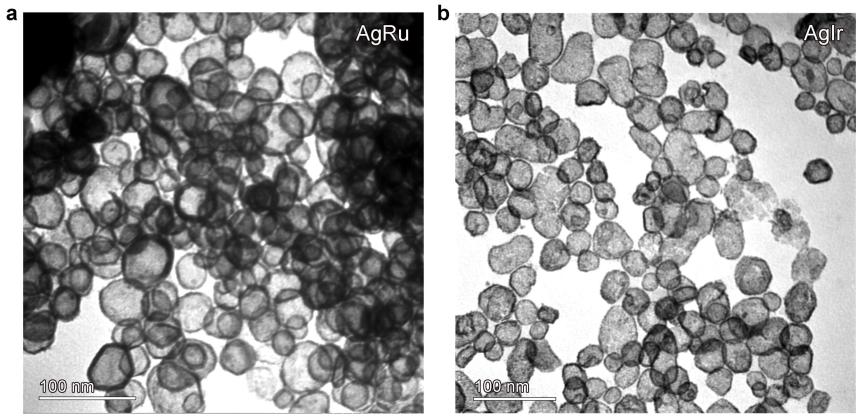

Fig. S12. TEM images of AgRu and AgIr alloy nanocages. (a) AgRu. **(b)** AgIr.

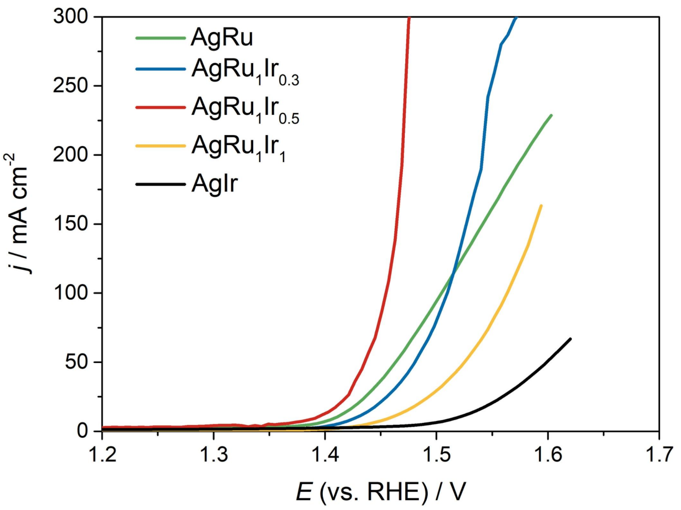

Fig. S13. OER LSV curves of AgRuIr alloy catalysts with different Ir/Ru ratios. Curve in O_2-saturated 0.1 M $HClO_4$ (pH = 1 ± 0.01) at 25 °C, scan rate: 10 mV s^{-1} with 95% *iR* compensation. Source data are provided as a Source Data file.

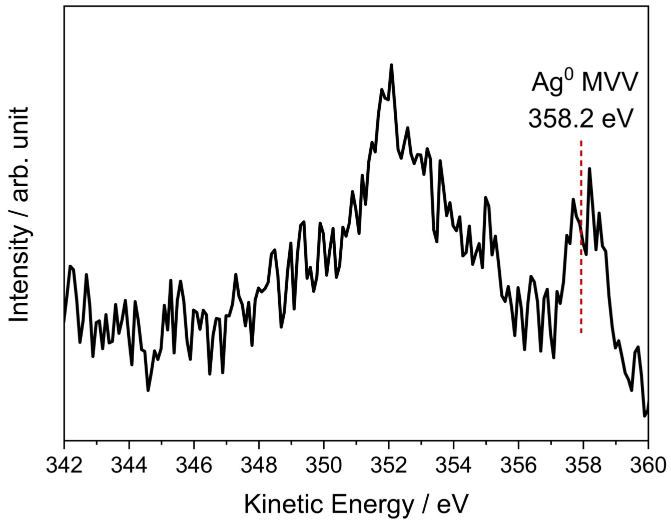

Fig. S14. Auger electron spectroscopy of Ag in $Ag_{0.19}Ru_1Ir_{0.48}$ alloy nanocages. The spectrum was acquired after chronoamperometric testing at 1.74 V for 2 h. The Ag MVV peak is observed at kinetic energy of 358.2 eV, corresponding to Ag^0 (reference: Ag^+ MVV peak usually appears in the range of 355–357 eV). Source data are provided as a Source Data file.

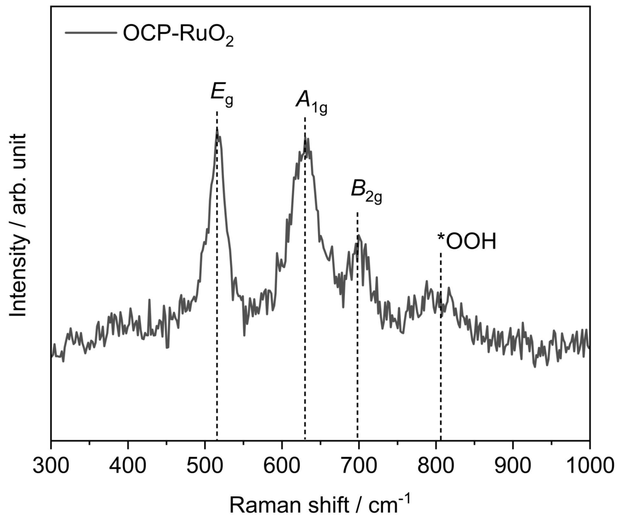

Fig. S15. In situ Raman spectrum of commercial RuO_2 at the open-circuit potential in 0.1 M $HClO_4$. Source data are provided as a Source Data file.

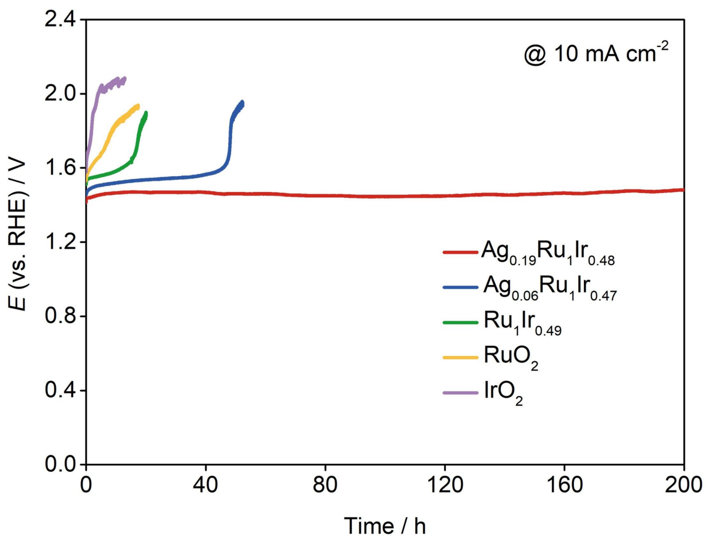

Fig. S16. Chronopotentiometric curve of the $Ag_{0.19}Ru_1Ir_{0.48}$ catalysts at 10 mA cm^{-2}. Curves in O_2-saturated 0.1 M $HClO_4$ (pH = 1 ± 0.01) at 25 °C. Control catalysts: $Ag_{0.06}Ru_1Ir_{0.47}$, $Ru_1Ir_{0.49}$, RuO_2, and IrO_2. Catalysts loading: 0.134 mg cm^{-2}. Non-*iR* correction is applied. Source data are provided as a Source Data file.

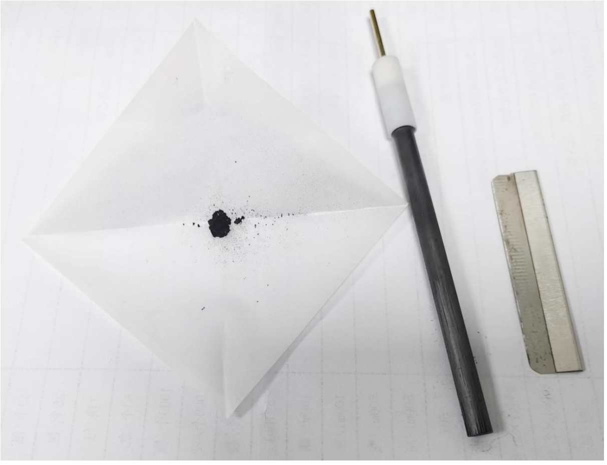

Fig. S17. Carbon powder scraped from the counter electrode (carbon rod). The carbon powder was analyzed by ICP-MS to determine the quantity of dissolved metals that were redeposited on the counter electrode.

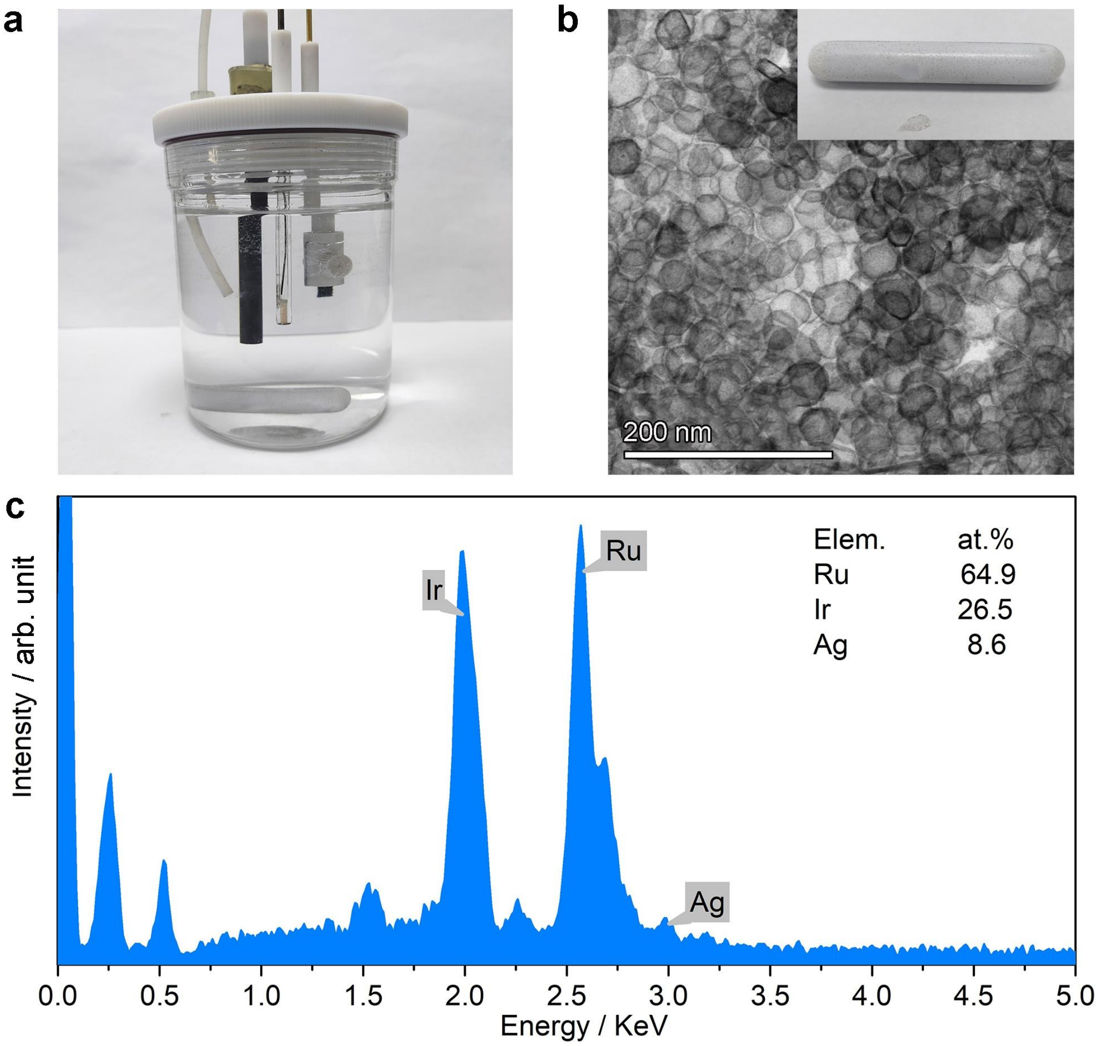

Fig. S18. Characterization of $Ag_{0.19}Ru_1Ir_{0.48}$ peeled from the working electrode after chronopotentiometric testing at 200 mA cm^{-2} for 1000 h. (a) Setup for electrocatalytic testing. **(b)** Photograph of the magnetic stirrer after catalysis (inset), showing a black material deposited on it. The black material was analyzed by TEM, revealing the presence of metal nanocages. **(c)** EDS spectrum of the black material revealed that it is composed of Ag, Ru, and Ir. These results suggest that the AgRuIr catalysts were partially peeled from the carbon cloth (working electrode) during the long-term catalysis. Source data are provided as a Source Data file.

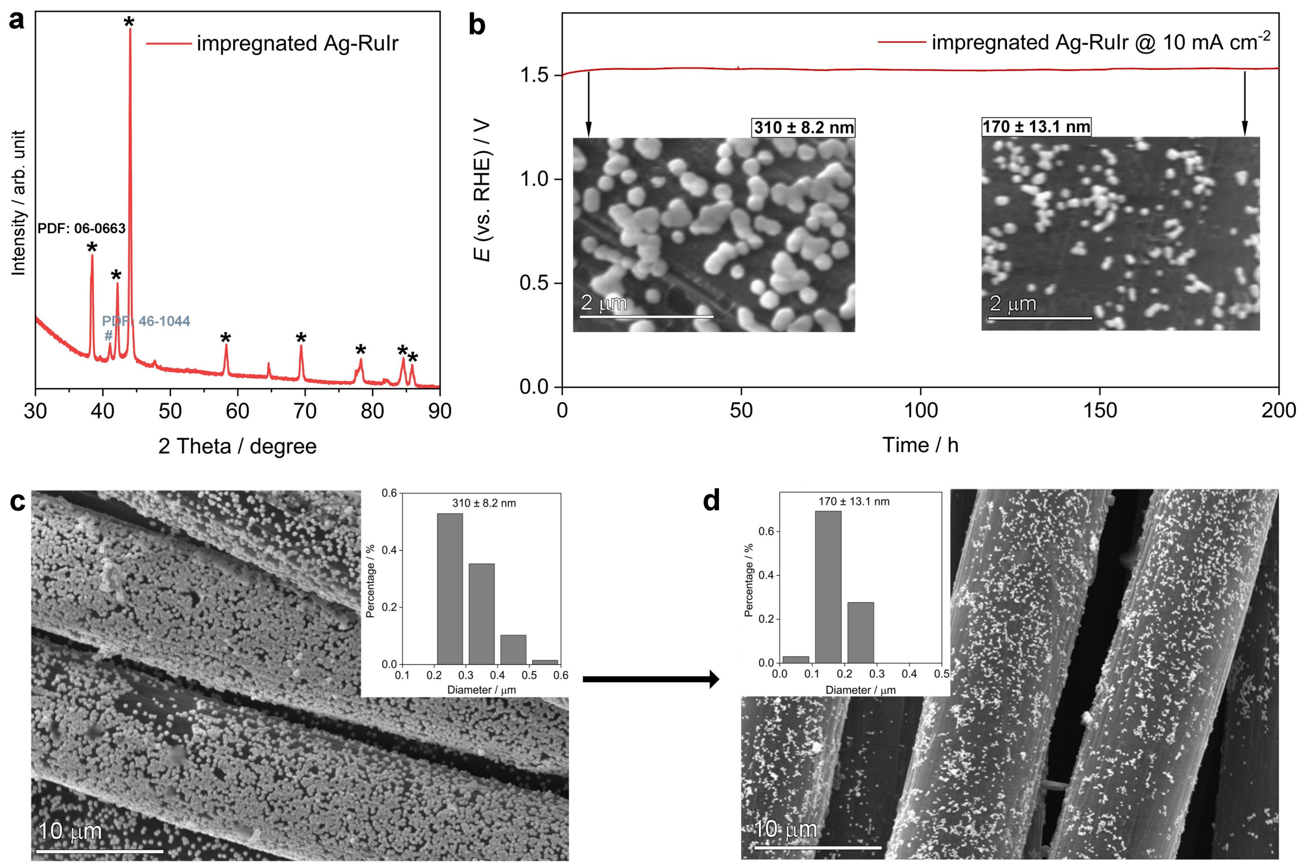

Fig. S19. Morphological and size change of a phase-separated Ag-RuIr catalyst during OER. The catalyst was synthesized via impregnation followed by annealing in a H_2 atmosphere. (**a**) XRD pattern of the catalyst, showing distinct signals from segregated Ag and RuIr phases. (**b**) Chronopotentiometric curve recorded at 10 mA cm^{-2} in O_2-saturated 0.1 M $HClO_4$ (pH = 1 ± 0.01) at 25 °C. Non-*iR* correction is applied. Inset: SEM images of the catalyst nanoparticles before and after testing. (**c, d**) Low-magnification SEM images of the Ag-RuIr catalyst before and after chronopotentiometric testing. Inset: Corresponding particle size distributions. While the potential remained relatively stable, the average particle size decreased from 310 nm to 170 nm, indicating significant oxidative dissolution. These results also indicate that average particle size can serve as an indicator of metal loss during the OER process. Source data are provided as a Source Data file.

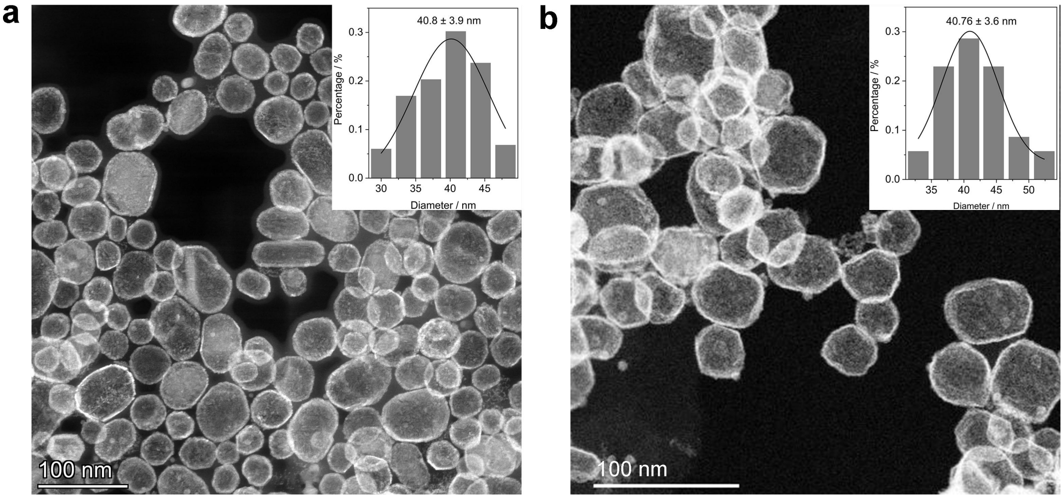

Fig. S20. Morphological and size change of the AgRuIr alloy nanocages during OER. (**a**) HAADF-STEM image of the as-synthesized nanocages. (**b**) HAADF-STEM image of the nanocages after chronopotentiometric testing at 200 mA cm^{-2} for 200 h. Inset: diameter histograms of nanocages. Source data are provided as a Source Data file.

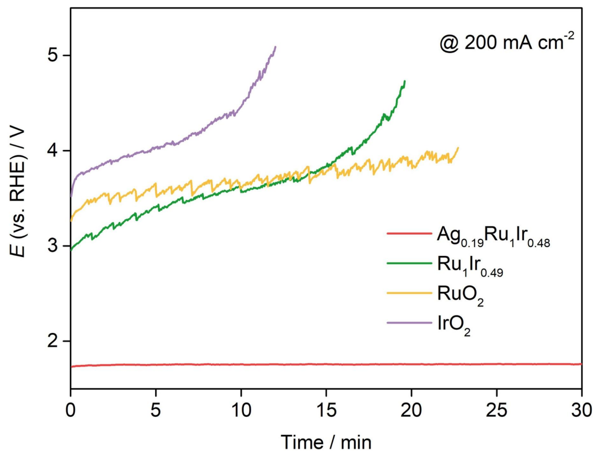

Fig. S21. Chronopotentiometric curves of $Ag_{0.19}Ru_1Ir_{0.48}$, $Ru_1Ir_{0.49}$, RuO_2, and IrO_2 at 200 mA cm^{-2}. Curve in O_2-saturated 0.1 M $HClO_4$ (pH = 1 ± 0.01) at 25 °C, catalysts loading: 0.934 mg cm^{-2}. Non-*iR* correction is applied. Source data are provided as a Source Data file.

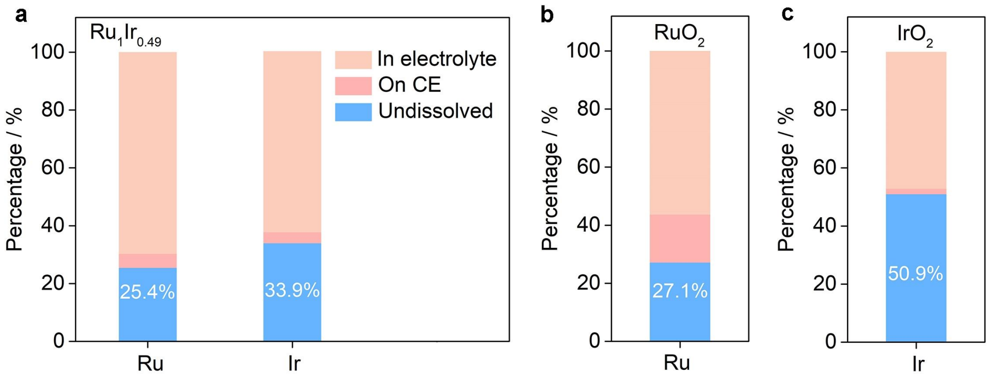

Fig. S22. Ratios of dissolved metals from $Ru_1Ir_{0.49}$, RuO_2, and IrO_2 during chronopotentiometric testing at 200 mA cm^{-2}. Catalysts loading: 0.934 mg cm^{-2}. **(a)** Ratios of dissolved Ru and Ir from $Ru_1Ir_{0.49}$ after the chronopotentiometric testing for 25 min. **(b)** Ratios of dissolved Ru from RuO_2 after the chronopotentiometric testing for 12 min. **(c)** Ratios of dissolved Ir from IrO_2 after the chronopotentiometric testing for 20 min. Source data are provided as a Source Data file.

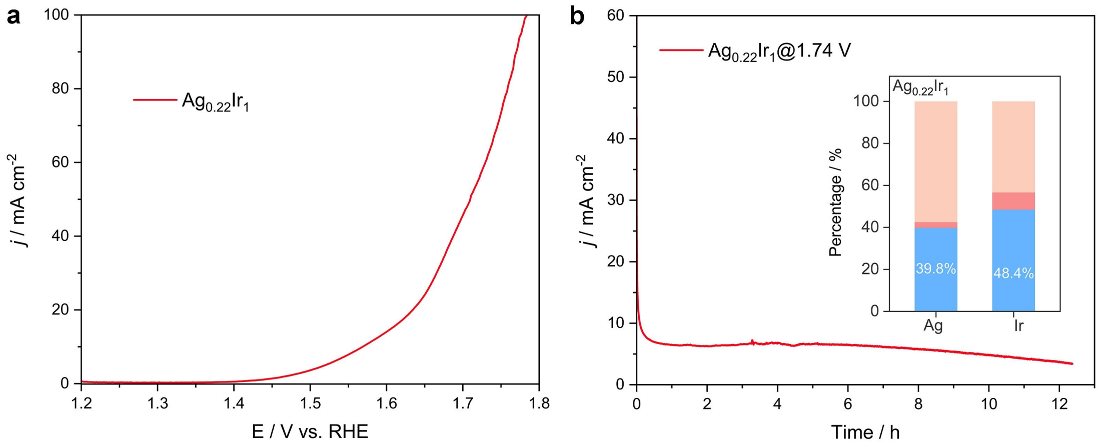

Fig. S23. Catalytic activity and stability of the AgIr alloy catalyst in acidic OER. (**a**) LSV curve in O_2-saturated 0.1 M $HClO_4$ (pH = 1 ± 0.01) at 25 °C, scan rate: 10 mV s^{-1} with 95% *iR* compensation. (**b**) Chronoamperometric curve recorded at 1.74 V in 0.1 M $HClO_4$ (pH = 1 ± 0.01) at 25 °C. Non-*iR* correction is applied. Inset: Percentages of dissolved Ag and Ir following chronoamperometric testing, as determined by ICP-MS. The yellow, pink, and blue bars represent the percentages of metals dissolved in the electrolyte, redeposited on the counter electrode, and remaining undissolved, respectively. Source data are provided as a Source Data file.

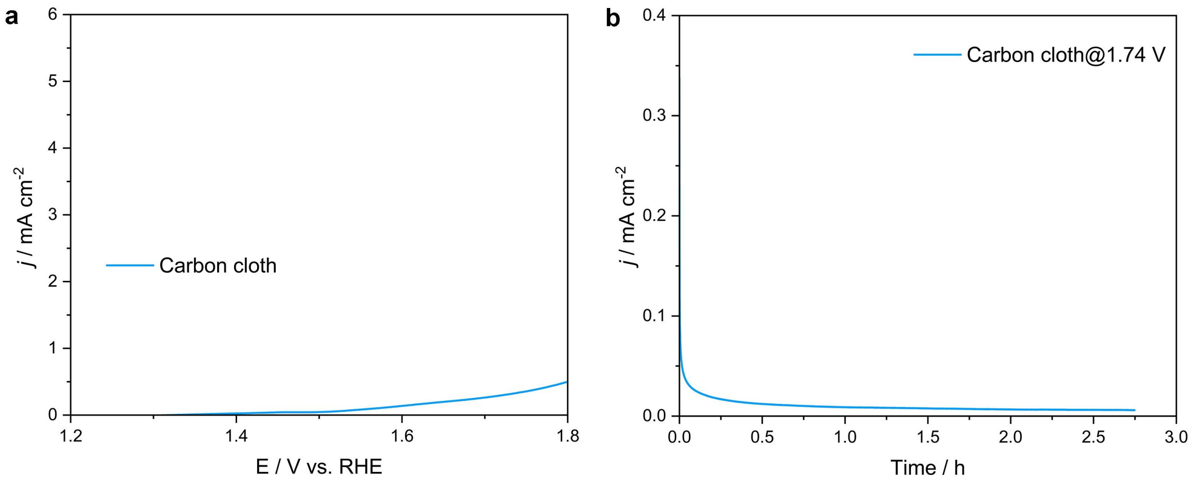

Fig. S24. Electrochemical OER activity of the carbon cloth substrate. (**a**) LSV curve in O_2-saturated 0.1 M $HClO_4$ (pH = 1 ± 0.01) at 25 °C, scan rate: 10 mV s^{-1} with 95% *iR* compensation. (**b**) Chronoamperometric curve recorded at 1.74 V in 0.1 M $HClO_4$ (pH = 1 ± 0.01) at 25 °C. Non-*iR* correction is applied. The minimal current response confirms the negligible contribution of the substrate to the measured catalytic activity. Source data are provided as a Source Data file.

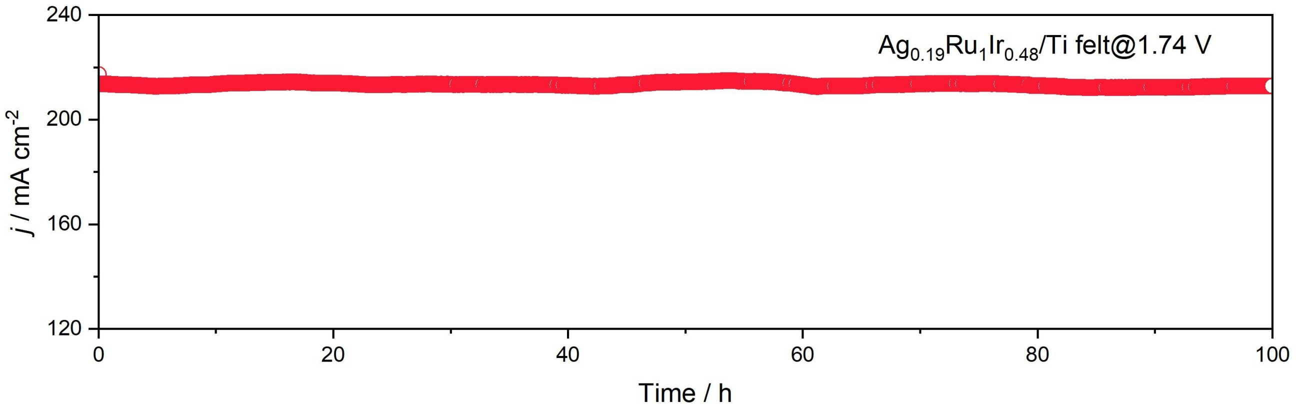

Fig. S25. Electrocatalytic OER performance of the $Ag_{0.19}Ru_1Ir_{0.48}$ alloy nanocages on a titanium fiber felt substrate. Chronoamperometric curve recorded at 1.74 V in 0.1 M $HClO_4$ (pH = 1 ± 0.01) at 25 °C with a catalyst loading of ~0.5 mg cm^{-2}. Non-*iR* correction is applied. The stable current density of ~213.7 mA cm^{-2} over 100 h of testing matches the performance on carbon cloth (215.5 mA cm^{-2}), validating the substrate-independent nature of the catalyst's durability. Source data are provided as a Source Data file.

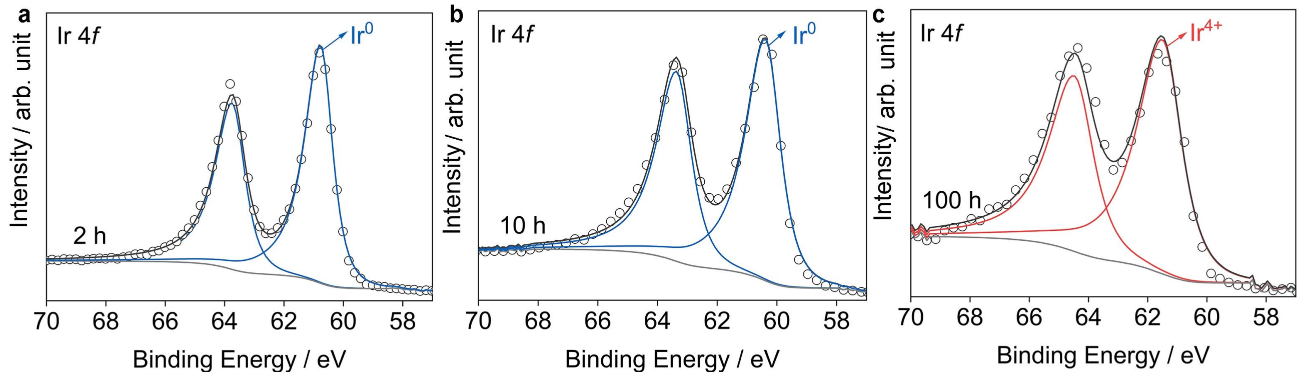

Fig. S26. Ir 4*f* XPS of the $Ag_{0.19}Ru_1Ir_{0.48}$ alloy catalysts sampled at different times in chronoamperometric testing at 1.74 V. (a) 2 h. **(b)** 10 h. **(c)** 100 h. Source data are provided as a Source Data file.

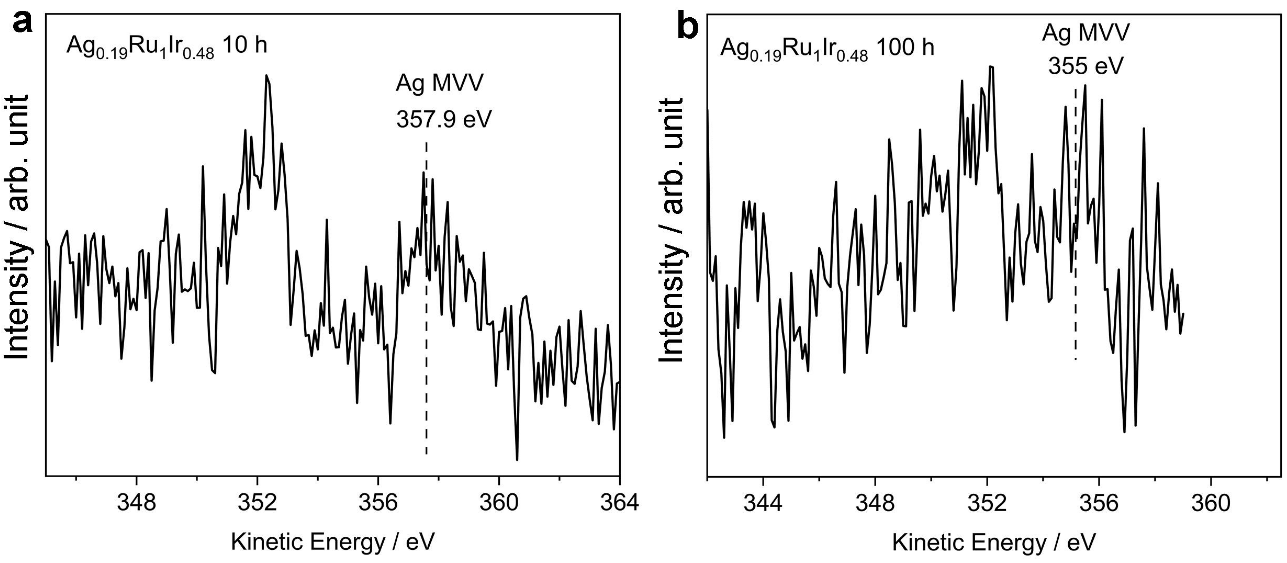

Fig. S27. Auger electron spectroscopy of Ag in $Ag_{0.19}Ru_1Ir_{0.48}$ alloy nanocages sampled at different times in chronoamperometric testing at 1.74 V. (a) 10 h. **(b)** 100 h. The Ag MVV peaks are observed at kinetic energies of 355–358 eV, corresponding to Ag^0. Source data are provided as a Source Data file.

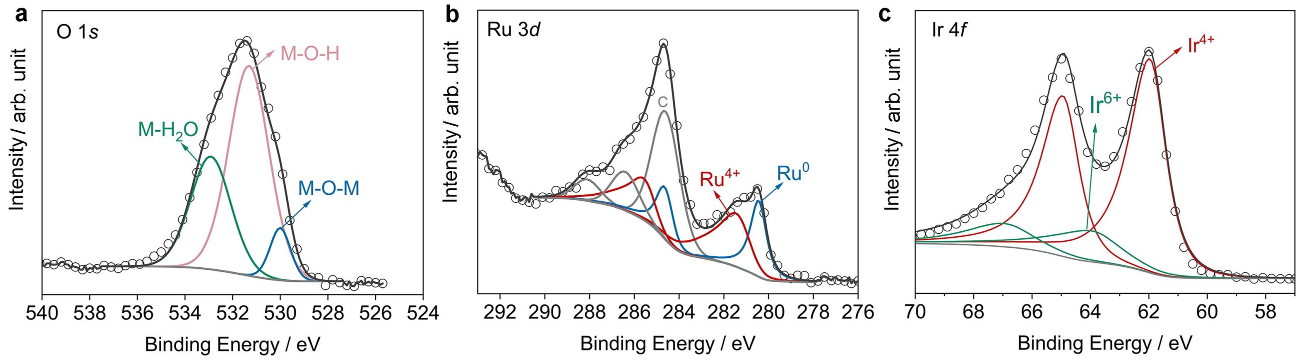

Fig. S28. O 1*s*, Ru 3*d*, and Ir 4*f* XPS of the $Ag_{0.19}Ru_1Ir_{0.48}$ catalyst after chronopotentiometric testing at 200 mA cm^{-2} for 1000 h. (a) O 1*s*. **(b)** Ru 3*d*. **(c)** Ir 4*f*. Source data are provided as a Source Data file.

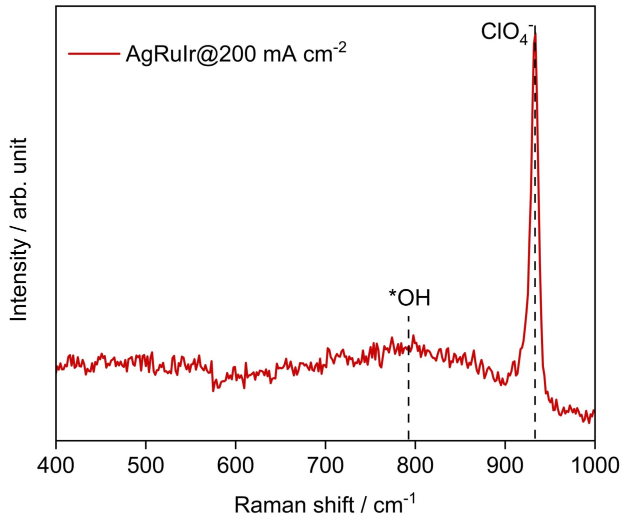

Fig. S29. Raman spectrum of the $Ag_{0.19}Ru_1Ir_{0.47}$ alloy nanocages after chronopotentiometric testing at 200 mA cm^{-2} for 100 h. Source data are provided as a Source Data file.

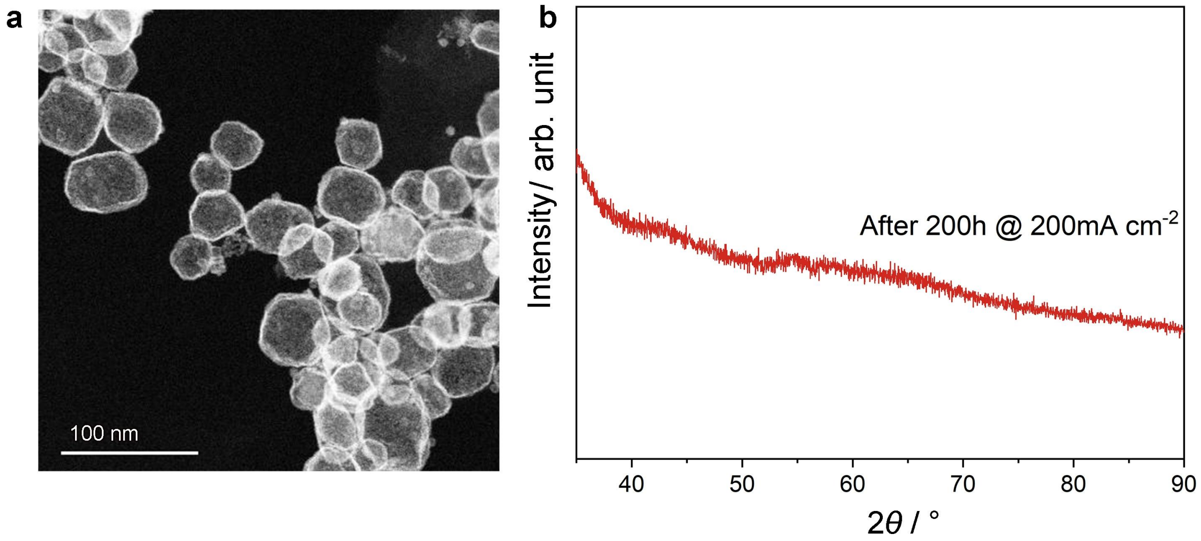

Fig. S30. Characterization of the $Ag_{0.19}Ru_1Ir_{0.48}$ catalyst after chronopotentiometric testing at 200 mA cm^{-2} for 200 h. (a) HAADF-STEM image. **(b)** XRD patterns. Source data are provided as a Source Data file.

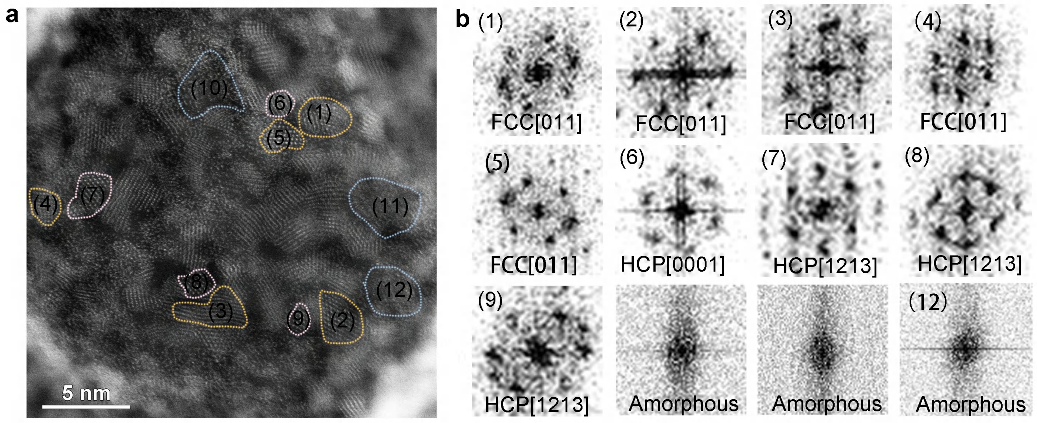

Fig. S31. Phase analysis of crystallites in the $Ag_{0.19}Ru_1Ir_{0.48}$ alloy catalyst after chronopotentiometric testing at 200 mA cm^{-2} for 200 h. (a) HADDF-STEM images of the nanocages. **(b)** HAADF-STEM and Fourier diffraction patterns of FCC and HCP, and amorphous crystallites, as labeled in (a).

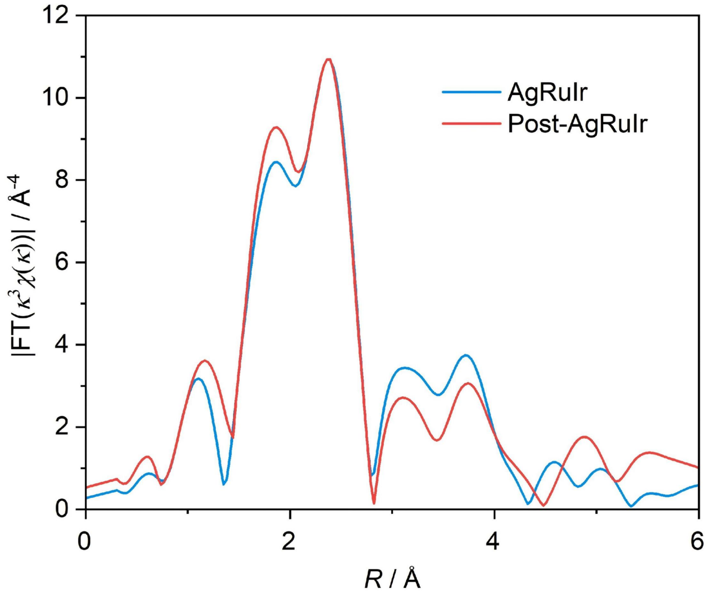

Fig. S32. Ru FT-EXAFS of the $Ag_{0.19}Ru_1Ir_{0.48}$ alloy nanocages before and after OER catalysis. *R*, radial distance. As a bulk-sensitive technique, X-ray absorption spectroscopy confirms the retention of the bulk metallic state in the $Ag_{0.19}Ru_1Ir_{0.48}$ alloy nanocages during the OER process. Source data are provided as a Source Data file.

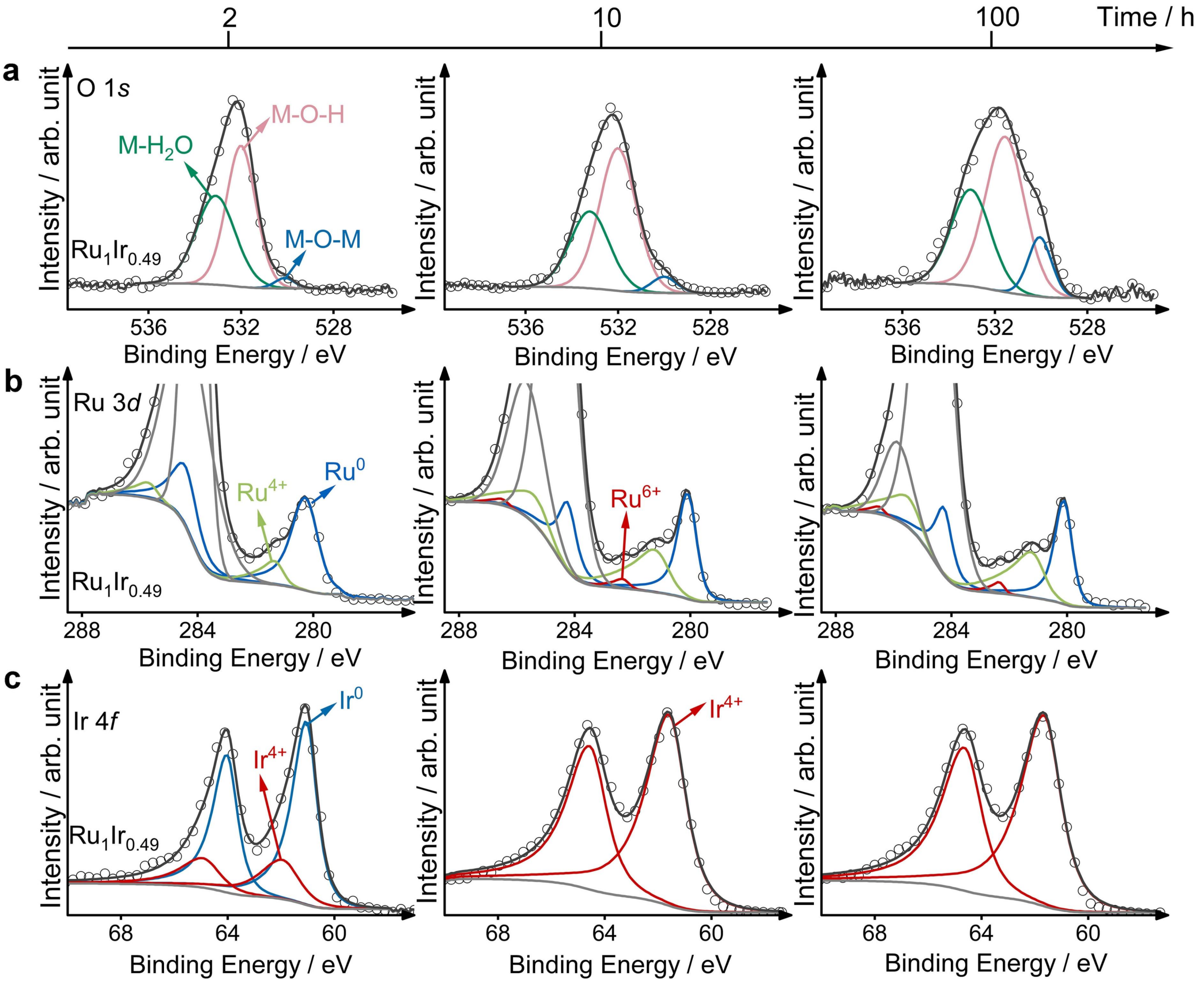

Fig. S33. XPS spectra of the $Ru_1Ir_{0.49}$ catalyst after chronoamperometric testing at 1.6 V for 2, 10, and 100 h. (a) O 1*s*. **(b)** Ru 3*d*. **(c)** Ir 4*f*. Note that when the chronoamperometric testing was carried out at an elevated potential of 1.74 V, catalysts rapidly degraded within 3 h, and no XPS signals were detectable. Source data are provided as a Source Data file.

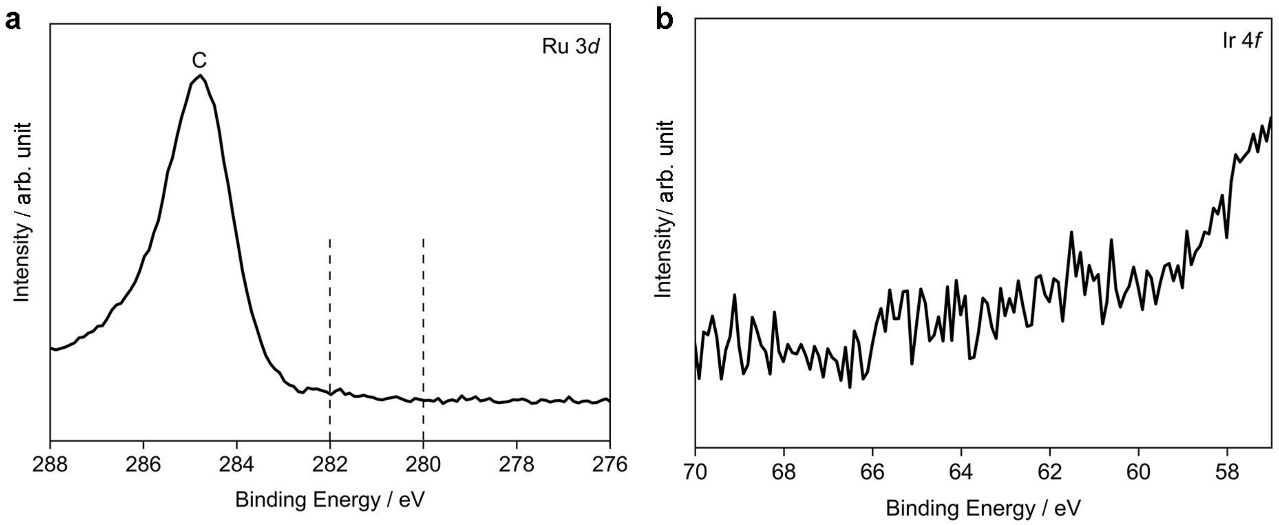

Fig. S34. XPS spectra of the $Ru_1Ir_{0.49}$ catalyst after chronoamperometric testing at 1.74 V for 3 h. (**a**) Ru 3*d*. The dotted lines indicate the expected positions for Ru 3*d* peaks. (**b**) Ir 4f. At this elevated potential, the Ag-free RuIr alloy catalysts underwent rapid degradation within 3 h, resulting in the absence of detectable XPS signals. Source data are provided as a Source Data file.

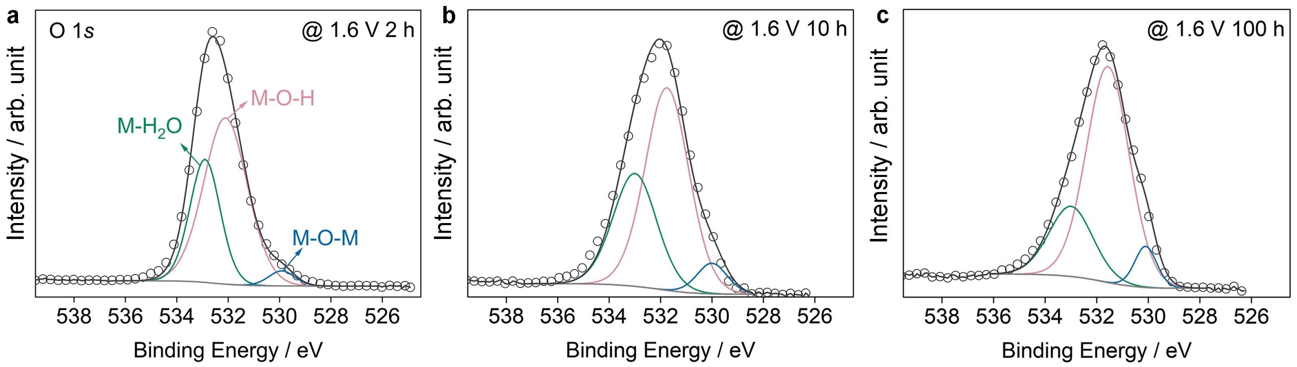

Fig. S35. O 1*s* XPS spectra of the Ag-deficient $Ag_{0.06}Ru_1Ir_{0.47}$ catalyst sampled at different times of chronoamperometric testing at 1.6 V. (a) At 2 h. **(b)** At 10 h. **(c)** At 100 h. Source data are provided as a Source Data file.

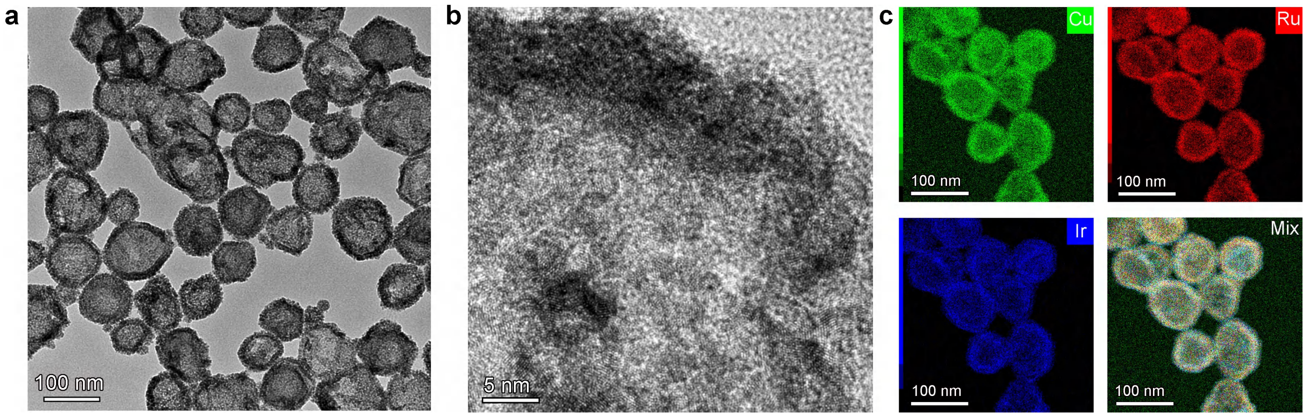

Fig. S36. Characterization of the $Cu_{0.16}Ru_1Ir_{0.47}$ alloy nanocages. (a) Low-magnification TEM image, showing the nanocage morphology. **(b)** HRTEM image, showing that the nanocages are polycrystalline composed of numerous crystallites. **(c)** EDS elemental mapping, confirming the efficient Cu–Ru–Ir alloying within he nanocages.

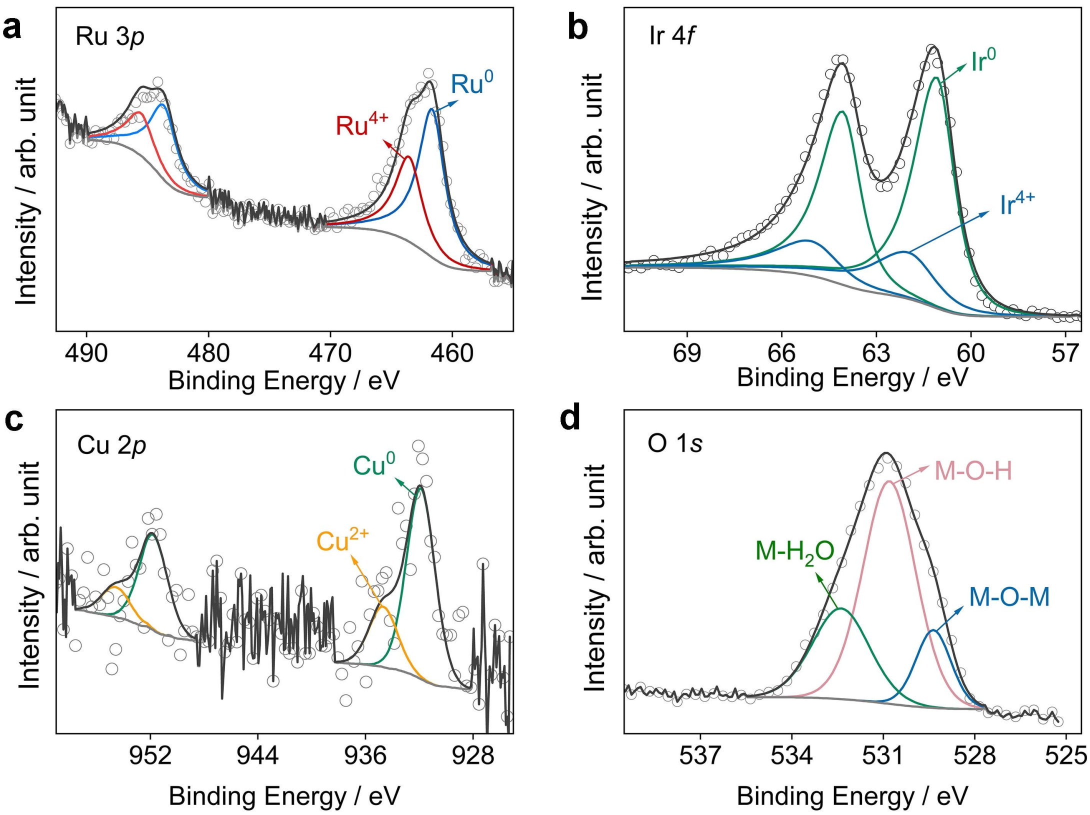

Fig. S37. XPS spectra of the $Cu_{0.16}Ru_1Ir_{0.47}$ alloy nanocages. (a) Ru 3*p*. **(b)** Ir 4*f*. **(c)** Cu 2*p*. **(d)** O 1*s*. The spectra were acquired after chronoamperometric testing at 1.74 V for 2 h. Source data are provided as a Source Data file.

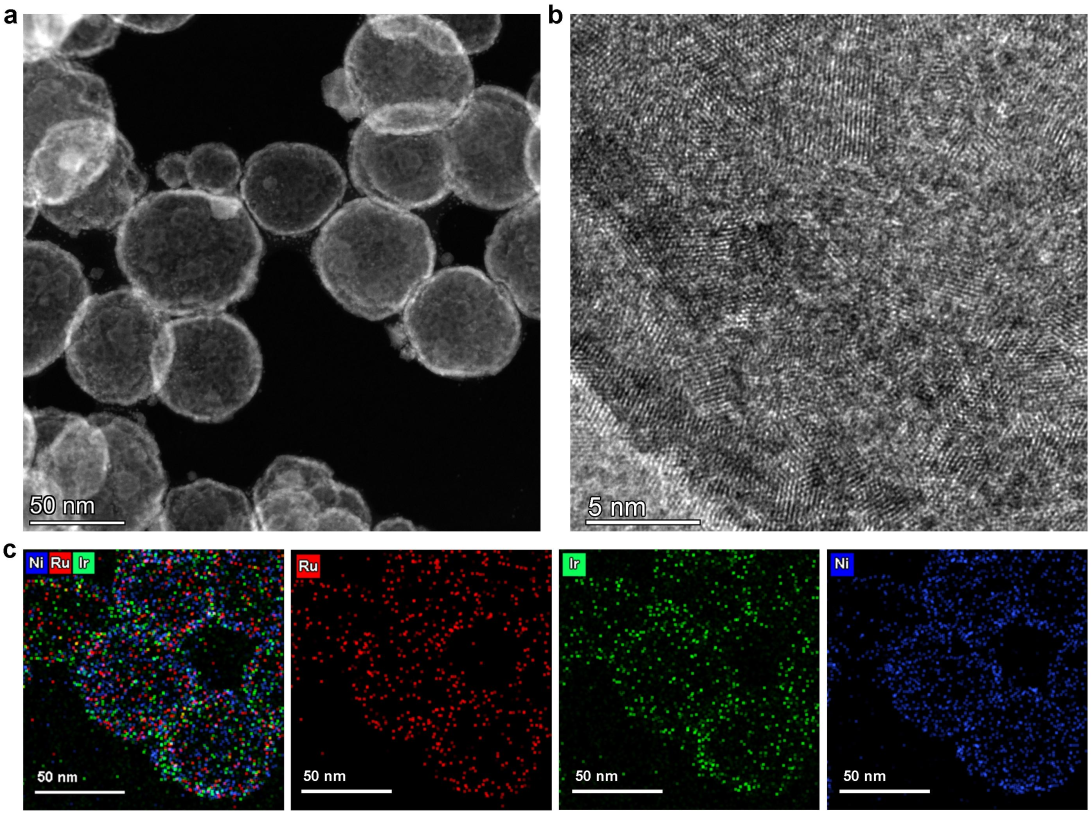

Fig. S38. Characterization of the $Ni_{0.22}Ru_1Ir_{0.48}$ alloy nanocages. (a) Low-magnification TEM image, showing the nanocage morphology. **(b)** HRTEM image, showing that the nanocages are polycrystalline composed of numerous crystallites. **(c)** EDS elemental mapping, confirming the efficient Ni–Ru–Ir alloying within he nanocages.

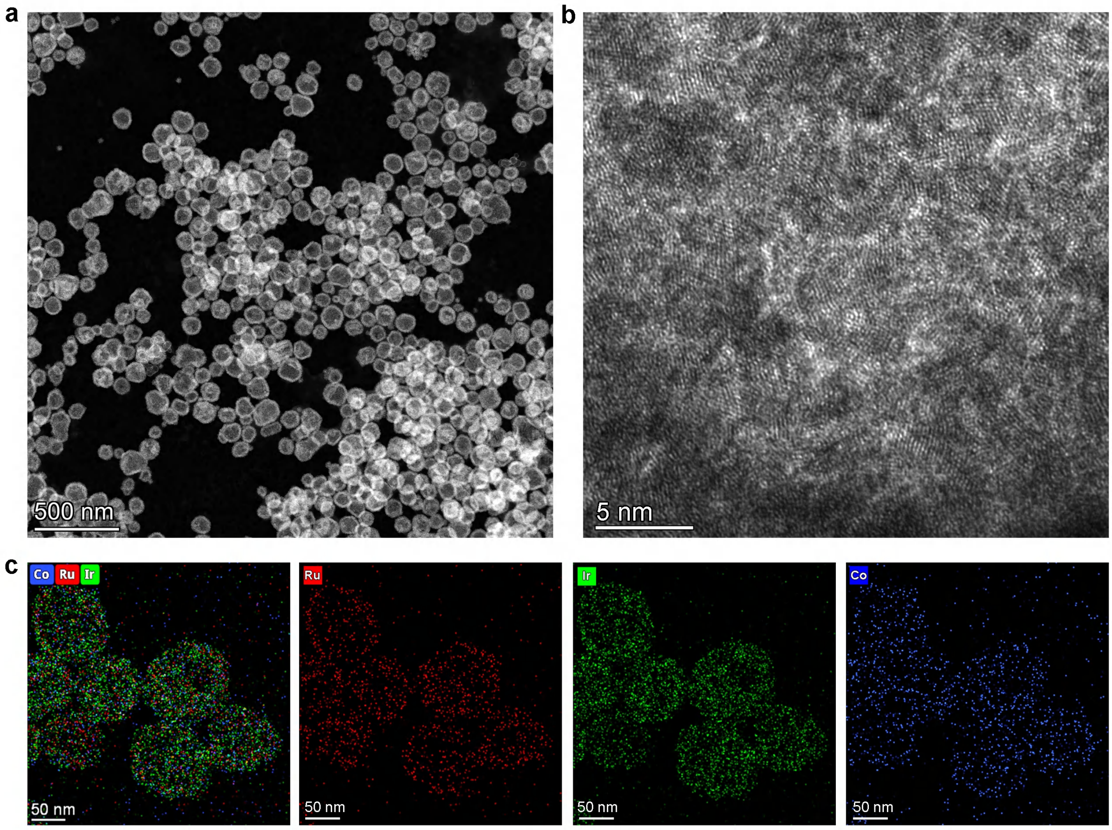

Fig. S39. Characterization of the $Co_{0.25}Ru_1Ir_{0.46}$ alloy nanocages. (a) Low-magnification TEM image, showing the nanocage morphology. **(b)** HRTEM image, showing that the nanocages are polycrystalline composed of numerous crystallites. **(c)** EDS elemental mapping, confirming the efficient Co–Ru–Ir alloying within he nanocages.

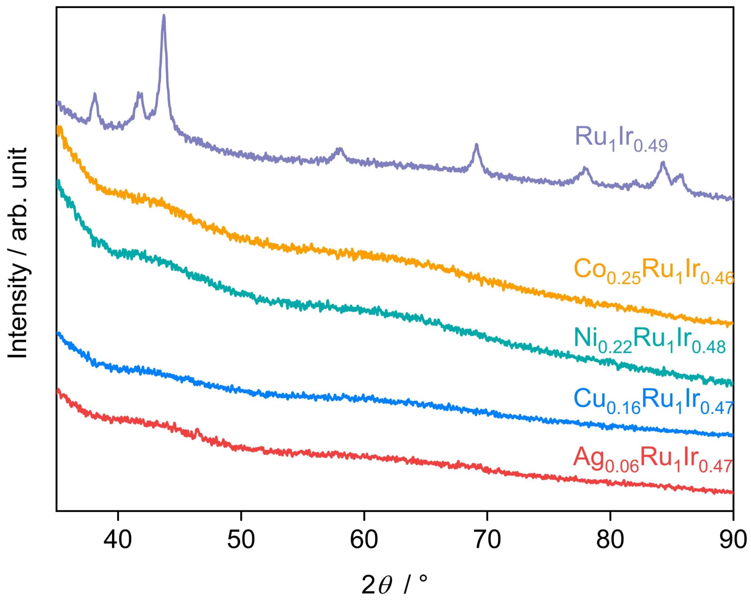

Fig. S40. XRD patterns of the $Cu_{0.16}Ru_1Ir_{0.47}$, $Ni_{0.22}Ru_1Ir_{0.48}$, $Co_{0.25}Ru_1Ir_{0.46}$, $Ag_{0.06}Ru_1Ir_{0.47}$ and $Ru_1Ir_{0.49}$ catalysts. The $Ru_1Ir_{0.49}$ catalyst synthesized by impregnation methods was highly crystalline. The other catalysts synthesized by etching-induced vacancy diffusion mechanism showed no clear XRD peaks, indicating low crystallinity, agreeing with their polycrystalline nature and ultrasmall crystallite sites. Source data are provided as a Source Data file.

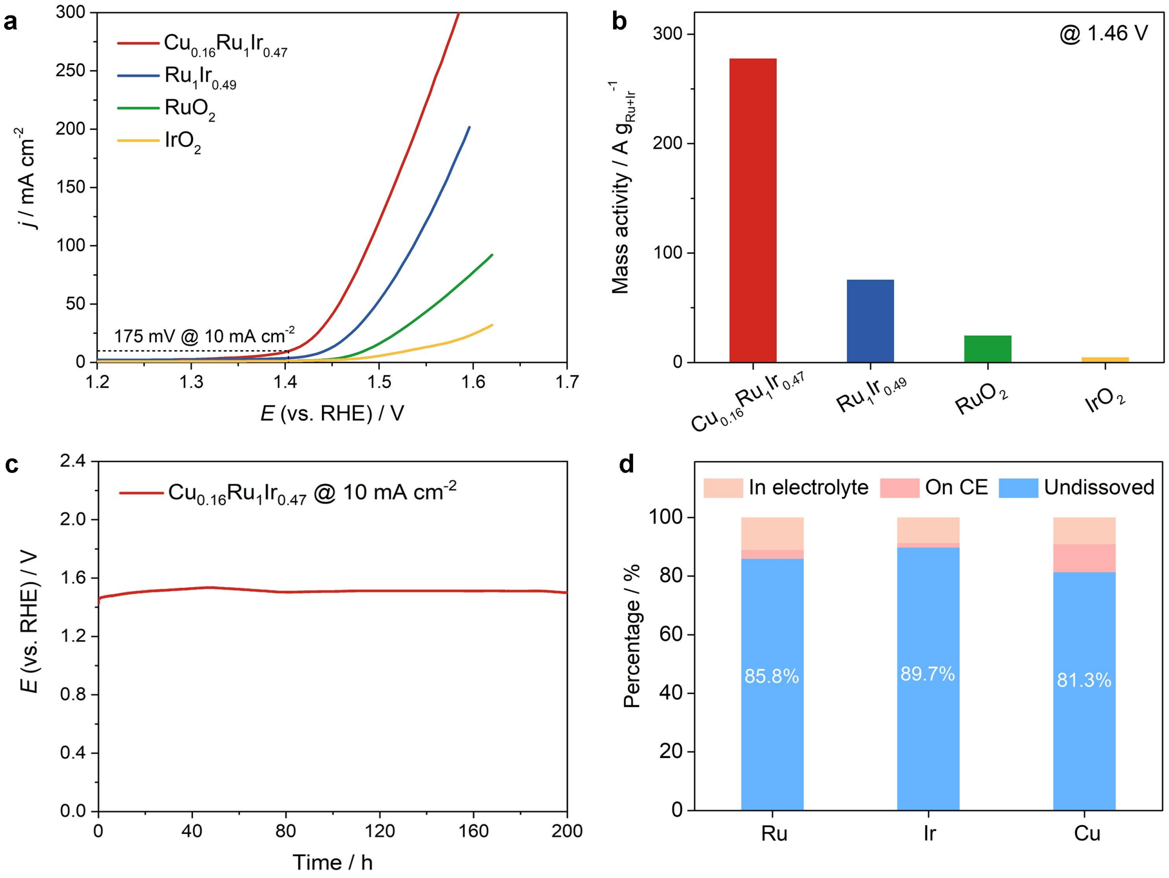

Fig. S41. Electrochemical OER activity and stability of the $Cu_{0.16}Ru_1Ir_{0.47}$ alloy catalyst. (a) LSV curves in O_2-saturated 0.1 M $HClO_4$ (pH = 1 ± 0.01) at 25 °C, scan rate: 10 mV s^{-1}. *iR* compensation level: 95%. Catalyst loading: 0.134 mg cm^{-2}. (b) Mass activities at 1.46 V. Catalysts for comparison: $Ru_1Ir_{0.49}$, and commercial RuO_2 and IrO_2. **(c)** Chronopotentiometric curve at 10 mA cm^{-2} in 0.1 M $HClO_4$ (pH = 1 ± 0.01) at 25 °C. Non-*iR* correction is applied. **(d)** Ratios of dissolved Ru, Ir, and Cu after chronopotentiometric testing at 10 mA cm^{-2} for 200 h. Source data are provided as a Source Data file.

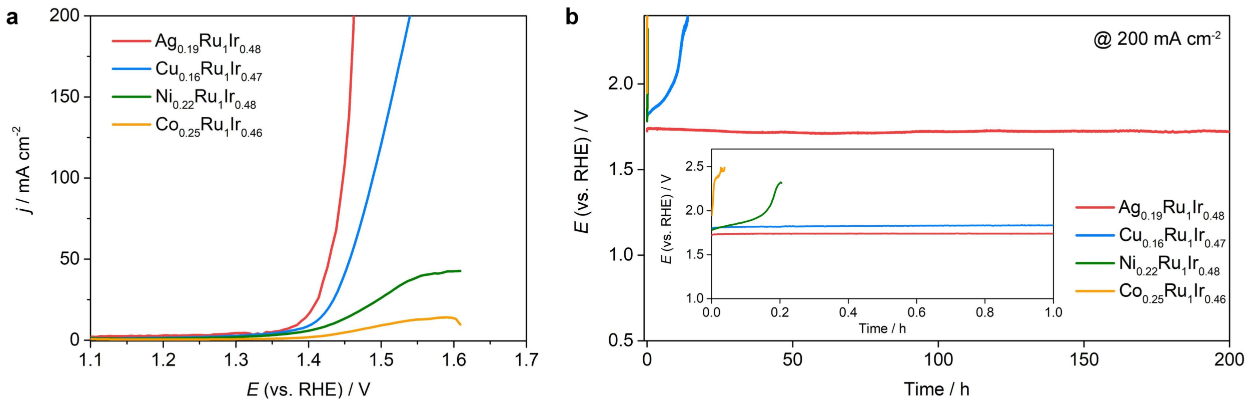

Fig. S42. Electrochemical OER performance of the MRuIr (M = Ag, Cu, Ni, and Co) alloy catalysts. (a) LSV curves in O_2-saturated 0.1 M $HClO_4$ (pH = 1 ± 0.01) at 25 °C, scan rate: 10 mV s^{-1}. *iR* compensation level: 95%. Catalyst loading: 0.134 mg cm^{-2}. **(b)** Chronopotentiometric curves at 200 mA cm^{-2} in 0.1 M $HClO_4$ (pH = 1 ± 0.01) at 25 °C. Non-*iR* correction is applied. Inset: The chronopotentiometric curves within the first 1 h. Source data are provided as a Source Data file.

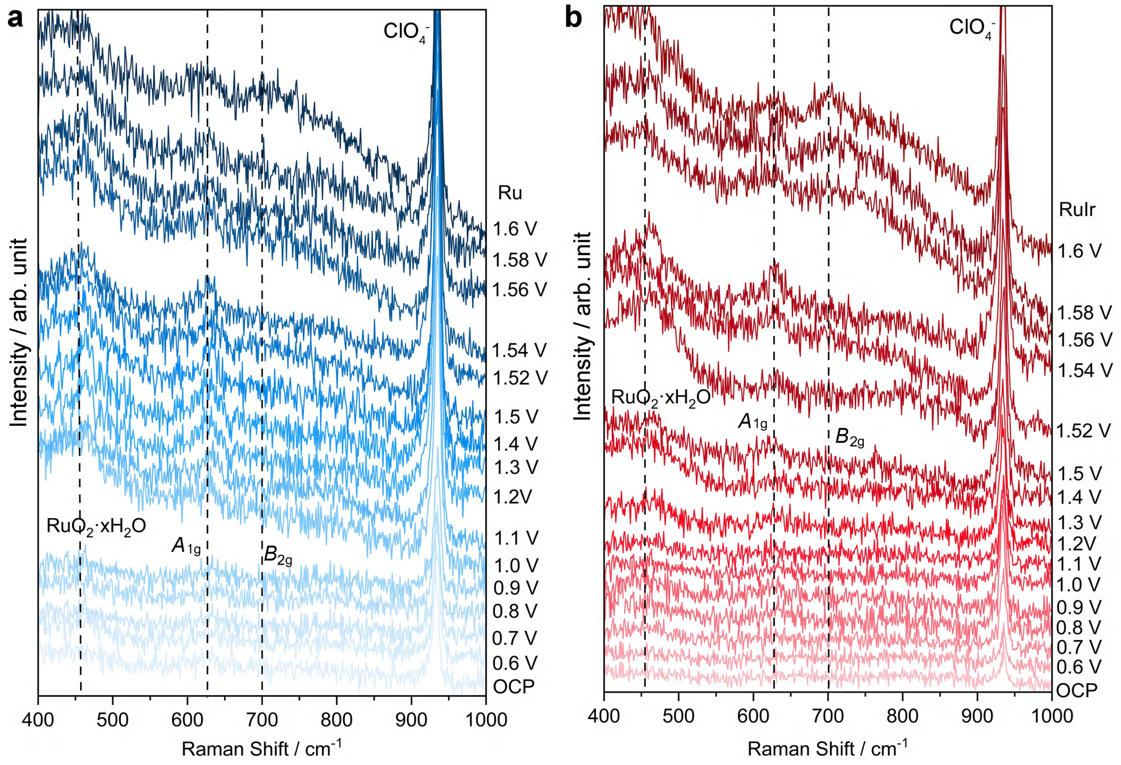

Fig. S43. Validating the role of Ir in elevating the reduction potentials of metals in the alloy system. (**a**) Potential-dependent in situ Raman spectra of Ru/C in O_2-saturated 0.1 M $HClO_4$ from OCP to 1.6 V. (**b**) Corresponding in situ Raman spectra for RuIr/C under identical conditions. The dashed lines indicate the positions of characteristic vibrational modes for rutile-type oxides and amorphous hydrated RuO_2 ($RuO_2 \cdot H_2O$). The delayed emergence of these features in RuIr compared to pure Ru demonstrates that Ir elevates the oxidation potential of the alloy, providing direct evidence for enhanced oxidation resistance. Source data are provided as a Source Data file.

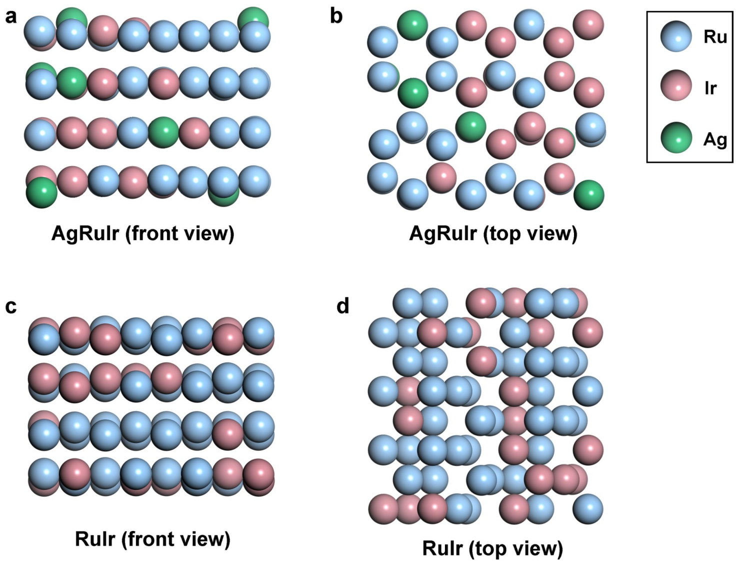

Fig. S44. Atomic models of AgRuIr and RuIr for DFT calculations. (a, b) AgRuIr. **(c, d)** RuIr.

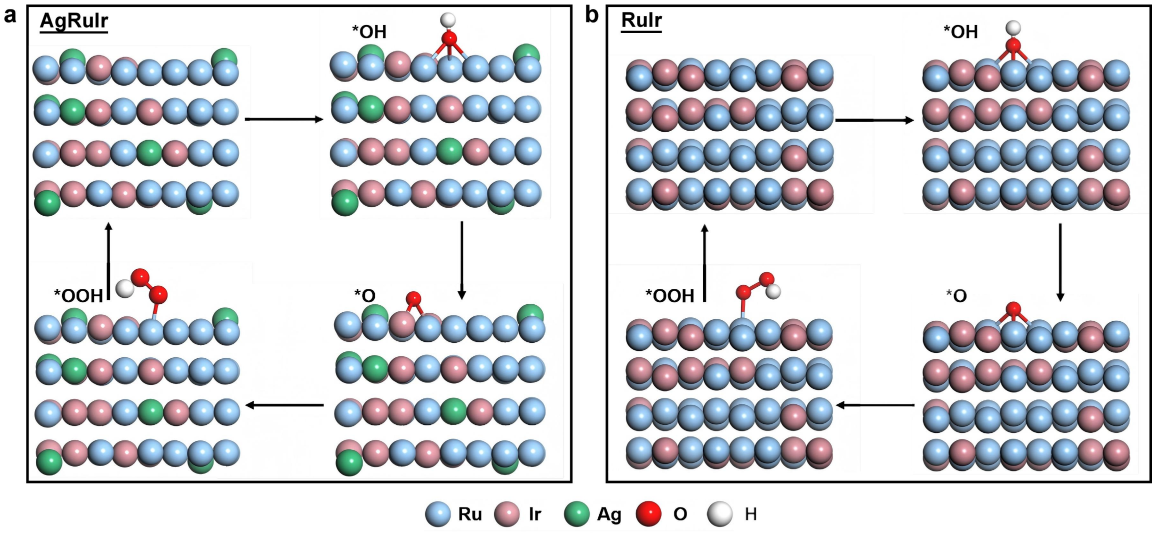

Fig. S45. Calculated configurations of OER intermediates adsorbed on the AgRuIr and RuIr surfaces. (a) On AgRuIr. **(b)** On RuIr.

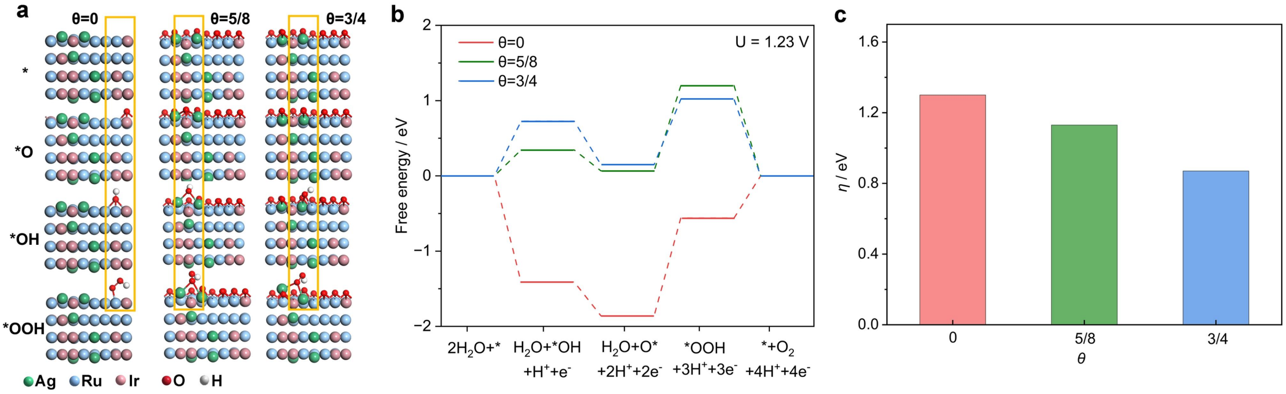

Fig. S46. Effect of surface oxygen coverage (*θ*) on AgRuIr alloy surface on OER kinetics. (**a**) Adsorption configurations of oxygenated OER intermediates on alloy surfaces at different surface oxygen coverages (*θ* = 0, 5/8, and 3/4). (**b**) Gibbs free energy profiles of the OER on AgRuIr with varying surface oxygen coverages. (**c**) Energy increase of the potential-determining step as a function of surface oxygen coverage. Source data are provided as a Source Data file.

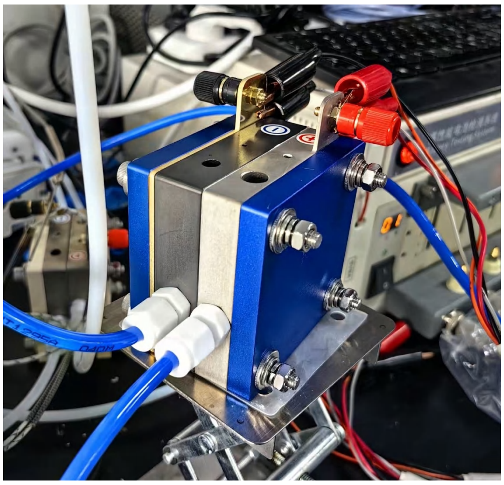

Fig. S47. PEMWE device. The device was assembled for evaluating the performance of the catalysts in PEM water electrolysis.

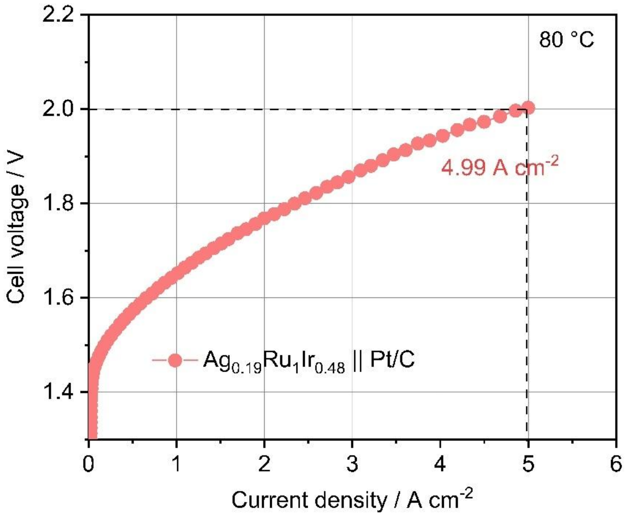

Fig. S48. PEMWE performance at 80 °C. Polarization curve using $Ag_{0.19}Ru_{1}Ir_{0.48}$ as the anode catalyst and commercial Pt/C as the cathode catalyst. Testing was performed at 80 °C without *iR* compensation. Source data are provided as a Source Data file.

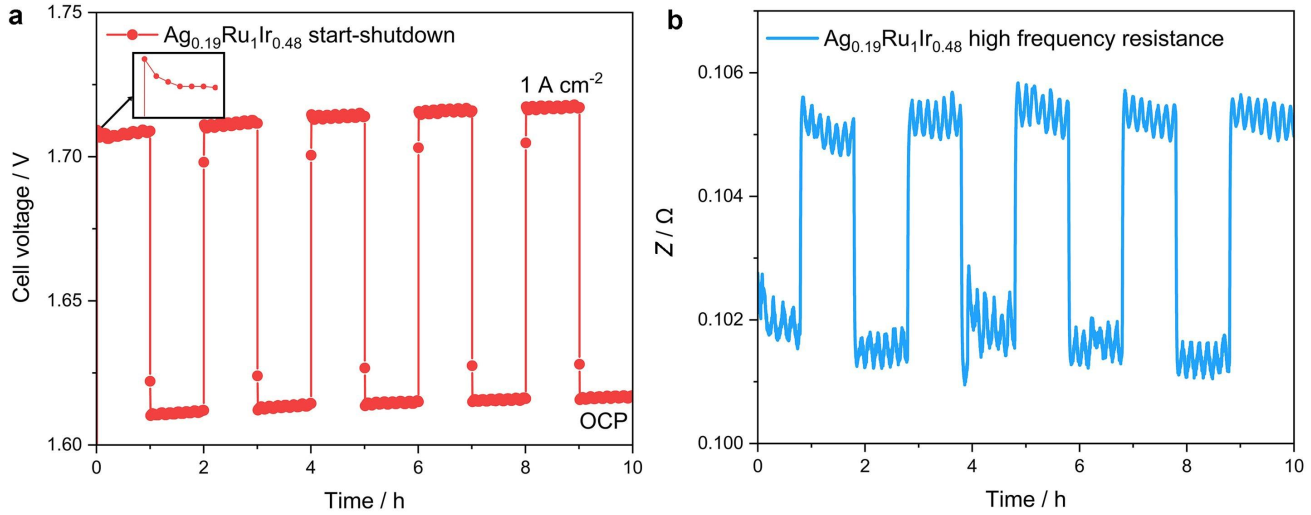

Fig. S49. Dynamic start-stop cycling performance of $Ag_{0.19}Ru_1Ir_{0.48}$ alloy nanocages in a PEMWE single cell. (**a**) Cell voltage profile during intermittent start-stop cycles, consisting of alternating periods of 1 h operation at 1 A cm^{-2} and 1 h at open-circuit potential (OCP). Inset: A zoom-in view of the cell-voltage profile during the transition from OCP to the start of the next cycle, demonstrating the rapid stabilization of the potential without significant voltage transients or "jumps." (**b**) Real-time monitoring of high-frequency resistance (HFR) during the cycling test, confirming the robust structural and electrical integrity of the MEA under dynamic operating conditions. Non-*iR* correction is applied. Source data are provided as a Source Data file.

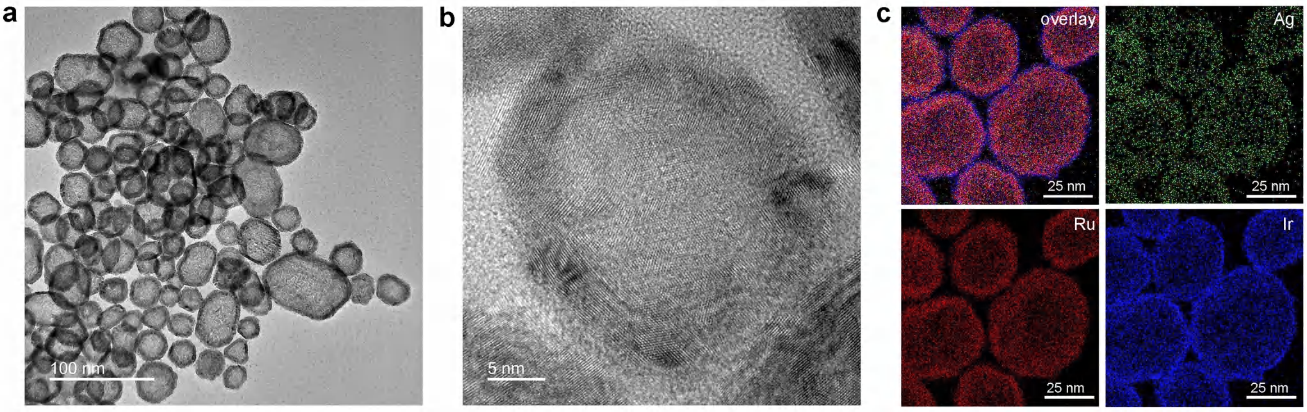

Fig. S50. Structural and compositional characterization of $Ag_{0.19}Ru_1Ir_{0.48}$ alloy nanocages after the start-stop testing. (**a**) TEM image showing the preservation of the hollow nanocage morphology. (**b**) HRTEM image resolving metallic lattice fringes, confirming the stability of the alloy framework. (**c**) EDS mapping showing the homogeneous distribution of Ag, Ru, and Ir after dynamic operation.

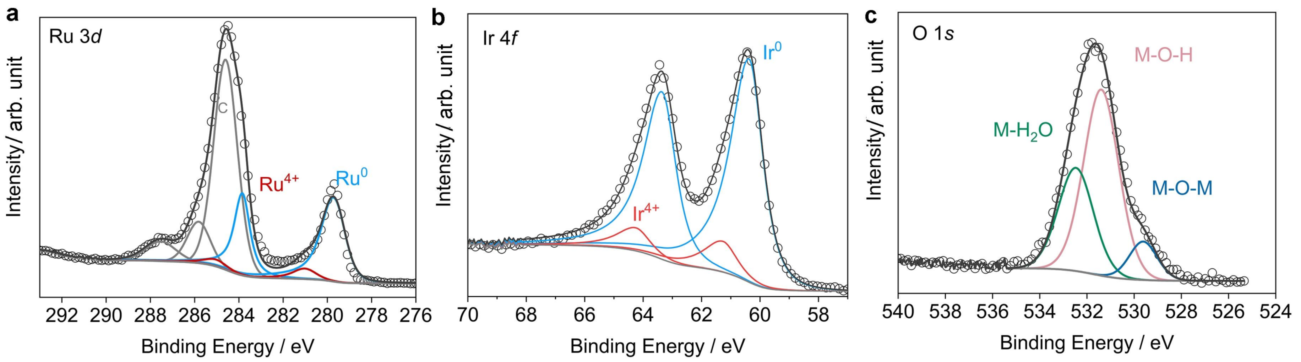

Fig. S51. XPS analysis of the $Ag_{0.19}Ru_1Ir_{0.48}$ alloy nanocages harvested from the MEA after the start-stop testing. (**a**) Ru 3*d*. (**b**) Ir 4*f*. (**c**) O 1*s*. The spectra confirm the dominant metallic state and suppressed oxidation of the catalyst under non-steady-state conditions. Source data are provided as a Source Data file.

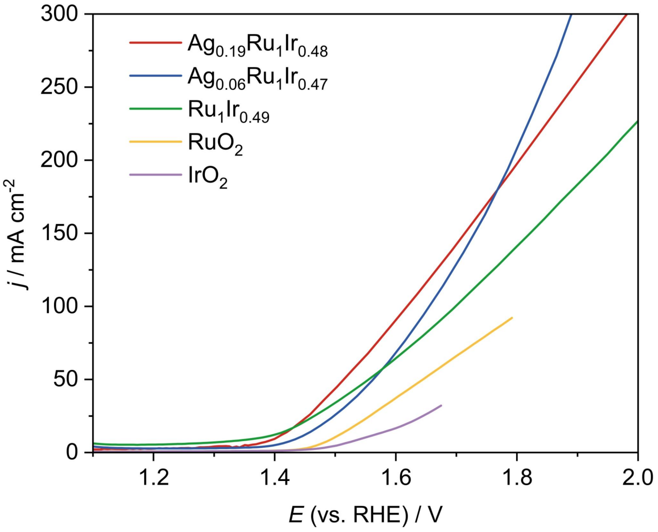

Fig. S52. Non-*iR* compensated LSV curves of catalysts in half-cell (pH = 1) at 25 °C. Scan rate, 10 mV s^{-1}. Source data are provided as a Source Data file.

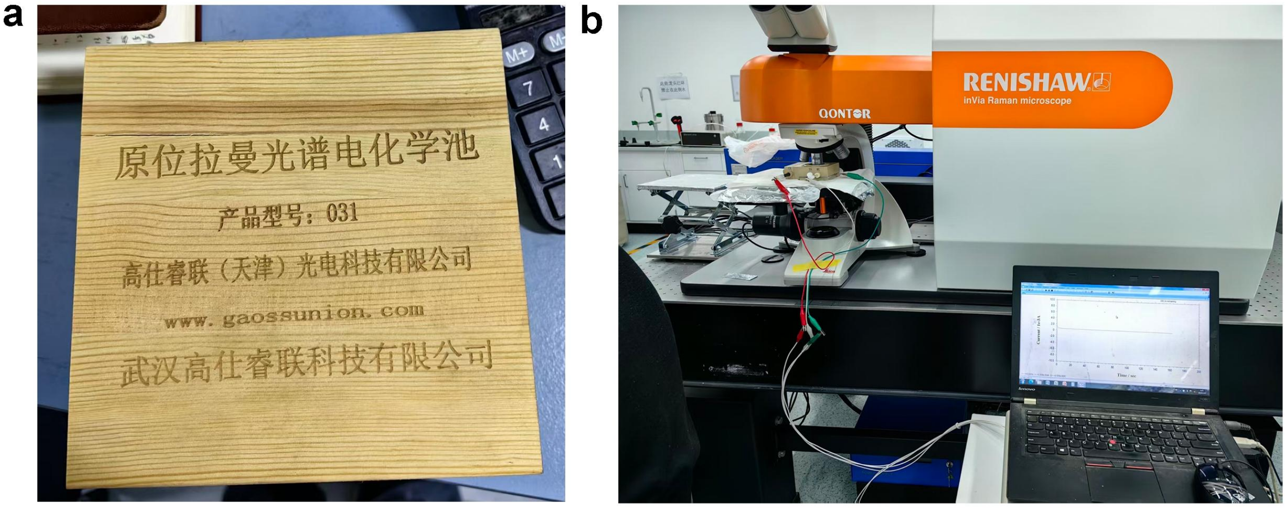

Fig. S53. Setup for in situ Raman testing. (a) Model of Customized in situ Raman Spectroelectrochemical Cell. **(b)** Optical photograph of in situ Raman testing system.

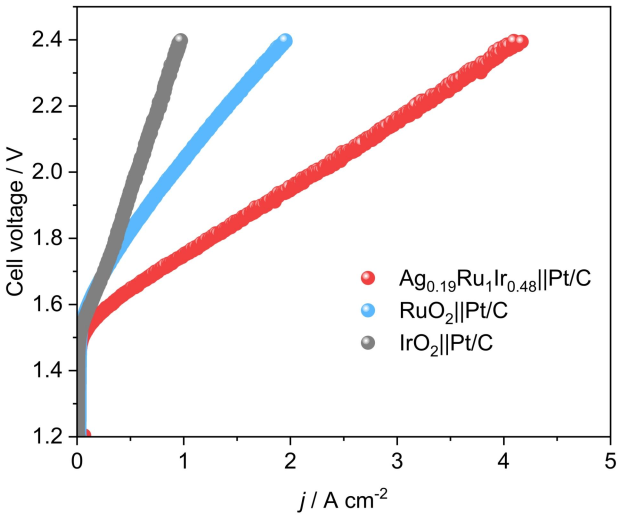

Fig. S54. Non-*iR* compensated polarization curves of PEMWE in pure water at 60 °C using $Ag_{0.19}Ru_1Ir_{0.48}$, RuO_2, and IrO_2 as the anode catalyst and commercial Pt/C as the cathode catalyst. Scan rate, 10 mV s^{-1}. Source data are provided as a Source Data file.

Table S1. ICP-MS of the $Ag_{0.19}Ru_1Ir_{0.48}$ alloy catalyst.

	Ru / Atomic%	Ir / Atomic%	Ag / Atomic%
$Ag_{0.19}Ru_1Ir_{0.48}$	60	29	11

Table S2. The acidic OER performance of $Ag_{0.19}Ru_1Ir_{0.48}$ compared with those of recently reported catalysts.

Catalysts	Electrolyte	$\eta^{\#}$@10 mA cm^{-2} (25 °C)	Stability	Ref
**$Ag_{0.19}Ru_1Ir_{0.48}$**	**0.1 M $HClO_4$ (pH = 1 ± 0.01)**	**160 mV (95% *iR*, loading, 0.134 mg cm^{-2})**	**1500 h*** (Nafion 117, loading, 0.934 mg cm^{-2})**@1 A cm^{-2}**	**This work**
12Ru/MnO_2	0.1 M $HClO_4$	161 mV (loading, 0.2 mg cm^{-2})	200 $h^{\#}$@10 mA cm^{-2}	1
WErRuO	0.5 M H_2SO_4	170 mV (loading, 0.33 mg cm^{-2})	100 $h^{\#}$@10 mA cm^{-2}	2
Zn-doped RuO_2	0.5 M H_2SO_4 (pH = 0.30 ± 0.01)	173 mV (85% *iR*, loading, 0.6 mg cm^{-2})	1000 $h^{\#}$@10 mA cm^{-2}	3
M-RuIrFeCoNiO_2	0.5 M H_2SO_4	189 mV (95% *iR*, loading, 0.476 mg cm^{-2})	500 h* (Nafion 212, loading, 2 mg cm^{-2})@1 A cm^{-2}	4
CdRuIrO	0.5 M H_2SO_4	189 mV (95% *iR*, loading, 1 mg cm^{-2})	1500 $h^{\#}$@10 mA cm^{-2}	5
SrRuIrO	0.5 M H_2SO_4 (pH = 0.30)	190 mV (95% *iR*, loading, 0.32 mg cm^{-2})	150 h* (loading, 1.5 mg cm^{-2})@1 A cm^{-2}	6
SnRuOx	0.5 M H_2SO_4	194 mV (41.65 μg cm^{-2})	1300 h*(Nafion 212, loading, 4 mg cm^{-2})@1 A cm^{-2}	7
$Ta_{0.1}Tm_{0.1}Ir_{0.8}O_{2-\delta}$	0.5 M H_2SO_4	198 mV (40.8 μg cm^{-2})	500 $h^{\#}$@10 mA cm^{-2}	8
$Nb_{0.1}Ru_{0.9}O_2$	0.5 M H_2SO_4	204 mV (0.25 mg cm^{-2})	511 $h^{\#}$@10 mA cm^{-2}	9
Ni-RuO_2	0.1 M $HClO_4$ (pH = 1)	214 mV (100% *iR*, loading, 0.4 mg cm^{-2})	1000 h* (Nafion 117, loading, 3.1 mg cm^{-2})@200 mA cm^{-2}	10
$RuWO_x$	0.5 M H_2SO_4 (pH = 0.2)	230 mV (95% *iR*, loading, 0.11 mg cm^{-2})	550 $h^{\#}$@10 mA cm^{-2}	11

RuMn	0.5 M H_2SO_4	239 mV (95% *iR*, loading, 0.1 mg cm^{-2})	140 h*(Nafion 117)@10 mA cm^{-2}	12
N-RuO_2/TiN	0.5 M H_2SO_4	159 mV (85% *iR*, loading, 0.28 mg cm^{-2})	1100 h* (Nafion 212, loading, 0.5 mg cm^{-2})@1A cm^{-2}	13
Ru/MnO_2	0.5 M H_2SO_4	179 mV	500 h* (Nafion 115, 0.1 mg_{Ru} cm^{-2})@500 mA cm^{-2}	14
$iGa_{0.2}Ru_{0.8}O_2$	1 M $HClO_4$ (pH = 0.08 ± 0.01)	188 mV (100% *iR*, loading, 0.2 mg cm^{-2})	200 h*(Hyproof HPM2080X)@500 mA cm^{-2}	15
GB-V-RuO_2	0.5 M H_2SO_4 (pH =0.3 ± 0.01)	159 mV (95% *iR*, loading, 0.5 mg cm^{-2})	500 h*(loading, 1 mg cm^{-2})@200 mA cm^{-2}	16
Ru-RuO_2	0.5 M H_2SO_4 (pH = 0.3)	165 mV	1100 h* (Nafion 115)@100 mA cm^{-2}	17
$Mn_{(SA)}$/RuO_2	0.5 M H_2SO_4 (pH = 0)	213 mV (loading, 0.52 mg cm^{-2})	1000 h# (loading, 3.2 mg cm^{-2})@10 mA cm^{-2}	18
$W_{60}Ir_{20}B_{20}$	0.5 M H_2SO_4	291 mV (loading, 0.25 mg cm^{-2})	800 h#@100 mA cm^{-2}	19

# via half-cell testing.

* via PEM testing.

Table S3. The overpotential of $Ag_{0.19}Ru_1Ir_{0.48}$ at high current densities (100 and 200 mA cm^{-2}) was determined by LSV via half-cell testing, compared with those of recently reported catalysts.

Catalysts	η@100 mA cm^{-2}	η@200 mA cm^{-2}	Ref.
**$Ag_{0.19}Ru_1Ir_{0.48}$**	**215 mV (95% *iR*, loading, 0.134 mg cm^{-2})**	**233 mV (95% *iR*, loading, 0.134 mg cm^{-2})**	**This work**
WErRuO	221 mV (loading, 0.33 mg cm^{-2})	253 mV (loading, 0.33 mg cm^{-2})	2
CaCuRuO	221 mV (loading, 0.25 mg cm^{-2})	250 mV (loading, 0.25 mg cm^{-2})	20
V_o-RuO_2	230 mV (loading, 0.25 mg cm^{-2})	–	21
Zn-doped RuO_2	245 mV (85% *iR*, loading, 0.6 mg cm^{-2})	–	3
$Bi_{1.5}Er_{0.5}Ru_2O_7$	249 mV	–	22
$CdRuIrO_x$	250 mV (95% *iR*, loading, 1 mg cm^{-2})	–	5
Ni-RuO_2	260 mV (100% *iR*, loading, 0.4 mg cm^{-2})	–	10
Ir-Sn PSC	264 mV (loading, 0.2 mg cm^{-2})	–	23
$La_{0.52}RuO_2$	265 mV (loading, 0.637 mg cm^{-2})	337 mV (loading, 0.637 mg cm^{-2})	24
MD-RuO_2-BN	266 mV (loading, 0.7 mg cm^{-2})	–	25
Ir-MnO_2	270 mV (loading, 1 mg cm^{-2})	300 mV (loading, 1 mg cm^{-2})	26
Pt-doped RuO_2	270 mV (95% *iR*, loading, 0.3 mg cm^{-2})	–	27
$ZnRuO_x$	285 mV (loading, 0.25 mg cm^{-2})	–	28
m-RuO_2	295 mV (90% *iR*, loading, 0.2 mg cm^{-2})	420 mV (90% *iR*, loading, 0.2 mg cm^{-2})	29
Ni@$Ir_{0.44}Ru_{0.56}$	335 mV	421 mV	30
$RuSnO_x$	350 mV (loading, 0.2 mg cm^{-2})	–	31

Table S4. Equivalent circuit and fitting parameters for the Nyquist EIS plots of the $Ag_{0.19}Ru_1Ir_{0.48}$, $Ag_{0.06}Ru_1Ir_{0.47}$, $Ru_1Ir_{0.49}$, RuO_2 and IrO_2 catalysts in electrocatalytic OER (Corresponding to Fig. 3e).

Model:

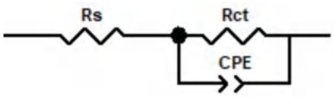

Catalysts	R_s/Ω	CPE/mF	R_{ct}/Ω
$Ag_{0.19}Ru_1Ir_{0.48}$	6.8	21.8	3.7
$Ag_{0.06}Ru_1Ir_{0.47}$	7.1	16.1	8.8
$Ru_1Ir_{0.49}$	7.1	6.1	78.2
RuO_2	7.2	5.7	132.4
IrO_2	7.5	1.7	321.7

Note: The Nyquist plots in Fig. 3e are each characterized by a single semi-circular feature, suggesting a typical electrochemical double-layer structure. Consequently, a standard R_s (R_{ct} CPE) equivalent circuit model was employed to fit these data. In this model, R_s represents the series solution resistance (or ohmic resistance), R_{ct} is the charge-transfer resistance (inversely correlates with the OER activity), and CPE denotes the constant phase element (commonly used to model the non-ideal capacitive behavior of an electrochemical interface, often arising from surface roughness or the heterogeneous distribution of active sites on the catalyst surface).

Table S5. Dissolved metal ratios of the catalysts measured by ICP-MS after chronopotentiometric testing at 200 mA cm^{-2}.

Catalyst	*t*/h	Elements	Initial/μg	In electrolyte /μg	On CE /μg	Undissolved /%
$Ag_{0.19}Ru_1Ir_{0.48}$	1000	Ru	157.2	5.8	2.01	95.03
		Ir	147	3.8	2	96.05
$Ru_1Ir_{0.48}$	0.417	Ru	159.4	111.2	7.73	25.4
		Ir	149.3	93.5	5.3	33.9
RuO_2	0.2	Ru	300	169.25	49.6	27.1
IrO_2	0.333	Ir	300	141.7	5.6	50.9

Initial: Mass of metals deposited on carbon cloth.

In electrolyte: Mass of metals dissolved in the electrolyte.

On CE: Mass of metals redeposited on the counter electrode.

Undissolved: Undissolved metal ratios, calculated by mass balance:

Undissolved = (Initial – In electrolyte – On CE)/Initial × 100%.

Table S6. Dissolved metal ratios of the catalysts measured by ICP-MS after chronoamperometric testing at 1.74 V.

Catalysts	t/h	Elements	Initial /μg	On WE /μg	In electrolyte /μg	On CE /μg	Peeled from WE /μg	Undissolved /%
$Ag_{0.19}Ru_1Ir_{0.48}$	100	Ru	62.9	54.9	2	0.78	5.22	95.7
		Ir	58.8	52.1	1.7	0.66	4.34	96.0
		Ag	12.8	10.5	0.61	0.1	1.59	96.0
$Ag_{0.06}Ru_1Ir_{0.47}$	100	Ru	68.8	25.3	34.1	1.36	8.04	48.5
		Ir	60.3	26.2	27.8	0.87	5.43	52.3
		Ag	4.76	1.16	2.5	0.12	0.98	45.9
$Au_{0.2}Ru_1$	3	Ru	80.5	35.1	41	0.55	3.85	48.4
		Ag	15.9	6.58	8.7	0.16	0.46	44.9
$Ru_1Ir_{0.49}$	3	Ru	69.3	0.82	52.1	1.9	14.48	22.1
		Ir	64.9	0.94	47.7	1.2	15.06	24.6
RuO_2	3	Ru	133.8	7.36	100.0	2.4	24.04	23.5
IrO_2	3	Ir	134.0	46.4	55.0	5.0	27.6	55.2

Initial: Mass of metals deposited on carbon cloth.

On WE: Mass of metals remained on carbon cloth (working electrode) after catalysis.

In electrolyte: Mass of metals dissolved in the electrolyte.

On CE: Mass of metals redeposited on the counter electrode.

Peeled from WE: Mass of metals in catalysts peeled from the working electrode, calculated by mass balance: Peeled from WE = Initial – On WE – In electrolyte – On CE. The Ru/Ir ratios in the pealed meals are close to those in the pristine catalysts. The Ag contents were lower than expected, due to systematic errors associated with the low Ag concentration.

Undissolved: Undissolved metal ratios, calculated by mass balance:

Undissolved = (Initial – In electrolyte – On CE)/Initial × 100%

Table S7. O 1*s* XPS fitting results of $Ag_{0.19}Ru_1Ir_{0.48}$ after chronoamperometric testing at 1.74 V for different times. (spectra, Fig. 4i). The table shows peak positions for each O species in the catalysts. The values in parentheses are percentages of the specific O species among all O species in the catalyst, excluding physisorbed H_2O.

$Ag_{0.19}Ru_1Ir_{0.48}$	M-H_2O/eV	M-OH/eV	M-O-M/eV
2 h	532.9	532	–
10 h	533.2	532	–
100 h	533.2	532.1 (91.33%)	530.1 (8.67 %)

Table S8. Ru 3*d* XPS fitting results of $Ag_{0.19}Ru_1Ir_{0.48}$ after chronoamperometric testing at 1.74 V for different times (spectra, Fig. 4j).

$Ag_{0.19}Ru_1Ir_{0.48}$	Ru^0/eV	$Ru^{3+/4+}$/eV	Ru^{6+}/eV
2 h	280	–	–
10 h	280	–	–
100 h	280.2 (80.8%)	281.2 (19.2%)	–

Table S9. Ir 4*f* XPS fitting results of $Ag_{0.19}Ru_1Ir_{0.48}$ after chronoamperometric testing at 1.74 V for different times (spectra, Fig. S26).

$Ag_{0.19}Ru_1Ir_{0.48}$	Ir^0/eV	Ir^{4+}/eV	Ir^{6+}/eV
2 h	60.7	–	–
10 h	60.4	–	–
100 h	–	61.5	–

Table S10. Ag Auger electron spectrum fitting results of $Ag_{0.19}Ru_1Ir_{0.48}$ after chronoamperometric testing at 1.74 V for different times (spectra, Fig. S14 and S27).

$Ag_{0.19}Ru_1Ir_{0.48}$	Ag^0/eV	Ag^+/eV
2 h	358.2	–
10 h	357.9	–
100 h	–	355

Table S11. O 1*s* XPS fitting results of $Ag_{0.06}Ru_1Ir_{0.47}$ after chronoamperometric testing at 1.6 V for different times. The table shows peak positions for each O species in the catalysts. The values in parentheses are percentages of the specific O species among all O species in the catalyst, excluding physisorbed H_2O.

$Ag_{0.06}Ru_1Ir_{0.47}$	M-H_2O/eV	M-OH/eV	M-O-M/eV
2 h	532.9	532.1 (94.84%)	529.9 (5.16%)
10 h	533	531.9 (91.14%)	530 (8.86%)
100 h	533	531.7 (90.51%)	530.1 (9.49%)

Table S12. O 1*s* XPS fitting results of $Ru_1Ir_{0.49}$ after chronoamperometric testing at 1.6 V for different times. The table shows peak positions for each O species in the catalysts. The values in parentheses are percentages of the specific O species among all O species in the catalyst, excluding physisorbed H_2O.

$Ru_1Ir_{0.49}$	M-H_2O/eV	M-OH/eV	M-O-M/eV
2 h	533.1	532 (95.35%)	530.1 (4.65%)
10 h	533.2	532 (92.8%)	530 (7.2%)
100 h	533	531.6 (81.25%)	530 (18.75%)

Table S13. Ru 3*d* XPS fitting results of $Ru_1Ir_{0.49}$ after chronoamperometric testing at 1.6 V for different times. The table shows peak positions for each oxidation state species of Ru in the catalysts. The values in parentheses are percentages of the specific Ru oxidation state among all Ru species in the catalyst.

$Ru_1Ir_{0.49}$	Ru^0/eV	$Ru^{3+/4+}$/eV	Ru^{6+}/eV
2 h	280.2 (82.86%)	281.6 (17.14%)	–
10 h	280 (55.7%)	281.1 (40.2%)	282.3 (4.1%)
100 h	280.1 (54.5%)	281.1 (41.3%)	282.3 (4.2%)

Table S14. ICP-MS results of the $Ni_{0.22}Ru_1Ir_{0.48}$ and $Co_{0.25}Ru_1Ir_{0.46}$ catalysts.

Catalysts	Ru / Atomic%	Ir / Atomic%	Ni / Atomic%	Co / Atomic%
$Ni_{0.22}Ru_1Ir_{0.48}$	58.8	28.2	13	–
$Co_{0.25}Ru_1Ir_{0.46}$	58.5	26.9	–	14.6

Table S15. Ratios of dissolved metals from the $Cu_{0.16}Ru_1Ir_{0.47}$ catalyst after chronopotentiometric testing at 10 mA cm^{-2} for 200 h, measured by ICP-MS.

$Cu_{0.16}Ru_1Ir_{0.47}$	Initial/μg	On WE/μg	In electrolyte/μg	On CE/μg	Peeling/μg	Undissolved /%
Ru	23.58	18.1	2.63	0.74	2.11	85.8
Ir	20.97	17.1	1.86	0.32	1.69	89.7
Cu	2.37	1.52	0.22	0.23	0.4	81.3

Initial: Mass of metals deposited on carbon cloth.

On WE: Mass of metals remained on carbon cloth (working electrode) after catalysis.

In electrolyte: Mass of metals dissolved in the electrolyte.

On CE: Mass of metals redeposited on the counter electrode.

Peeled from WE: Mass of metals in catalysts peeled from the working electrode, calculated by mass balance: Peeled from WE = Initial – On WE – In electrolyte – On CE. The Ru/Ir ratios in the pealed meals are close to those in the pristine catalysts. The Ag contents were lower than expected, due to systematic errors associated with the low Ag concentration.

Undissolved: Undissolved metal ratios, calculated by mass balance:

Undissolved = (Initial – In electrolyte – On CE)/Initial × 100%

Table S16. Comparison of current density and mass activity at 2.0 V PEMWE with different anode catalysts operated at 80 °C.

Catalyst	Current density at 2 V (A cm^{-2})	Mass activity at 2 V (A mg_{Ru+Ir}^{-1})	Reference
$Ag_{0.19}Ru_1Ir_{0.48}$	4.99	5.67	This work
$(RuIr)O_2/C$	4.96	19.84	[32]
Com.IrO_2	3.17	12.68	

Table S17. The voltage degradation rate of catalysts (potential increase per hour) in PEMWE at high current densities. This table shows a comparison of $Ag_{0.19}Ru_1Ir_{0.48}$||Pt/C catalyst with state-of-the-art catalysts reported in the literature.

Catalysts	Current density ($A\ cm^{-2}$)	Voltage degradation rate ($\mu V\ h^{-1}$)	Ref.
$Ag_{0.19}Ru_1Ir_{0.48}$||Pt/C	1	0.93	This work
RuIr-NC||Pt/C	0.01	618	33
In-RuO_2||Pt/C	0.1	140	34
IrO_x-3Nd||Pt/C	1	55.8	35
Ta-RuO_2||Pt/C	1	14	36
RIE-Ir-CeO_x	3	1.33	37
Ir-O-Mn||Pt/C	1	20	38

Table S18. Summary of Ru, Ir, and Ag dissolution rate of $Ag_{0.19}Ru_1Ir_{0.48}$ catalyst in PEMWE working at 1 A cm^{-2}, measured by ICP.

$Ag_{0.19}Ru_1Ir_{0.48}$	Initial/μg	Dissolution concentration /μg L^{-1}	Dissolution percentage / %	Percentage dissolution rate / % h^{-1}
Ru	609	5.1	0.84	5.5×10^{-4}
Ir	280	2.2	0.79	5.2×10^{-4}
Ag	110	1.2	1.09	7.2×10^{-4}

Table S19. Calculation of the stability numbers (*S*-number) of different catalysts based on the chronopotentiometric results and metal dissolution measured by ICP-MS.

Catalysts	t/h	Q/C	n_{O2}/mol	n_{Ru}/mol	n_{Ir}/mol	S-number
$Ag_{0.19}Ru_1Ir_{0.48}$	1500	5.4×10^{6}	14.0	5.0×10^{-8}	1.1×10^{-8}	2.3×10^{8}
$Ru_1Ir_{0.49}$	0.4	108	2.8×10^{-4}	1.2×10^{-6}	5.1×10^{-7}	166
RuO_2	0.2	51.8	1.3×10^{-4}	2.2×10^{-6}	–	60
IrO_2	0.3	86.4	2.2×10^{-4}	–	7.7×10^{-7}	287

Table S20. Summary of the relative percentages of lattice oxygen (M-O-M), oxidized Ru (Ru^{4+}), and oxidized Ir (Ir^{4+}) derived from XPS after the start-stop testing.

$Ag_{0.19}Ru_{1}Ir_{0.48}$	M-O-M/%	Ru^{4+}/%	Ir^{4+}/%
After start-stop cycles	9.6	2.8	7.0

Table S21. Values and error bars of electrolyte pH.

Electrolyte	pH (Mean)	Standard deviation
0.1 M $HClO_4$	1.0	0.01

References

1 Lin, C. *et al.* In–situ reconstructed Ru atom array on α–MnO_2 with enhanced performance for acidic water oxidation. *Nat. Catal.* **4**, 1012–1023, (2021).

2 Hao, S. *et al.* Dopants fixation of ruthenium for boosting acidic oxygen evolution stability and activity. *Nat. Commun.* **11**, 5368, (2020).

3 Zhang, D. *et al.* Construction of Zn–doped RuO_2 nanowires for efficient and stable water oxidation in acidic media. *Nat. Commun.* **14**, 2517, (2023).

4 Hu, C. *et al.* Misoriented high-entropy iridium ruthenium oxide for acidic water splitting. *Sci. Adv.* **9**, eadf9144, (2023).

5 Liu, S. *et al.* Structurally–distorted RuIr–based nanoframes for long–duration oxygen evolution catalysis. *Adv. Mater.* **35**, 2305659, (2023).

6 Wen, Y. *et al.* Stabilizing highly active Ru sites by suppressing lattice oxygen participation in acidic water oxidation. *J. Am. Chem. Soc.* **143**, 6482–6490, (2021).

7 Shi, Z. *et al.* Customized reaction route for rthenium oxide towards stabilized water oxidation in high–performance PEM electrolyzers. *Nat. Commun.* **14**, 843, (2023).

8 Hao, S. *et al.* Torsion strained iridium oxide for efficient acidic water oxidation in proton exchange membrane electrolyzers. *Nat. Nanotechnol.* **16**, 1371–1377, (2021).

9 Liu, H. *et al.* Eliminating over-oxidation of ruthenium oxides by niobium for highly stable electrocatalytic oxygen evolution in acidic media. *Joule* **7**, 558–573, (2023).

10 Wu, Z.-Y. *et al.* Non–iridium–based electrocatalyst for durable acidic oxygen evolution reaction in proton exchange membrane water electrolysis. *Nat. Mater.* **22**, 100–108, (2022).

11 Wen, Y. *et al.* Introducing brønsted acid sites to accelerate the bridging–oxygen–assisted deprotonation in acidic water oxidation. *Nat. Commun.* **13**, 4871, (2022).

12 Li, L. *et al.* Compensating electronic effect enables fast site–to–site electron transfer over ultrathin RuMn nanosheet branches toward highly electroactive and stable water splitting. *Adv. Mater.* **33**, 2105308, (2021).

13 Zhang, H., Liu, L., Pei, L., Wang, D. & Wang, X. Highly active and stable nitrogen-doped ruthenium oxide/titanium nitride composite anode electrocatalyst for practical proton exchange membrane water electrolyzers. *Adv. Energy Mater.*, 2406074, (2025).

14 Qi, M. *et al.* Single-atom Ru-triggered lattice oxygen redox mechanism for enhanced acidic water oxidation. *J. Am. Chem. Soc.*, doi: 10.1021/jacs.1025c05752, (2025).

15 Wang, H. *et al.* Atomic Ga triggers spatiotemporal coordination of oxygen radicals for efficient water oxidation on crystalline RuO_2. *Nat. Commun.* **16**, 3976, (2025).

16 Wu, H. *et al.* Engineering high-density microcrystalline boundary with V-doped RuO_2 for high-performance oxygen evolution in acid. *Nat. Commun.* **16**, 4482, (2025).

17 Song, Y. *et al.* Sub-4 nm Ru-RuO_2 schottky nanojunction as a catalyst for durable acidic water oxidation. *J. Am. Chem. Soc.* **147**, 13775-13783, (2025).

18 Xue, Z.-H. *et al.* Simple and scalable introduction of single-atom Mn on RuO_2 electrocatalysts for oxygen evolution reaction with long-term activity and stability. *J. Am. Chem. Soc.* **147**, 17839–17848, (2025).

19 Li, R. *et al.* IrW nanochannel support enabling ultrastable electrocatalytic oxygen evolution at 2 A cm^{-2} in acidic media. *Nat. Commun.* **12**, 3540, (2021).

20 Miao, X. *et al.* Quadruple perovskite ruthenate as a highly efficient catalyst for acidic water oxidation. *Nat. Commun.* **10**, 3809, (2019).

21 Yan, H., Jiang, Z., Deng, B., Wang, Y. & Jiang, Z. J. Ultrathin carbon coating and defect engineering promote RuO_2 as an efficient catalyst for acidic oxygen evolution reaction with super–high durability. *Adv. Energy Mater.* **13**, 2300152, (2023).

22 Zhou, G. *et al.* Spin–related symmetry breaking induced by half–disordered hybridization in $Bi_xEr_{2-x}Ru_2O_7$ pyrochlores for acidic oxygen evolution. *Nat. Commun.* **13**, 4106, (2022).

23 Zheng, X. *et al.* Ir–Sn pair–site triggers key oxygen radical intermediate for efficient acidic water oxidation. *Sci. Adv.* **9**, eadi8025, (2023).

24 Qin, Y. *et al.* RuO_2 electronic structure and lattice strain dual engineering for enhanced acidic oxygen evolution reaction performance. *Nat. Commun.* **13**, 3784, (2022).

25 Chen, D. *et al.* Bicontinuous RuO_2 nanoreactors for acidic water oxidation. *Nat. Commun.* **15**, 3928, (2024).

26 Shi, Z. *et al.* Confined Ir single sites with triggered lattice oxygen redox: Toward boosted and sustained water oxidation catalysis. *Joule* **5**, 2164–2176, (2021).

27 Wang, J. *et al.* Single–site Pt–doped RuO_2 hollow nanospheres with interstitial C for high–performance acidic overall water splitting. *Sci. Adv.* **8**, eabl9271, (2022).

28 Sun, P. *et al.* Designing 3d transition metal cation–doped $MRuO_x$ as durable acidic oxygen evolution electrocatalysts for PEM water electrolyzers. *J. Am. Chem. Soc.* **146**, 15515–15524, (2024).

29 Zhao, G. *et al.* Metallic Ru–Ru interaction in ruthenium oxide enabling durable proton exchange membrane water electrolysis. *Adv. Mater.* **36**, 2404213, (2024).

30 Xie, Y. *et al.* Electrochemical leaching of Ni dopants in IrRu alloy electrocatalyst boosts overall water splitting. *Adv. Funct. Mater.* **34**, 2406351, (2024).

31 Xu, Y. *et al.* Strain–modulated Ru–O covalency in Ru-Sn oxide enabling efficient and stable water oxidation in acidic solution. *Angew. Chem. Int. Ed.* **63**, e202316029, (2024).

32 Park, Y. *et al.* Atomic-level Ru-Ir mixing in rutile-type $(RuIr)O_2$ for efficient and durable oxygen evolution catalysis. *Nat. Commun.* **16**, 579, (2025).

33 Wu, D. *et al.* Efficient overall water splitting in acid with anisotropic metal nanosheets. *Nat. Commun.* **12**, 1145, (2021).

34 Wang, Y. *et al.* Breaking the Ru–O–Ru symmetry of a RuO_2 catalyst for sustainable acidic water oxidation. *Angew. Chem. Int. Ed.* **63**, e202316903, (2023).

35 Zhang, N. *et al.* Local oxygen vacancy–mediated oxygen exchange for active and durable acidic water oxidation. *Angew. Chem. Int. Ed.*, e202503246, (2025).

36 Zhang, J. H. *et al.* Tantalum–stabilized ruthenium oxide electrocatalysts for industrial water electrolysis. *Science* **387**, 48–55, (2025).

37 Wenjuan Shi *et al.* Ultrastable supported oxygen evolution electrocatalyst formed by ripeninginduced embedding. *Science* **387**, 791-796, (2025).

38 Wang, D. *et al.* Ir-O-Mn embedded in porous nanosheets enhances charge transfer in low-iridium PEM electrolyzers. *Nat. Commun.* **16**, 181, (2025).
