# Supplementary material for: Oxidation-resistant AgRuIr alloy nanocages for efficient and enduring oxygen evolution in proton exchange membrane electrolysis
Source: Nat Commun. 2026 Apr 15;17:5195. doi: 10.1038/s41467-026-71943-6 (PMC13254247; doi:10.1038/s41467-026-71943-6)
Supplement: Supplementary file 3 — Description of Additional Supplementary Files [file 41467_2026_71943_MOESM3_ESM.pdf]

Description of Additional Supplementary Files

File Name: Supplementary Data 1

Description: The optimized structures of AgRuIr and RuIr models for DFT calculations (Fig. 5).
