# Supplementary material for: Oxidation-resistant AgRuIr alloy nanocages for efficient and enduring oxygen evolution in proton exchange membrane electrolysis
Source: Nat Commun. 2026 Apr 15;17:5195. doi: 10.1038/s41467-026-71943-6 (PMC13254247; doi:10.1038/s41467-026-71943-6)
Supplement: Supplementary file 2 — Peer Review File [file 41467_2026_71943_MOESM2_ESM.pdf]

nature portfolio

Peer Review File

Oxidation-Resistant AgRuIr Alloy Nanocages for Efficient and Enduring Oxygen Evolution in Proton Exchange Membrane Electrolysis

Open Access This file is licensed under a Creative Commons Attribution 4.0 International License, which permits use, sharing, adaptation, distribution and reproduction in any medium or format, as long as you give appropriate credit to the original author(s) and the source, provide a link to the Creative Commons license, and indicate if changes were made. In the cases where the authors are anonymous, such as is the case for the reports of anonymous peer reviewers, author attribution should be to 'Anonymous Referee' followed by a clear attribution to the source work. The images or other third party material in this file are included in the article's Creative Commons license, unless indicated otherwise in a credit line to the material. If material is not included in the article's Creative Commons license and your intended use is not permitted by statutory regulation or exceeds the permitted use, you will need to obtain permission directly from the copyright holder. To view a copy of this license, visit http://creativecommons.org/licenses/by/4.0/.

REVIEWER COMMENTS

Reviewer #1 (Remarks to the Author):

This manuscript reports the synthesis of AgRuIr alloy nanocages as highly efficient and durable OER catalysts for PEM water electrolysis. Using XPS, in situ Raman spectroscopy, and HRTEM, the authors convincingly demonstrate that the alloy nanocages remain intact under high OER potentials without converting to oxides, and that the active sites consist of an oxygenated metallic surface. This retention of the metallic state during catalysis effectively prevents the over-oxidation of Ru or Ir into soluble high-valent species, a common degradation pathway for conventional Ru- or Ir-based oxide catalysts, enabling exceptional catalytic stability in PEM water electrolysis. Particularly impressive is the demonstration of stable operation for 1500 hours at 1.73 V and 1 A cm-2, with a voltage degradation rate of only 0.93 µV/h and metal dissolution rates as low as 0.5-0.7% per thousand hours. These results highlight the effectiveness of the unique material design strategy. Through combined experimental characterization and theoretical calculations, the authors provide in-depth insight into the critical role of Ag in enhancing both the oxidation resistance and catalytic activity of the alloy. The arguments are rigorous and well-supported by the data, and the work presents a novel design for acidic OER catalysts. The data shown in both the main text and the Supporting Information are comprehensive and robust. Overall, I feel the quality of the research meets the high standards of Nature Communications. This manuscript is acceptable for publication after the authors address the following points.

1. Figure 2d shows the coexistence of FCC, HCP, and amorphous structures in the AgRuIr alloy nanocages. The authors marked a few areas with different phases, but only included two Fourier diffractograms in Figures 2e and 2f. It is recommended to add all FFT diffraction patterns for these specifically labeled FCC and HCP regions in the Supporting Information.
2. In Figure 4b-h, the authors compare the stability and metal dissolution rates of the ternary Ag-Ru-Ir alloy nanocages with those of binary AgRu and RuIr alloy catalysts. To better demonstrate the advantages of the ternary alloy, it is suggested to also test the stability and dissolution rates of another binary alloy catalyst, specifically AgIr alloy.
3. Figure 4b compares the dissolution rates of various catalysts: RuO_2, IrO_2, RuIr, AgRu, and AgRuIr. Besides quantifying the dissolution rate of each catalyst via ICP-MS, are there other intuitive ways to observe metal dissolution, such as monitoring changes in the average particle size?
4. The XPS analysis (Figure 4i, j) of the $Ag_{0.19}Ru_1Ir_{0.48}$ catalyst at various intervals provides insights into its oxidation resistance. To better confirm the role of Ag in preventing oxidation, it is also necessary to conduct valence state analysis for the Ag-free RuIr alloy catalyst at different intervals.
5. Fig. 4k and Fig. S21 show HAADF-STEM images of the $Ag_{0.19}Ru_1Ir_{0.48}$ catalyst after stability testing. The results verify that the nanocages retain their metallic state and show both FCC and HCP phases. Since the nanocages initially have amorphous regions, it is important to clearly label these areas in the post-catalysis STEM images. Are there any changes in the crystal structure of the nanocages?
6. The authors demonstrate that the metastable AgRuIr alloy phase is essential for its outstanding OER performance. To better support this hypothesis, it is essential to evaluate the OER activity and stability of the sample after calcination at 500 °C. Since the metastable phase transforms into a stable, phase-separated Ru-Ir/Ag structure after calcination, the catalyst would likely lose its OER activity and durability. Experimental data should be supplemented.

7. The results of the DFT calculations (Figure 5) should be discussed in more detail. For example, the Gibbs free energy profiles indicate that the OER energy barrier of the AgRuIr catalyst is reduced when its surface is covered by oxygen. What is the reason for this phenomenon, i.e., why surface oxygen coverage promotes the transformation of *O to *OOH?

Reviewer #2 (Remarks to the Author):

In this manuscript, Wang et al. report a novel AgRuIr alloy nanocage catalyst for the acidic oxygen evolution reaction (OER), demonstrating excellent catalytic activity and durability in PEMWE. This work presents a conceptually distinct approach: whereas conventional acidic OER catalysts typically rely on Ru or Ir oxides, the authors demonstrate a high-performance metallic catalyst. Their key finding is that the metallic state of the catalyst is preserved under operating conditions. This stability is attributed to alloying Ru and Ir with Ag, which reduces the overall oxophilicity of the catalyst and prevents oxygen from incorporating into the metal lattice. The oxidation resistance of the catalyst has been reliably confirmed through robust experimental data, including in-situ Raman spectroscopy and quasi-in-situ XPS. The oxidation resistance mechanism has been further validated by DFT calculations. This novel design results in remarkable PEMWE performance (1 A cm-2@1.73V) and durability (< 1 µV degradation per hour). Additionally, the synthesis protocol is interesting and unconventional, to my knowledge, and seems potentially extendable to other alloy systems, including those with immiscible components. Therefore, I recommend its publication in Nature Communications after the authors address the following concerns.

1. The vacancy-diffusion-assisted alloying mechanism is intriguing. To better understand this process, I suggest including a schematic diagram of the synthesis procedure in the supporting information, showing how vacancy migration aids the alloying of immiscible elements.

2. In the vacancy-diffusion-assisted alloying, the etching process is important because it directly correlates with the formation of vacancies. In this regard, the authors should include a discussion on how etching parameters, such as etchant concentration, etching time, and temperature, influence alloy formation.

3. Figure 2: While HAADF-STEM and phase analysis have revealed the distorted/disordered lattice arrangements in the metastable AgRuIr alloy nanocages, the authors further provided Ru K-edge EXAFS data to confirm the complex local atomic structure of the nanocrystals. I suggest that the authors include Ir L3-edge EXAFS data to better support the discussion on the local atomic structure.

4. Figure 4: The O1s and Ru 3d XPS spectra of AgRuIr after long-term OER testing were investigated to show that lattice oxygen and oxidized Ru were absent. How about the Ag-free, binary RuIr alloy? A similar study on RuIr alloy catalysts will be helpful in understanding the critical role of Ag in suppressing metal oxidation during catalysis.

5. In Figure 4b, the authors compared the metal dissolution levels of Ag.19Ru1Ir0.48 to those of many other catalysts, including binary Ag0.2Ru1 and Ru1Ir0.49. How about the metal dissolution levels for AgIr alloy catalysts?

6. Ag0.06Ru1Ir0.47 is also an AgRuIr alloy catalyst. Why is its stability significantly lower than that of the Ag0.19Ru1Ir0.48 counterpart? Please explain how such a minor difference in Ag content affects the stability of the catalyst.

7. In PEMWE testing, Figure 6b shows that the cell voltage was 1.73 V to achieve the current density of 1 A cm-2, while Figure 6c indicates that the cell voltage was approximately 2 V to achieve the same current density. What is the reason for the inconsistency in the cell voltage? A more detailed discussion on this data is needed to avoid confusion

8. The EIS data in Figure 3e were fitted by an equivalent circuit shown in Table S4. The reason for choosing this model should be added to the text. Besides, the physical meanings of Rs, CPE, and Rct should be clearly explained and discussed.

Reviewer #3 (Remarks to the Author):

This manuscript reports the synthesis of metastable Ag0.19Ru1Ir0.48 alloy nanocages that the authors claim remain metallic in the bulk under harsh acidic OER conditions, thereby reducing oxophilicity and suppressing over-oxidation. The catalyst delivers high intrinsic activity (e.g., 200 mA cm^{-2} at 1.463 V vs RHE) and notable durability, including PEMWE operation at 1 A cm^{-2} and 1.73 V with minimal degradation (0.93 μV h^{-1} over 1500 h) and ultralow dissolution (0.5–0.7 % k h^{-1}). The nanocage synthesis route and the strategy to alloy otherwise immiscible components are innovative. Overall, the performance metrics and especially the long-term durability are impressive and, if validated, would represent a meaningful advance for acid OER and PEMWE.

Major Remarks
1)The manuscript's central hypothesis that the Ag0.19Ru1Ir0.48 nanocages maintain a metallic bulk state throughout OER operation avoiding overoxidation is not fully supported by the presented XPS data.
Figure S18 shows that the Ir 4f spectrum after 100 h at 1.74 V corresponds with an asymmetric line shape typical of IrO_2 (https://doi.org/10.1002/sia.7215) rather than metallic Ir. This suggests significant surface oxidation or complete oxidation of Ir (considering shell thickness of 3.4 nm), contradicting the claim of persistent metallic character. Fig S22 also confirms thitrend for both Ru and Ir after CA at 200mA/cm2
I also suggest to add to Figure 4 Ir 4f and Ag Auger spectra at 2 h, 10 h, and 100 h after CA at 1.74V, and add a table in the SI summarizing binding energies and relative concentrations for O, Ru, Ir, and Ag.

2) It is undeniable that the results for Ag0.19Ru1Ir0.48 nanocages in terms of OER activity and stability are very good, and considering the no preservation of metallic bulk from XPS data, the authors should consider that high activity and stability could be related to other mechanism rather than to metallic preservation. Recent work (Nature Communications 2025, DOI: 10.1038/s41467-025-55910-1) shows that Ru–Ir atomic mixing in rutile-type oxides can deliver similar performance via optimized oxygen adsorption. The authors should consider this alternative mechanism since the

PEMWE activity results shown in this article are very similar: 3 A/cm² @ 2 V vs. 3.17 A/cm² from the previously cited Nat Comm 2025.

3) The authors use carbon cloth for OER electrochemical tests at potentials up to 1.74 V vs RHE and high current densities (200 mA/cm2). Carbon substrates are not electrochemically inert under such acid OER conditions, they can oxidize, and contribute with parasitic currents, thereby confounding activity/stability attribution to the catalyst. I strongly recommend repeating key measurements on a corrosion-resistant substrate, such as Ti mesh, and/or provide blank carbon-cloth controls at 1.74 V to quantify substrate contributions.
4) The PEMWE results substantially strengthen the manuscript by demonstrating that the exceptional half-cell stability of $Ag_{0.19}Ru_1Ir_{0.48}$does translate into record-level single-cell durability at 1 A cm^{-2}, supported by impressively low dissolution rates. Nevertheless, the claims of "practical applicability" would be more convincing if tempered by a clearer discussion of operating conditions, catalyst loading targets, and durability under dynamic operation. Rather than definitively establishing AgRuIr as a drop-in industrial anode catalyst, these results should be viewed as a compelling proof of concept for a new stabilization strategy for Ru-based PEMWE anodes, warranting further validation under more demanding, application-relevant conditions.
To further validate practical PEMWE durability, the catalyst should be evaluated under dynamic operation protocols that mimic renewable-driven electrolysis, including start–stop cycling (e.g., alternating 10–20 min operation at 1 A cm^{-2} with 10 min at open-circuit or load cycling between low and high current densities as corrosion stress tests, followed by recovery under steady operation. Also, monitoring voltage drift, high-frequency resistance (HFR), together with post-mortem MEA analysis (SEM , TEM, XPS) would directly demonstrate whether the exceptional stability persists under realistic, non-steady-state PEMWE conditions.

Minor Remarks
1) Provide mass-activity normalization (A mg^{-1}(Ru+Ir)) at 2.0 V for PEMWE tests. Benchmark against the $(RuIr)O_2$ Nature Communications study (DOI: 10.1038/s41467-025-55910-1): ≈3.17 A cm^{-2} at 2.0 V, mass activity ≈19.84 A mg^{-1}(Ru+Ir) at 2.0V.
2) I think the work on optimizing the catalyst preparation and compositionin terms of the Ru/Ir ratio and shell thickness is very good and deserves to be detailed in the main text, with reference to Figs. S4–S8.

Reviewer #4 (Remarks to the Author):

I co-reviewed this manuscript with one of the reviewers who provided the listed reports. This is part of the Nature Communications initiative to facilitate training in peer review and to provide appropriate recognition for Early Career Researchers who co-review manuscripts.

Reviewer #5 (Remarks to the Author):

The manuscript titled "Oxidation-Resistant AgRuIr Alloy Nanocages for Efficient and Enduring Oxygen

Evolution in PEM Water Electrolysis" reports the development of metastable AgRuIr alloy nanocages with high intrinsic OER activity and exceptional long-term stability under acidic PEMWE conditions. The authors propose that Ag lowers oxophilicity, while Ir enhances oxidation resistance through electronic hybridization. Notably, the nanocages exhibited 1500 hours of durability at 1 A/cm^2 in a full PEMWE device. If fully validated, this result would represent a meaningful advance toward durable, non-oxide OER catalysts. Overall, the work is timely and potentially impactful. However, to meet the standards of Nature Communications, several aspects of the mechanistic interpretation and experimental evidence require clarification.

1. The authors attribute the improved oxidation resistance to Ir "elevating the reduction potentials" of Ru and Ag within the alloy through electronic hybridization. This is an important claim, yet it is not currently substantiated by direct measurements or calculated electronic descriptors. The authors should provide quantitative evidence demonstrating how Ir modifies redox energetics in the alloy environment. Without such analysis, the mechanistic conclusion remains speculative.

2. XPS and Raman spectroscopy suggest an absence of bulk oxide formation. However, both techniques probe only limited depths and may not completely eliminate the possibility of subsurface oxidation, especially considering the ~3.8 nm shell thickness. The authors should explicitly discuss these limitations and, if possible, reference operando XAS or other, more bulk-sensitive evidence to reinforce their claim that the alloy remains metallic under elevated potentials.

3. The explanation for the enhanced performance focuses on the low oxophilicity of Ag. However, introducing Ag also imposes lattice strain and alters local coordination, both of which can significantly influence the d-band structure and active-site energetics. Therefore, the authors should discuss whether strain or structural disorder contributes comparably to the observed improvements.

4. The reported breakdown of the conventional *OH/*OOH scaling is intriguing. However, more detail is needed to determine if it is robust or model-dependent. The authors should clarify how sensitive the results are to surface *O coverage and determine whether the quasi-random alloy structure accurately represents realistic surface heterogeneity. Providing such clarification would improve confidence in the generality of the mechanistic conclusions.

Reviewer #1 (Remarks to the Author):

I am satisfied with the revision, and it can be accepted at the present state.

Reviewer #2 (Remarks to the Author):

The reviewer have carefully address the issues raised by the reviewer, and i think the manuscript are ready for publication on Nature Communications.

Reviewer #3 (Remarks to the Author):

I would like to thank the authors for their detailed reply and for addressing all of the improvements suggested, as well as for including new characterizations that further strengthen the conclusions of the article. The manuscript is acceptable for publication, however, I would like to highlight two points:

1)I would like to emphasize that, indeed, during the first hours of operation, the activity of the AgIrRu catalyst is associated with the metallic character of Ru and Ir. However, after 100 hours, the XPS data show that all Iridium has become oxidized, which is reasonable considering that the nanocage shell thickness is 3.8 nm, approximately the depth probed by XPS. What is important, and should be explicitly stated in the manuscript, is that the metallic state of Ru is preserved even after 1000 hours of operation, as confirmed by several techniques (XPS and FT EXAFS). The interaction between this metallic Ru and IrO_2 may be responsible for the catalyst's activity and long term stability.

2) Regarding the PEMWE experiments suggested in my Question 4, I appreciate that the authors followed all of our recommendations. Nevertheless, I would like to request further clarification on the following point to be addressed by the authors:

In the dynamic start-stop cycling experiments shown in Fig. S49, it is surprising how stable the voltage remains throughout the different cycles, with no apparent changes even during start cycle after OCP periods where a potential "jump" is usually observed, followed by a gradual decrease until stabilization. Could the authors provide a zoom-in of these results and/or the raw data?

Reviewer #4 (Remarks to the Author):

I co-reviewed this manuscript with one of the reviewers who provided the listed reports. This is part of the Nature Communications initiative to facilitate training in peer review and to provide appropriate recognition for Early Career Researchers who co-review manuscripts.

Reviewer #5 (Remarks to the Author):

My concerns have been well addressed, and the manuscript has been revised accordingly. Therefore, I recommend its publication.

Reviewer #3 (Remarks to the Author):

The authors have addressed the issues raised by the reviewers. The manuscript is ready for publication at the present state

Reviewer #4 (Remarks to the Author):

I co-reviewed this manuscript with one of the reviewers who provided the listed reports. This is part

of the Nature Communications initiative to facilitate training in peer review and to provide appropriate recognition for Early Career Researchers who co-review manuscripts.

Response to Reviewers

Dear Editor and Reviewers,

Thank you for your positive assessment and constructive feedback. Your suggestions have been invaluable in enhancing the mechanistic depth of our work. We have carefully revised the manuscript to address all comments, incorporating new discussion and experimental data as requested. Our detailed point-by-point responses to the reviewers' concerns are provided below.

Response to Reviewer #1:

Reviewer's Comments: This manuscript reports the synthesis of AgRuIr alloy nanocages as highly efficient and durable OER catalysts for PEM water electrolysis. Using XPS, in situ Raman spectroscopy, and HRTEM, the authors convincingly demonstrate that the alloy nanocages remain intact under high OER potentials without converting to oxides, and that the active sites consist of an oxygenated metallic surface. This retention of the metallic state during catalysis effectively prevents the over-oxidation of Ru or Ir into soluble high-valent species, a common degradation pathway for conventional Ru- or Ir-based oxide catalysts, enabling exceptional catalytic stability in PEM water electrolysis. Particularly impressive is the demonstration of stable operation for 1500 hours at 1.73 V and 1 A cm^{-2}, with a voltage degradation rate of only 0.93 μV/h and metal dissolution rates as low as 0.5-0.7% per thousand hours. These results highlight the effectiveness of the unique material design strategy. Through combined experimental characterization and theoretical calculations, the authors provide in-depth insight into the critical role of Ag in enhancing both the oxidation resistance and catalytic activity of the alloy. The arguments are rigorous and well-supported by the data, and the work presents a novel design for acidic OER catalysts. The data shown in both the main text and the Supporting Information are comprehensive and robust. Overall, I feel the quality of the research meets the high standards of Nature Communications. This manuscript is acceptable for publication after the authors address the following points.

Response: We sincerely thank the reviewer for their positive evaluation of our work and for recognizing the significance of our AgRuIr alloy catalyst design for the acidic OER. We are particularly encouraged by the reviewer's appreciation of our mechanistic studies and the long-term stability demonstrated within PEMWE. We have carefully considered each of the reviewer's insightful comments and have revised the manuscript accordingly to further enhance its quality and clarity. Below, we provide a point-by-point response to the specific technical concerns.

1. Figure 2d shows the coexistence of FCC, HCP, and amorphous structures in the AgRuIr alloy nanocages. The authors marked a few areas with different phases, but only included two Fourier diffractograms in Figures 2e and 2f. It is recommended to add all FFT diffraction patterns for these specifically labeled FCC and HCP regions in the Supporting Information.

Response: We thank the reviewer for this constructive suggestion. In accordance with this recommendation, we have provided the FFT diffraction patterns for all the FCC and HCP crystallites identified in **Fig. 2d.** These patterns have been presented in **Fig. S3** of the revised Supporting Information (and are also provided below for your convenience).

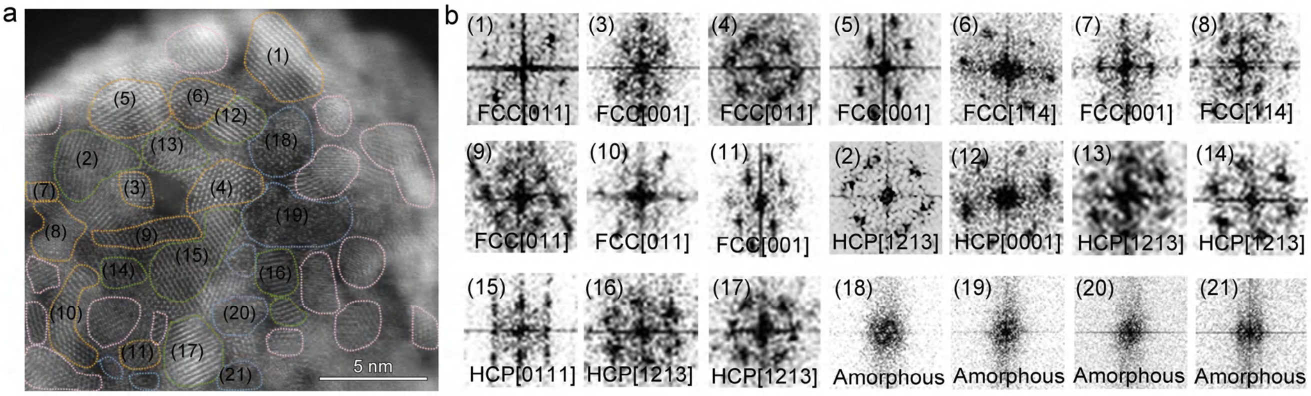

Fig. S3. Phase analysis of crystallites in $Ag_{0.19}Ru_{1}Ir_{0.48}$ alloy nanocages. (a) HAADF-STEM image of a representative nanocage. The yellow, green, blue, and pink dotted lines delineate the FCC, HCP, amorphous, and undefined (structure not resolved) crystallites, respectively. (b) FFT diffraction patterns corresponding to the FCC, HCP, and amorphous crystallites as labeled in (a).

2. In Figure 4b-h, the authors compare the stability and metal dissolution rates of the ternary Ag-Ru-Ir alloy nanocages with those of binary AgRu and RuIr alloy catalysts. To better demonstrate the advantages of the ternary alloy, it is suggested to also test the stability and dissolution rates of another binary alloy catalyst, specifically AgIr alloy.

Response: We thank the reviewer for this helpful suggestion. In response, we have investigated the OER activity, stability, and metal dissolution rates of a binary AgIr alloy catalyst to serve as an additional control for the ternary AgRuIr system. As the main text focuses primarily on Ru-based catalysts, these results have been incorporated into **Fig. S23** of the revised Supporting Information.

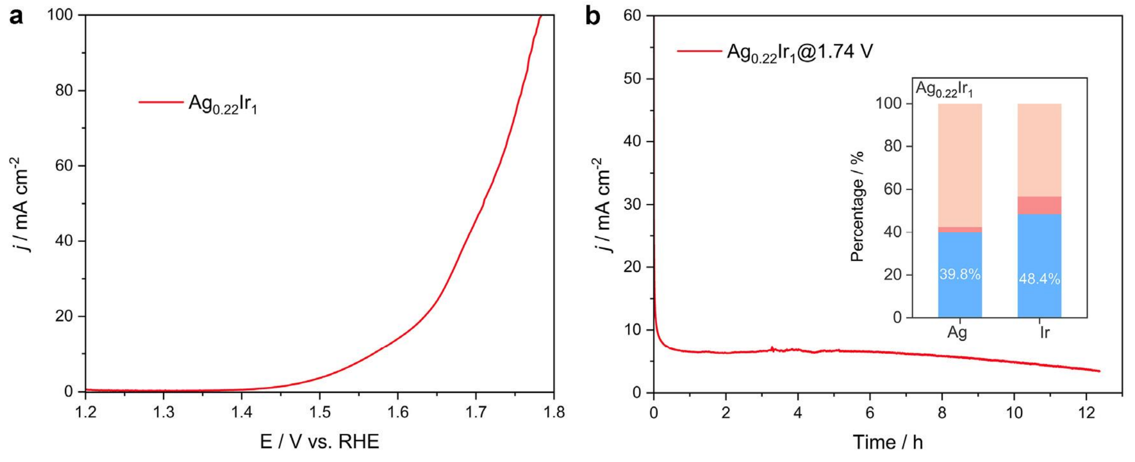

Fig. S23. Catalytic activity and stability of the AgIr alloy catalyst in acidic OER. (a) LSV curve in O_2-saturated 0.1 M $HClO_4$. (b) Chronoamperometric curve recorded at 1.74 V. Inset: Percentages of

dissolved Ag and Ir following chronoamperometric testing, as determined by ICP-MS. The yellow, pink, and blue bars represent the percentages of metals dissolved in the electrolyte, redeposited on the counter electrode, and remaining undissolved, respectively.

As illustrated by these results, the binary AgIr alloy exhibits poor stability during the OER. A significant decline in current density was observed at 1.74 V within the first 12 h of testing. ICP-MS analysis revealed that over 60% of both Ag and Ir dissolved during this period, which accounts for the rapid degradation in catalytic performance. These results further highlight the superiority of the ternary AgRuIr alloy composition in achieving exceptionally low metal dissolution rates and robust OER stability.

3. Figure 4b compares the dissolution rates of various catalysts: RuO_2, IrO_2, RuIr, AgRu, and AgRuIr. Besides quantifying the dissolution rate of each catalyst via ICP-MS, are there other intuitive ways to observe metal dissolution, such as monitoring changes in the average particle size?

Response: We thank the reviewer for this constructive comment. In this work, metal dissolution from the catalysts was primarily quantified using ICP-MS. The reviewer suggests an intuitive alternative approach: directly monitoring changes in the average particle size. In accordance with this recommendation, we have measured the average size of the AgRuIr alloy nanocages before and after chronopotentiometric testing at 200 mA cm^{-2} for 200 h. These results have been incorporated into **Fig. S20** of the revised Supporting Information. No significant change in the average particle size was observed, which is consistent with the enhanced oxidation resistance revealed by our XPS and ICP-MS analyses.

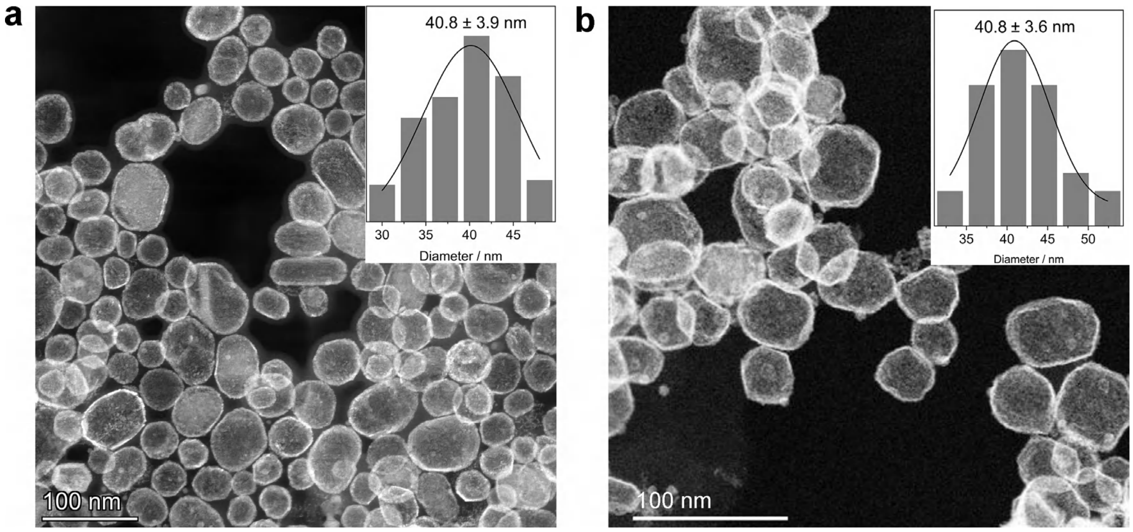

Fig. S20. Morphological and size change of the AgRuIr alloy nanocages during OER. (a) HAADF-STEM image of the as-synthesized nanocages. (b) HAADF-STEM image of the nanocages after chronopotentiometric testing at 200 mA cm^{-2} for 200 h.

In contrast, a phase-separated Ag-RuIr catalyst exhibited significant particle shrinkage during the OER. This control catalyst, synthesized via impregnation followed by annealing in a H_2 atmosphere,

exhibited an initial average particle size of 310 nm. Following chronopotentiometric testing at 10 mA cm^{-2} for 200 h, the average particle size decreased markedly from 310 nm to 170 nm. Although this catalyst maintained a virtually stable potential at 10 mA cm^{-2}, the substantial size reduction suggests significant metal dissolution during the OER. These results are now presented in **Fig. S19** of the revised Supporting Information.

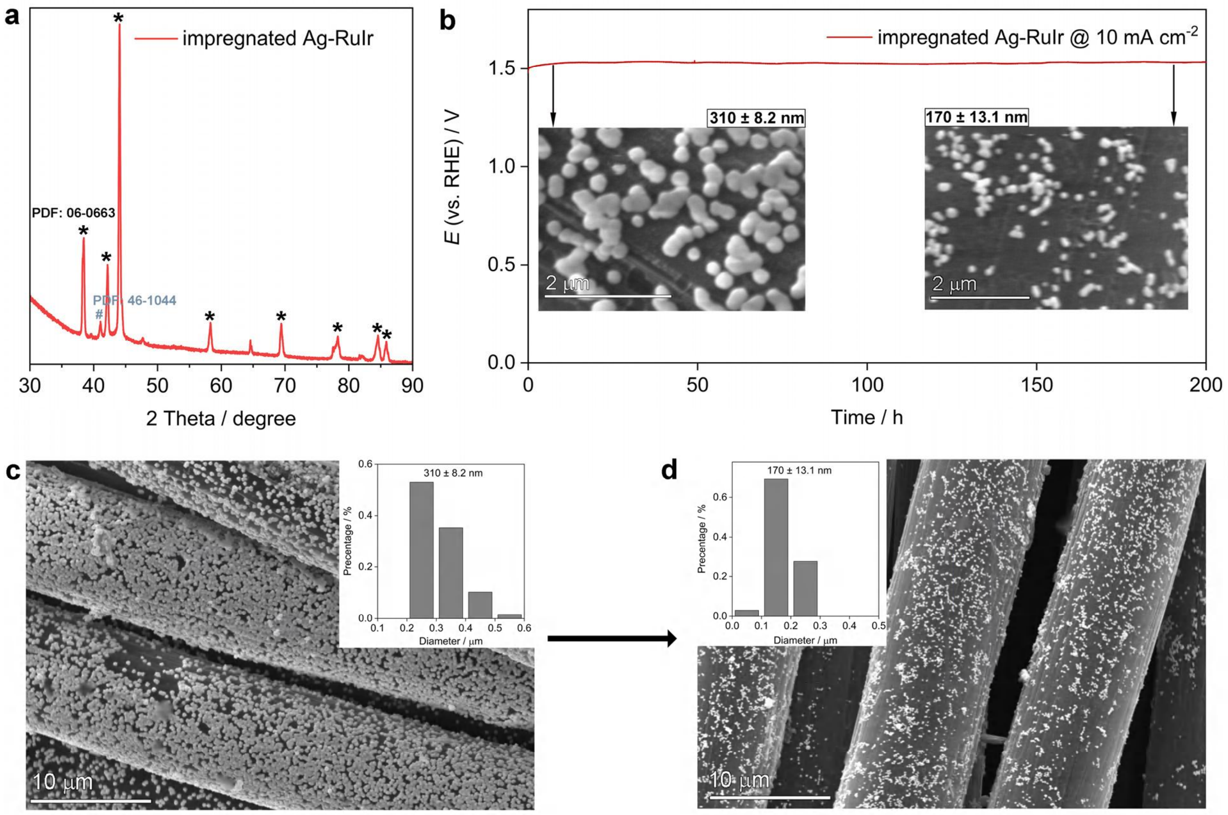

Fig. S19. Morphological and size change of a phase-separated Ag-RuIr catalyst during OER. The catalyst was synthesized via impregnation followed by annealing in a H_2 atmosphere. (a) XRD pattern of the catalyst, showing distinct signals from segregated Ag and RuIr phases. (b) Chronopotentiometric curve recorded at 10 mA cm^{-2}. Inset: SEM images of the catalyst nanoparticles before and after testing. (c, d) Low-magnification SEM images and corresponding particle size distributions before and after chronopotentiometric testing. While the potential remained relatively stable, the average particle size decreased from 310 nm to 170 nm, indicating significant oxidative dissolution. These results also indicate that average particle size can serve as an indicator of metal loss during the OER process.

Therefore, we agree with the reviewer that monitoring changes in average particle size serves as a valuable indicator of metal dissolution, complementing quantitative ICP-MS evaluations. The fact that our AgRuIr alloy nanocages retain their size after catalysis further supports the conclusion that these structures are highly resistant to oxidation under OER operating conditions. The corresponding discussion has been added to the revised manuscript:

Page 14: “Additionally, post-catalytic characterization showed negligible changes in average particle size, consistent with the minimal metal losses detected via ICP-MS (**Fig. S19** and **S20**).”

4. The XPS analysis (Figure 4i, j) of the $Ag_{0.19}Ru_1Ir_{0.48}$ catalyst at various intervals provides insights into its oxidation resistance. To better confirm the role of Ag in preventing oxidation, it is also necessary to conduct valence state analysis for the Ag-free RuIr alloy catalyst at different intervals.

Response: We thank the reviewer for the constructive suggestion. In **Fig. S33**, we provide the XPS spectra of the Ag-free $Ru_1Ir_{0.49}$ catalyst at various intervals during chronoamperometric OER testing at 1.6 V. Even at this relatively low potential (notably lower than the 1.74 V applied to the $Ag_{0.19}Ru_1Ir_{0.48}$ catalysts in **Fig. 4**i, j), both Ru and Ir underwent rapid oxidation. This is evidenced by the clear appearance of Ru^{6+} 3d peaks, Ir^{4+} 4f peaks, and O 1s peaks attributed to lattice oxygen. These results confirm the high susceptibility of the Ag-free RuIr catalyst to oxidation.

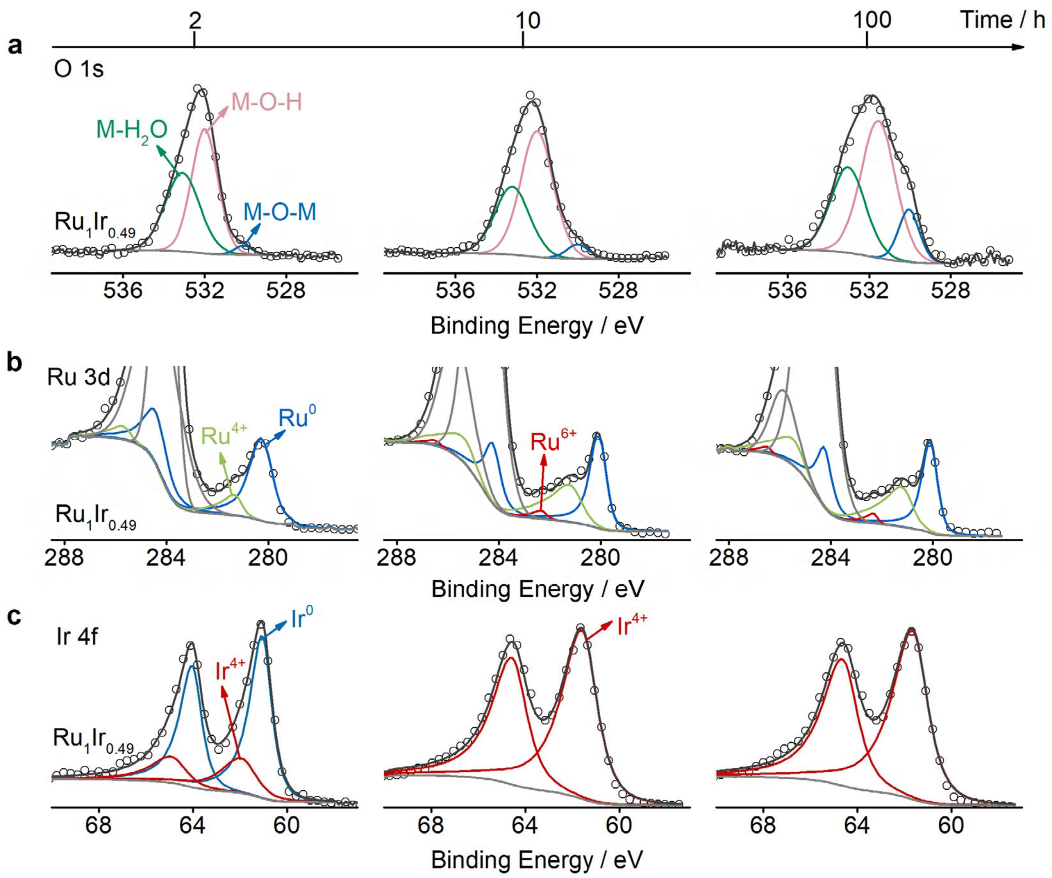

Fig. S33. XPS spectra of the $Ru_1Ir_{0.49}$ catalyst after chronoamperometric testing at 1.6 V for 2, 10, and 100 h. (a) O 1s. (b) Ru 3d. (c) Ir 4f.

To ensure a fair comparison at a potential identical to that used for the $Ag_{0.19}Ru_1Ir_{0.48}$ catalyst, we further investigated the Ru 3d and Ir 4f XPS of the Ag-free $Ru_1Ir_{0.49}$ catalyst after a short time (3 h) of chronoamperometric testing at 1.74 V. These results have been added to **Fig. S34** of the revised Supporting Information. Under these conditions, no Ru 3d or Ir 4f XPS signals were detectable, suggesting the complete loss of the RuIr catalyst due to severe oxidative dissolution.

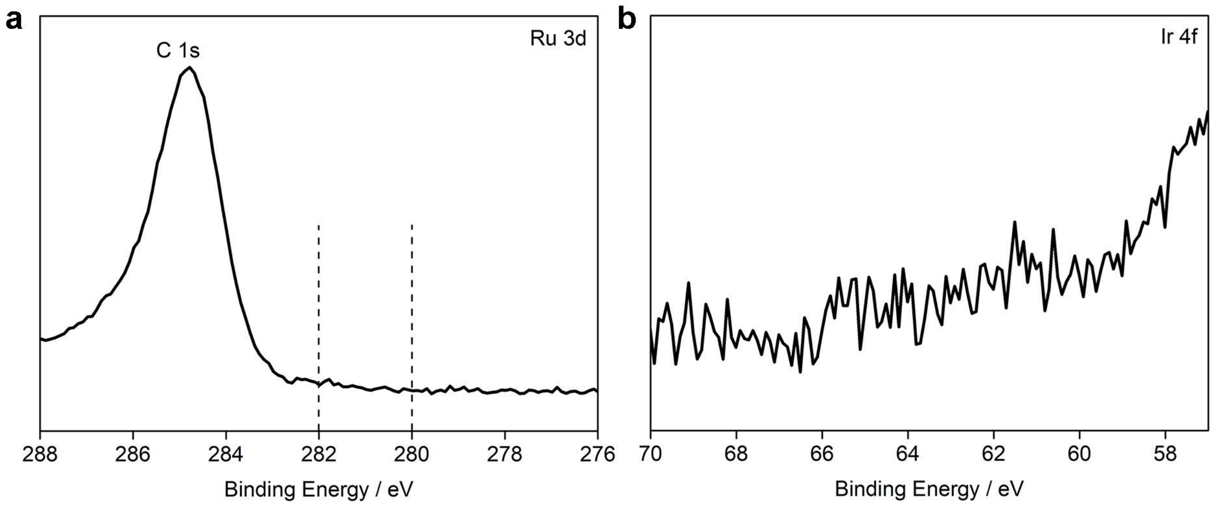

Fig. S34. XPS spectra of the $Ru_1Ir_{0.49}$ catalyst after chronoamperometric testing at 1.74 V for 3 h. (a) Ru 3d. The dotted lines indicate the expected positions for Ru 3d peaks. (b) Ir 4f. At this elevated potential, the Ag-free RuIr alloy catalysts underwent rapid degradation within 3 h, resulting in the absence of detectable XPS signals.

These collective results confirm that the incorporation of Ag is essential for enhancing the oxidation resistance of the AgRuIr alloy catalyst under OER operating potentials.

5. Fig. 4k and Fig. S21 show HAADF-STEM images of the $Ag_{0.19}Ru_1Ir_{0.48}$ catalyst after stability testing. The results verify that the nanocages retain their metallic state and show both FCC and HCP phases. Since the nanocages initially have amorphous regions, it is important to clearly label these areas in the post-catalysis STEM images. Are there any changes in the crystal structure of the nanocages?

Response: We thank the reviewer for this constructive suggestion. In response, we have performed a detailed structural examination of the crystallites within the $Ag_{0.19}Ru_1Ir_{0.48}$ alloy nanocages following the OER stability test. Our analysis reveals that the crystal structure of the nanocages remained virtually unchanged after catalysis. The nanocages are composed of crystallites with varying structures, including FCC, HCP, and amorphous regions. To provide further clarity, we have added the FFT diffraction patterns of representative crystallites to **Fig. S30** of the revised Supporting Information (also shown below).

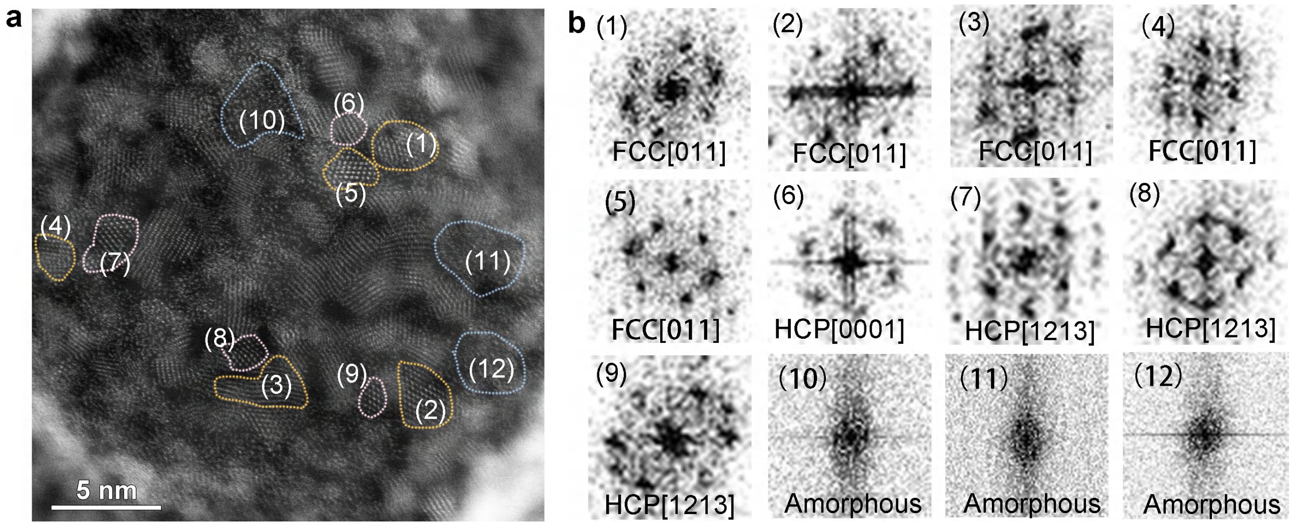

Fig. S30. Phase analysis of crystallites in $Ag_{0.19}Ru_1Ir_{0.48}$ nanocages after chronopotentiometric testing at 200 mA cm^{-2} for 200 h. (a) HAADF-STEM image of a representative nanocage post-catalysis. The labeled regions indicate the presence of FCC, HCP, and amorphous phases. (b) FFT diffraction patterns corresponding to the FCC, HCP, and amorphous crystallites as identified in (a).

6. The authors demonstrate that the metastable AgRuIr alloy phase is essential for its outstanding OER performance. To better support this hypothesis, it is essential to evaluate the OER activity and stability of the sample after calcination at 500 °C. Since the metastable phase transforms into a stable, phase-separated Ru-Ir/Ag structure after calcination, the catalyst would likely lose its OER activity and durability. Experimental data should be supplemented.

Response: We thank the reviewer for this helpful suggestion. We agree that the phase-separated Ru-Ir/Ag structure, obtained by annealing the $Ag_{0.19}Ru_1Ir_{0.48}$ alloy nanocages at 500 °C, provides a critical point of comparison for the metastable alloy phase. As hypothesized, the phase-separated structure exhibits a marked decrease in both OER activity and stability. These results have been incorporated into **Fig. S6** of the revised Supporting Information.

The phase-separated Ru-Ir/Ag catalyst displays significantly diminished OER activity, requiring an overpotential of 310 mV to reach 10 mA cm^{-2}. This is substantially higher than the 160 mV overpotential required by the metastable $Ag_{0.19}Ru_1Ir_{0.48}$ alloy nanocages. Furthermore, the annealed catalyst exhibited poor stability. During chronopotentiometric testing at 10 mA cm^{-2}, the potential increased rapidly within the first 10 h. These results clearly validate that the alloying of Ag is essential for enhancing both the OER activity and the stability of the AgRuIr catalyst.

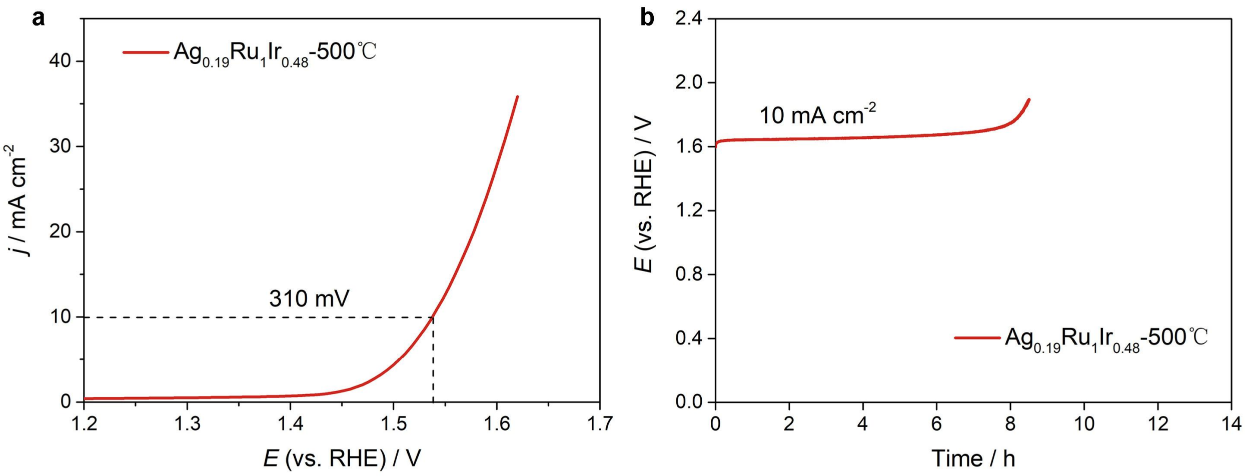

Fig. S6. OER performance of the phase-separated Ru-Ir/Ag catalyst obtained by annealing $Ag_{0.19}Ru_{1}Ir_{0.48}$ alloy nanocages at 500 °C. (a) LSV curves in O_2-saturated 0.1 M $HClO_4$. *iR* compensation level: 95%. Catalyst loading: 0.134 mg cm^{-2}. (b) Chronopotentiometric curve of the catalyst at 10 mA cm^{-2}.

7. The results of the DFT calculations (Figure 5) should be discussed in more detail. For example, the Gibbs free energy profiles indicate that the OER energy barrier of the AgRuIr catalyst is reduced when its surface is covered by oxygen. What is the reason for this phenomenon, i.e., why surface oxygen coverage promotes the transformation of *O to *OOH?

Response: We thank the reviewer for the opportunity to elaborate on the theoretical insights provided by our DFT calculations. In our study, we employed an oxygen-coverage model to simulate the actual surface state of the catalyst under OER operating conditions. This approach was motivated by our in-situ Raman spectroscopy investigation, which confirmed that the AgRuIr surface is primarily occupied by oxygenated species during catalysis.

The reduction in the OER energy barrier upon oxygen coverage can be attributed to the modulation of adsorption energies for the oxygenated intermediates. Specifically, the potential-determining step in the OER process is often the transformation of *O to *OOH. According to the theoretical framework established by Nørskov and co-workers (ref. 40), increasing the surface oxygen coverage destabilizes the adsorption energy of *O more significantly than that of *OOH. This relative destabilization of the *O intermediate reduces the Gibbs free energy barrier required for the formation of *OOH, thereby promoting the overall OER kinetics.

This trend is consistent with our AgRuIr alloy system, as validated by the DFT calculations. By incorporating an appropriate degree of oxygen coverage to mimic the actual catalytic interface, our model more accurately reflects the enhanced activity observed experimentally. To improve the clarity of the manuscript, we have expanded the discussion in the revised version to include these details:

Page 17: "Additionally, surface oxygen coverage (θ_{*O}) was also found to significantly influence OER

kinetics, aligning with the theoretical framework established by Nørskov and co-workers[40]. Given the high energy increase required for *O → *OOH, *O species tend to accumulate on the metal surface, reaching high coverage before the OER proceeds. Our DFT calculations demonstrate that with increasing θ_{*O}, the energy increase for the PDS (*O → *OOH) markedly decreases (**Fig. S46**). Specifically, at θ_{*O} = 3/4, the energy increase for this step reaches 2.10 eV at U = 0 and 0.87 eV at U = 1.23 V. This reduction upon oxygen coverage stems from the differential modulation of adsorption energies: increasing surface oxygen coverage destabilizes *O more significantly than *OOH. This relative destabilization reduces the energy increase required for *OOH formation, thereby promoting the overall OER kinetics. These results demonstrate that AgRuIr, characterized by a spontaneously formed surface *O layer at operating potentials, is highly efficient for OER catalysis"

Response to Reviewer #2:

Reviewer's comments: In this manuscript, Wang et al. report a novel AgRuIr alloy nanocage catalyst for the acidic oxygen evolution reaction (OER), demonstrating excellent catalytic activity and durability in PEMWE. This work presents a conceptually distinct approach: whereas conventional acidic OER catalysts typically rely on Ru or Ir oxides, the authors demonstrate a high-performance metallic catalyst. Their key finding is that the metallic state of the catalyst is preserved under operating conditions. This stability is attributed to alloying Ru and Ir with Ag, which reduces the overall oxophilicity of the catalyst and prevents oxygen from incorporating into the metal lattice. The oxidation resistance of the catalyst has been reliably confirmed through robust experimental data, including in-situ Raman spectroscopy and quasi-in-situ XPS. The oxidation resistance mechanism has been further validated by DFT calculations. This novel design results in remarkable PEMWE performance (1 A cm-2@1.73V) and durability (< 1 μV degradation per hour). Additionally, the synthesis protocol is interesting and unconventional, to my knowledge, and seems potentially extendable to other alloy systems, including those with immiscible components. Therefore, I recommend its publication in Nature Communications after the authors address the following concerns.

Response: We sincerely thank the reviewer for their positive evaluation of our work and for recognizing the conceptual novelty of our AgRuIr alloy catalyst. We are particularly encouraged by the reviewer's appreciation of our strategy—utilizing a metallic catalyst for the acidic OER—as well as the demonstrated performance and durability within the PEMWE device. Each of the reviewer's insightful concerns has been carefully considered, and the manuscript has been revised accordingly to further strengthen the work. Our point-by-point responses to the specific technical concerns are provided below.

1. The vacancy-diffusion-assisted alloying mechanism is intriguing. To better understand this process, I suggest including a schematic diagram of the synthesis procedure in supporting information, showing how vacancy migration aids the alloying of immiscible elements.

Response: We thank the reviewer for this constructive suggestion. Accordingly, we have added a schematic illustration of the synthesis mechanism as **Fig. 2**a to visually demonstrate how vacancy migration facilitates the alloying of metals with intrinsic immiscibility. We have also expanded the discussion on **page 5** to more clearly describe this transformation from a core-shell precursor to a homogeneous alloy.

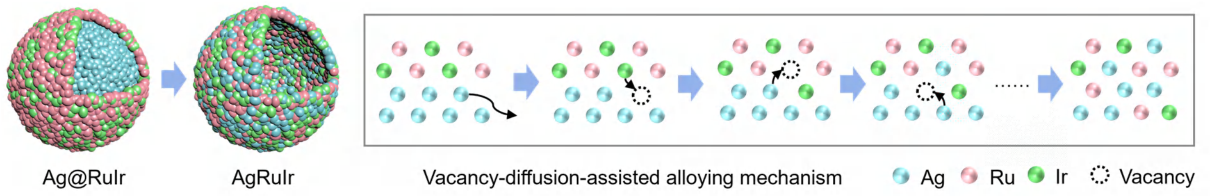

Fig. 2. (a) Schematic illustration of the vacancy-diffusion-assisted alloying mechanism.

Page 5: "We synthesized AgRuIr alloy nanocages via a vacancy-diffusion-assisted alloying mechanism (**Fig. 2**a). In this process, the diffusion of Ag vacancies—generated by HNO_3 etching—facilitates the full atomic mixing of the constituent metals. This mechanism effectively overcomes the intrinsic immiscibility of the elements, driving the transformation of the initial Ag@RuIr core–shell nanostructures into homogeneous AgRuIr alloy nanocages"

2. In the vacancy-diffusion-assisted alloying, the etching process is important because it directly correlates with the formation of vacancies. In this regard, the authors should include a discussion on how etching parameters, such as etchant concentration, etching time, and temperature, influence alloy formation.

Response: We thank the reviewer for this constructive suggestion. We agree that the etching parameters, including etchant concentration, etching time, and temperature, are critical to the formation of AgRuIr alloy nanocages via vacancy diffusion. In response, we have provided additional experimental results illustrating the influence of these parameters on the final nanostructure. These results are now presented in **Fig. S2** of the revised Supporting Information.

In general, too mild conditions lead to incomplete removal of the Ag cores from the Ag@RuIr core-shell nanospheres. Conversely, overly aggressive conditions result in excessive thinning of the shell, indicating a continuous and undesirable loss of Ag from the alloy framework. A detailed discussion of these effects has been incorporated into the revised manuscript.

Page 6: "In a typical procedure, the Ag@RuIr core-shell nanostructures were first prepared by the epitaxial growth of Ru and Ir on Ag nanospheres in a polyol system. These nanostructures were then subjected to an optimized etching process in 15 wt% HNO_3 at room temperature (25 °C) for 30 min, after which the resulting nanocages were collected by centrifugation and washed with deionized water. Control experiments (**Fig. S2**) demonstrated that under milder conditions (e.g., lower HNO_3 concentrations), the Ag cores cannot be effectively removed. Conversely, under more aggressive conditions (e.g., higher HNO_3 concentrations, elevated temperatures, or prolonged etching time), the

AgRuIr nanocages became excessively thin, suggesting a continuous loss of Ag from the alloy lattice. Consequently, the etching conditions were optimized to ensure the complete removal of the Ag cores for the formation of the AgRuIr alloy nanocages while preventing extensive dissolution of Ag. This approach allows for a wide composition window, enabling the optimization of catalytic performance.”

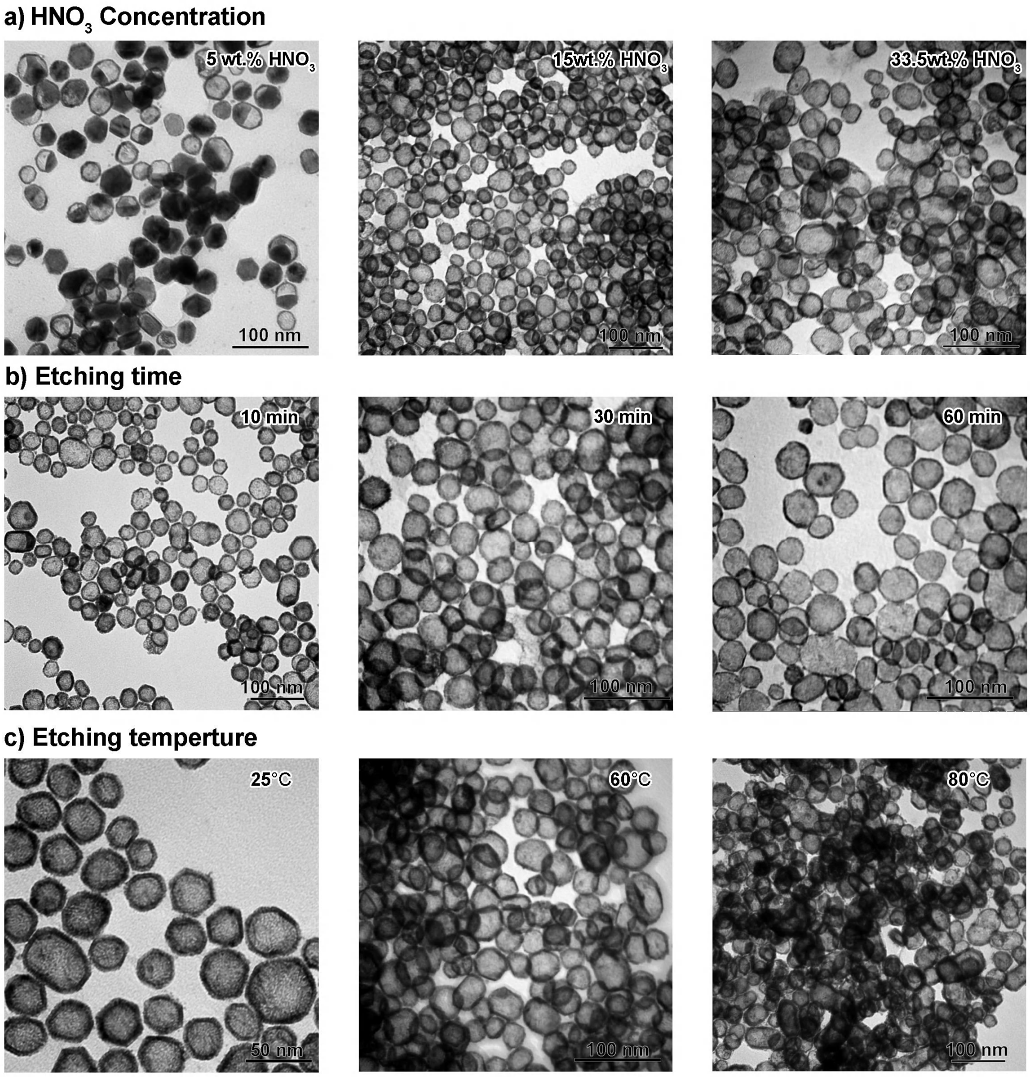

Fig. S2. Optimization of etching conditions. TEM images of products obtained by varying key etching parameters: (a) HNO_3 concentration, (b) etching duration, and (c) etching temperature. The images illustrate the structural evolution from incomplete core removal to excessive shell thinning.

3. Figure 2: While HAADF-STEM and phase analysis have revealed the distorted/disordered lattice arrangements in the metastable AgRuIr alloy nanocages, the authors further provided Ru K-edge EXAFS data to confirm the complex local atomic structure of the nanocrystals. I suggest that the authors include Ir L3-edge EXAFS data to better support the discussion on the local atomic structure.

Response: We thank the reviewer for this constructive suggestion. In accordance with this recommendation, we have acquired and included Ir L3-edge EXAFS data to further elucidate the local atomic structure of the AgRuIr alloy nanocages. These results have been incorporated into **Fig. S4** of the revised Supporting Information.

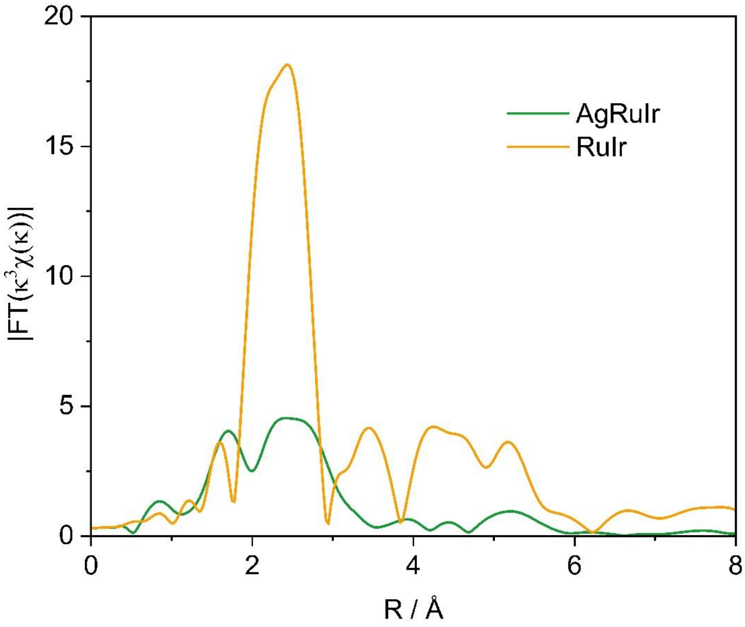

Fig. S4. Ir L_3-edge FT-EXAFS of the AgRuIr alloy nanocages and the RuIr alloy nanoparticles.

The Ir FT-EXAFS exhibits a broader distribution of Ir coordination distances in the AgRuIr alloy nanocrystals than in the RuIr alloy nanocrystals. This spectrum displays similar features to those observed in the Ru FT-EXAFS (**Fig. 2**k), with both confirming the presence of varied atomic distances and disordered atomic arrangements throughout the alloy sample.

4. Figure 4: The O1s and Ru 3d XPS spectra of AgRuIr after long-term OER testing were investigated to show that lattice oxygen and oxidized Ru were absent. How about the Ag-free, binary RuIr alloy? A similar study on RuIr alloy catalysts will be helpful in understanding the critical role of Ag in suppressing metal oxidation during catalysis.

Response: We thank the reviewer for the insightful comment. In **Fig. S33**, we provided the XPS spectra of the Ag-free $Ru_1Ir_{0.49}$ catalyst at various intervals during chronoamperometric OER testing at 1.6 V. Even at this relatively low potential (notably lower than the 1.74 V applied to the $Ag_{0.19}Ru_1Ir_{0.48}$ catalysts in **Fig. 4**i, j), both Ru and Ir underwent rapid oxidation. This is evidenced by the clear appearance of Ru^{6+} 3d peaks, Ir^{4+} 4f peaks, and O 1s peaks attributed to lattice oxygen. These results confirm the high susceptibility of the Ag-free RuIr catalyst to oxidation.

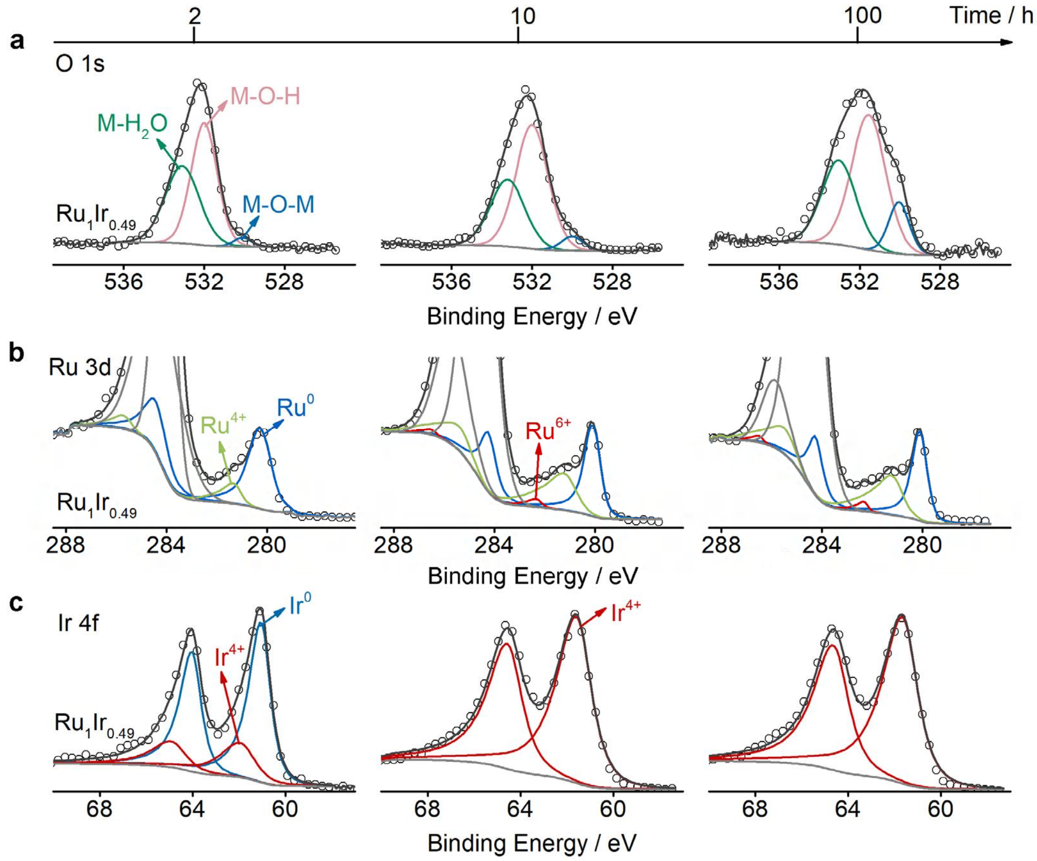

Fig. S33. XPS spectra of the $Ru_1Ir_{0.49}$ catalyst after chronoamperometric testing at 1.6 V for 2, 10, and 100 h. (a) O 1s. (b) Ru 3d. (c) Ir 4f.

To ensure a fair comparison at a potential identical to that used for the $Ag_{0.19}Ru_1Ir_{0.48}$ catalyst, we further investigated the Ru 3d and Ir 4f XPS of the Ag-free $Ru_1Ir_{0.49}$ catalyst after a short time (3 h) of chronoamperometric testing at 1.74 V. These results have been added to **Fig. S34** of the revised Supporting Information. Under these conditions, no Ru 3d or Ir 4f XPS signals were detectable, suggesting the complete loss of the RuIr catalyst due to severe oxidative dissolution.

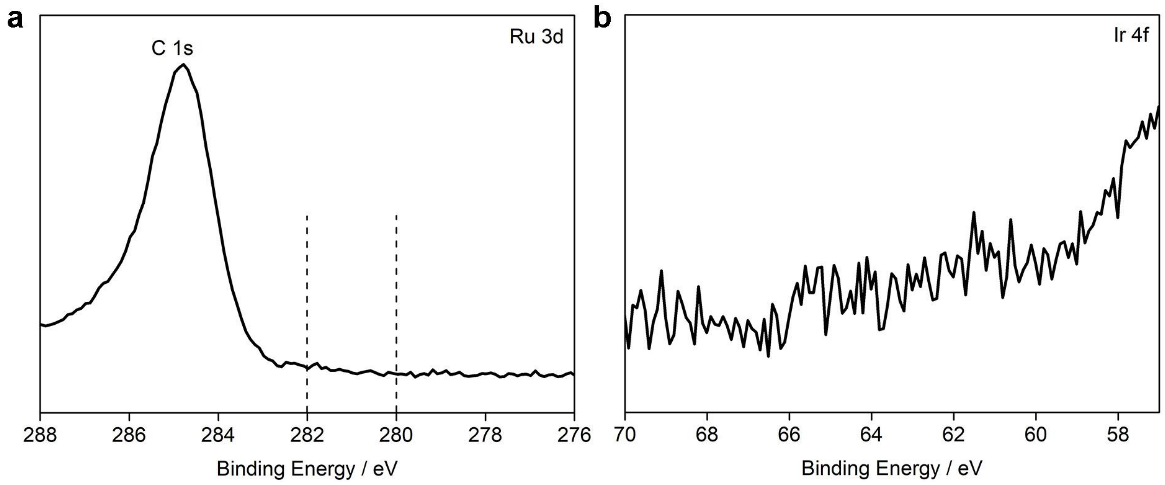

Fig. S34. XPS spectra of the $Ru_1Ir_{0.49}$ catalyst after chronoamperometric testing at 1.74 V for 3 h. (a) Ru 3d. The dotted lines indicate the expected positions for Ru 3d peaks. (b) Ir 4f. At this elevated potential, the Ag-free RuIr alloy catalysts underwent rapid degradation within 3 h, resulting in the

absence of detectable XPS signals.

These collective results confirm the critical role of Ag in suppressing metal oxidation in our AgRuIr alloy catalyst during OER catalysis.

5. In Figure 4b, the authors compared the metal dissolution levels of $Ag_{0.19}Ru_1Ir_{0.48}$ to those of many other catalysts, including binary $Ag_{0.2}Ru_1$ and $Ru_1Ir_{0.49}$. How about the metal dissolution levels for AgIr alloy catalysts?

Response: We thank the reviewer for the constructive suggestion. In response, we have evaluated the metal dissolution levels of binary $Ag_{0.22}Ir_1$ alloy nanocages to serve as an additional control for comparison with our ternary $Ag_{0.19}Ru_1Ir_{0.48}$ alloy catalyst. As the main text focuses primarily on Ru-based catalysts, these results have been incorporated into **Fig. S23** of the revised Supporting Information.

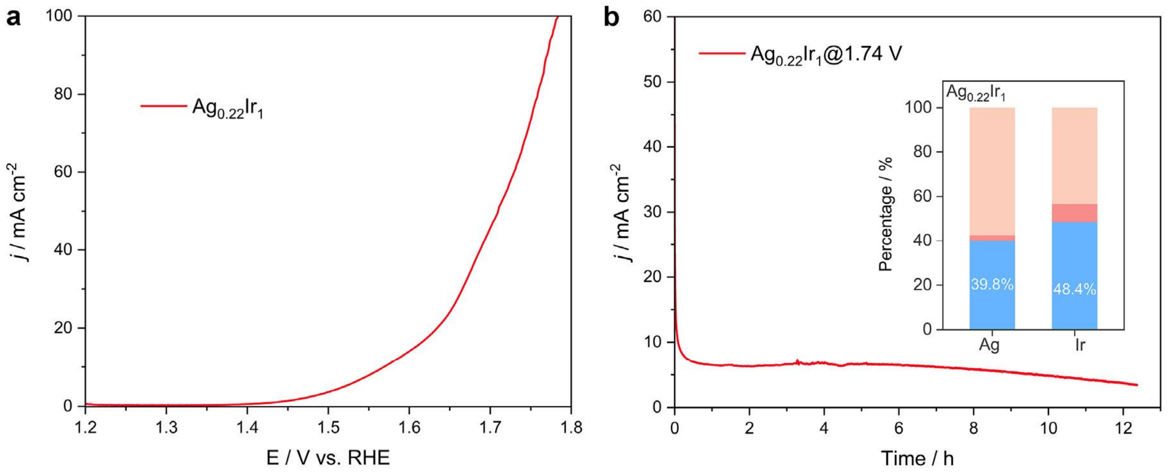

Fig. S23. Catalytic activity and stability of the AgIr alloy catalyst in acidic OER. (a) LSV curve in O_2-saturated 0.1 M $HClO_4$. (b) Chronoamperometric curve recorded at 1.74 V. Inset: Percentages of dissolved Ag and Ir following chronoamperometric testing, as determined by ICP-MS. The yellow, pink, and blue bars represent the percentages of metals dissolved in the electrolyte, redeposited on the counter electrode, and remaining undissolved, respectively.

As illustrated by these results, the binary AgIr alloy exhibits poor stability during the OER. A significant decline in current density was observed at 1.74 V within the first 12 h of testing. ICP-MS analysis revealed that over 60% of both Ag and Ir dissolved during this period, which accounts for the rapid degradation in catalytic performance. These results further highlight the superiority of the ternary AgRuIr alloy composition in achieving exceptionally low metal dissolution rates and robust OER stability.

6. $Ag_{0.06}Ru_1Ir_{0.47}$ is also an AgRuIr alloy catalyst. Why is its stability significantly lower than that of the $Ag_{0.19}Ru_1Ir_{0.48}$ counterpart? Please explain how such a minor difference in Ag content affects the stability of the catalyst.

Response: We thank the reviewer for this insightful question regarding the impact of Ag content on the stability of the catalysts. While both $Ag_{0.06}Ru_1Ir_{0.47}$ and $Ag_{0.19}Ru_1Ir_{0.48}$ are alloy catalysts, they exhibit distinct electrochemical behaviors that highlight the importance of optimal Ag composition.

The variation in OER stability suggests that the modulation of oxophilicity by Ag is highly composition-dependent. At a lower Ag content ($Ag_{0.06}Ru_1Ir_{0.47}$), the electronic modification is already present (resulting in enhanced OER activity and durability compared with Ag-free RuIr catalyst) but it is not fully optimized compared to the $Ag_{0.19}Ru_1Ir_{0.48}$ counterpart. Essentially, the $Ag_{0.06}Ru_1Ir_{0.47}$ sample lacks sufficient "protection" against the oxidative etching, leading to significantly lower durability. In contrast, the higher Ag fraction in the $Ag_{0.19}Ru_1Ir_{0.48}$ catalyst reaches a critical threshold necessary to effectively prevent lattice oxygen incorporation. This prevents the overoxidation of Ru and Ir into soluble high-valent species. These findings demonstrate that a specific amount of Ag is required to achieve the oxidation resistance necessary for sustained electrolysis.

We have added a clarifying discussion regarding these composition-dependent effects to the revised manuscript:

Page 15: "These findings indicate that the corrosion behavior and catalytic stability of the AgRuIr alloy catalyst are highly sensitive to the Ag content. Exceptional corrosion resistance is only realized when a sufficiently high fraction of Ag is incorporated into the alloy lattice. Collectively, these results highlight the critical role of Ag in mitigating oxidative dissolution and enhancing the overall durability of RuIr-based metallic catalysts under acidic OER conditions."

7. In PEMWE testing, Figure 6b shows that the cell voltage was 1.73 V to achieve the current density of 1 A cm^{-2}, while Figure 6c indicates that the cell voltage was approximately 2 V to achieve the same current density. What is the reason for the inconsistency in the cell voltage? A more detailed discussion on this data is needed to avoid confusion

Response: We thank the reviewer for pointing out the difference in cell voltages between **Fig. 6**b and **Fig. 6**c. We agree that additional clarification is necessary to distinguish the testing conditions underlying the two datasets. The difference between the cell voltage observed in the polarization test (1.73 V at 1 A cm^{-2}) and that in the chronopotentiometric stability test (~2.0 V at 1 A cm^{-2}) arises from the following factors:

(1) Transient vs. steady-state kinetics: The *I–V* polarization curve reflects a transient, non-steady-state process. During a continuous potential scan, the coverage of oxygen-evolution intermediates on the catalyst surface evolves dynamically. This results in a lower instantaneous polarization resistance than that observed in steady-state chronopotentiometric testing, where the surface intermediate coverage reaches a stable, high-occupancy state.

(2) Mass transport effects: In steady-state chronopotentiometric testing, the intrinsic catalyst activity is coupled with complex mass transport dynamics within the PEMWE device. Over extended durations, factors such as oxygen bubble accumulation and local reactant diffusion limitations become more pronounced, leading to higher overpotentials than the values captured during a rapid *I–V* scan.

(3) Resistance difference: The *I–V* data were recorded using a Gamry electrochemical workstation with 90% *iR* compensation, while the long-term chronopotentiometric stability test was conducted on a Neware battery testing system without *iR* compensation. The differences in inherent internal resistance and *iR* compensation levels contribute to the different cell voltages.

A clarifying statement has been added to the revised manuscript:

Page 19: "The cell voltage in this steady-state test (~2.0 V) is slightly higher than that observed in the transient polarization testing (1.73 V), which may arise from differences in surface intermediate coverage, time-dependent mass-transport phenomena (such as bubble accumulation), and variations in the uncompensated resistance of the distinct measurement systems employed."

8. The EIS data in Figure 3e were fitted by an equivalent circuit shown in Table S4. The reason for choosing this model should be added to the text. Besides, the physical meanings of R_s, CPE, and R_{ct} should be clearly explained and discussed.

Response: We thank the reviewer for the constructive suggestions. The Nyquist plots in **Fig. 3**e are each characterized by a single semi-circular feature, suggesting a typical electrochemical double-layer structure. Consequently, a standard R_s (R_{ct} CPE) equivalent circuit model was employed to fit these data. In this model, R_s represents the series solution resistance (or ohmic resistance), R_{ct} is the charge-transfer resistance (inversely correlates with the OER activity), and CPE denotes the constant phase element (commonly used to model the non-ideal capacitive behavior of an electrochemical interface, often arising from surface roughness or the heterogeneous distribution of active sites on the catalyst surface).

The corresponding discussion and detailed explanations of these parameters have been added to **Table S4** of the revised Supporting Information.

Response to Reviewer #3:

Reviewer's comments: This manuscript reports the synthesis of metastable $Ag_{0.19}Ru_1Ir_{0.48}$ alloy nanocages that the authors claim remain metallic in the bulk under harsh acidic OER conditions, thereby reducing oxophilicity and suppressing over-oxidation. The catalyst delivers high intrinsic activity (e.g., 200 mA cm^{-2} at 1.463 V vs RHE) and notable durability, including PEMWE operation

at 1 A cm^{-2} and 1.73 V with minimal degradation (0.93 μV h^{-1} over 1500 h) and ultralow dissolution (0.5–0.7 % k h^{-1}). The nanocage synthesis route and the strategy to alloy otherwise immiscible components are innovative. Overall, the performance metrics and especially the long-term durability are impressive and, if validated, would represent a meaningful advance for acid OER and PEMWE.

Response: We thank the reviewer for their positive assessment of our work. We are pleased that the design of our metallic $Ag_{0.19}Ru_1Ir_{0.48}$ alloy nanocages to suppress overoxidation was found to be a meaningful advance for acidic OER and PEMWE. We appreciate the recognition of our innovative alloying route for immiscible components and the impressive durability metrics. We have carefully addressed each of the reviewer's comments and technical suggestions to further validate and strengthen the manuscript. Our point-by-point responses are provided below.

1. The manuscript's central hypothesis that the $Ag_{0.19}Ru_1Ir_{0.48}$ nanocages maintain a metallic bulk state throughout OER operation avoiding overoxidation is not fully supported by the presented XPS data. Figure S18 shows that the Ir 4f spectrum after 100 h at 1.74 V corresponds with an asymmetric line shape typical of IrO_2 (https://doi.org/10.1002/sia.7215) rather than metallic Ir. This suggests significant surface oxidation or complete oxidation of Ir (considering shell thickness of 3.4 nm), contradicting the claim of persistent metallic character. Fig S22 also confirms this trend for both Ru and Ir after CA at 200 mA/cm^2. I also suggest to add to Figure 4 Ir 4f and Ag Auger spectra at 2 h, 10 h, and 100 h after CA at 1.74V, and add a table in the SI summarizing binding energies and relative concentrations for O, Ru, Ir, and Ag.

Response: We thank the reviewer for the insightful analysis of the XPS data. We acknowledge that the emergent asymmetric line shapes and binding energy shifts in the Ir 4f spectra—observed after 100 h of operation at 1.74 V—indicate the formation of surface oxides. We agree that "persistent metallic character" may lack the necessary precision to describe these findings. Consequently, we use the term of "retention of the bulk metallic state" throughout the revised manuscript. This terminology more accurately accounts for the evolution of the oxygenated metal surface and the development of a thin oxide layer during extended durability testing. Example of the discussion:

Page 4: "As a result, the AgRuIr alloy nanocrystals form only an oxygenated surface layer or an ultrathin oxide layer after extended durations, while maintaining a metallic bulk, even though none of their individual components could withstand the elevated potential alone. The suppression of oxygen incorporation into the metal lattice is confirmed by X-ray photoelectron spectroscopy (XPS) and in situ Raman analyses."

Our point-by-point responses are as follows:

(1) Surface vs. bulk oxidation: Our XPS results (**Fig. S27**, Ir 4f XPS at 1.74 V for 2 h and 10 h; **Fig. 4**j, O XPS, Ru 3d XPS at 1.74 V for 100 h) confirm that during initial and intermediate stages of

catalysis, both Ru and Ir remain in their metallic states. After extended durations, metals on the surface might have partially oxidized, with Ir^{4+} appearing after 100 h at 1.74 V (**Fig. S27**) and Ru^{4+} appearing after 1000 h at 200 mA cm^{-2} (**Fig. S31**). Ir^{4+} forms before Ru^{4+}, which might because Ir is partially enriched on the surface (**Fig. 2**c), leading to easier oxidation during catalysis and facile detection by XPS, a surface-sensitive analysis technique.

While XPS as a surface-sensitive technique detects a thin oxide layer at the surface after extended catalysis, bulk-sensitive techniques confirm the persistence of the metallic framework.

(i) Raman spectroscopy: As a relatively bulk-sensitive technique for these nanocrystals, Raman spectroscopy (**Fig. S28**) shows no characteristic peaks for rutile-type or amorphous metal oxides even after 100 h of operation at 200 mA cm^{-2}.

(ii) HAADF-STEM imaging: We have provided high-resolution STEM images (**Fig. 4**k and **Fig. S30**) that clearly resolve continuous metallic lattice fringes throughout the nanocage after 200 h of testing at 200 mA cm^{-2}, confirming that the structural skeleton remains an alloy.

(iii) Ru FT-EXAFS: To provide even deeper structural insight, we have included Ru FT-EXAFS data of the AgRuIr alloy nanocages after catalysis (**Fig. S32**). The Fourier-transformed spectra of the post-OER samples are nearly identical to the pristine metallic nanocages, providing definitive bulk-sensitive evidence that the Ru coordination environment remains metallic.

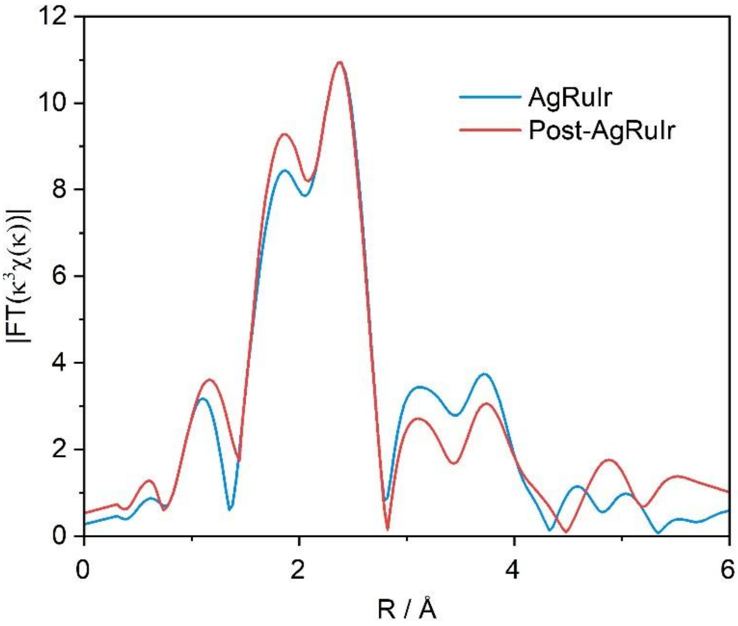

Fig. S32. Ru FT-EXAFS of the $Ag_{0.19}Ru_1Ir_{0.48}$ alloy nanocages before and after OER catalysis. As a bulk-sensitive technique, X-ray absorption spectroscopy confirms the retention of the bulk metallic state in the $Ag_{0.19}Ru_1Ir_{0.48}$ alloy nanocages during the OER process.

These results collectively prove that while an ultrathin surface oxide may eventually form, the bulk of the 3.8 nm nanocages retains their metallic alloy integrity.

(2) Retarded Oxidation Kinetics and the Role of Ag. The transition to an oxide phase is remarkably slow compared to Ag-deficient or Ag-free counterparts. Our analysis suggests that oxygen only incorporates into the Ir surface lattice after 100 h at 1.74 V and into the Ru surface lattice after 1000 h at 200 mA cm^{-2}. Even though, metallic Ru still dominates in the nanocages. For comparison, both the Ag-deficient $Ag_{0.06}Ru_1Ir_{0.47}$ and the Ag-free $Ru_1Ir_{0.49}$ catalysts exhibited lattice oxygen signals in the O 1s XPS within 2 h of chronoamperometric testing at an even lower potential of 1.6 V, with the lattice oxygen content increasing over time, suggesting continuous metal oxidation (**Fig. S33, S35**, and **Table S11–S13**). This highlights the critical role of Ag in reducing the oxophilicity of the alloy, which is essential for achieving exceptional OER durability.

(3) Nature of the Active Sites. Our data confirm that during the initial and intermediate stages of catalysis (e.g., at 1.74 V for 2 h and 10 h), both Ru and Ir remain in their metallic states (**Fig. S26** and **Fig. 4**j). Despite the absence of detectable oxides at these stages, the catalyst already exhibits exceptional OER activity and stability. This confirms that the exceptional OER performance originates from an oxygenated metal surface rather than a bulk oxide phase.

As requested, we have presented Ir 4f and Ag Auger spectra at 2 h, 10 h, and 100 h after CA at 1.74V in **Fig. S26** and **Fig. S27** of the revised Supporting Information. Additionally, we have provided **Tables S11–S13** in the Supporting Information, summarizing the binding energies and relative atomic concentrations for O, Ru, Ir, and Ag.

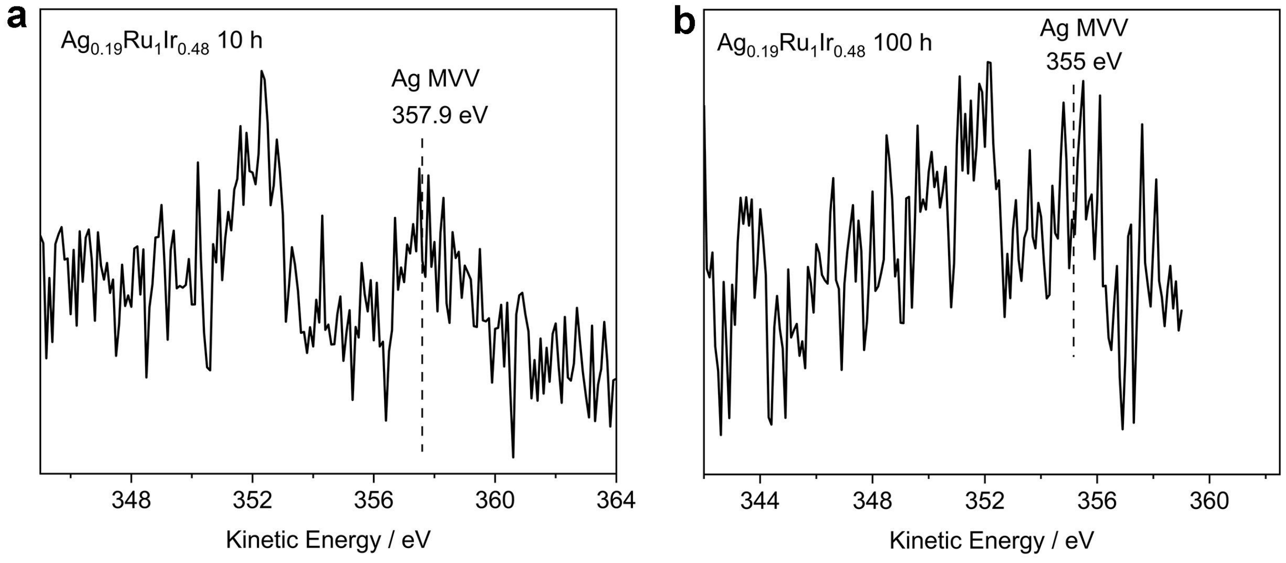

Fig. S27. Auger electron spectroscopy of Ag in $Ag_{0.19}Ru_1Ir_{0.48}$ alloy nanocages sampled at different times in chronoamperometric testing at 1.74 V. (a) 10 h. (b) 100 h. The Ag MVV peaks are observed at kinetic energies of 355–358 eV, corresponding to Ag^0.

Table S7. O 1s XPS fitting results of $Ag_{0.19}Ru_1Ir_{0.48}$ after chronoamperometric testing at 1.74 V for different times. (spectra, Fig. 4i). The table shows peak positions for each O species in the catalysts. The values in parentheses are percentages of the specific O species among all O species in the catalyst, excluding physisorbed H_2O.

$Ag_{0.19}Ru_1Ir_{0.48}$	M-H_2O/eV	M-OH/eV	M-O-M/eV
2 h	532.9	532	–
10 h	533.2	532	–
100 h	533.2	532.1 (91.33%)	530.1 (8.67 %)

Table S8. Ru 3d XPS fitting results of $Ag_{0.19}Ru_1Ir_{0.48}$ after chronoamperometric testing at 1.74 V for different times (spectra, Fig. 4j).

$Ag_{0.19}Ru_1Ir_{0.48}$	Ru^0/eV	$Ru^{3+/4+}$/eV	Ru^{6+}/eV
2 h	280	–	–
10 h	280	–	–
100 h	280.2 (80.8%)	281.2 (19.2%)	–

Table S9. Ir 4f XPS fitting results of $Ag_{0.19}Ru_1Ir_{0.48}$ after chronoamperometric testing at 1.74 V for different times (spectra, Fig. S26).

$Ag_{0.19}Ru_1Ir_{0.48}$	Ir^0/eV	Ir^{4+}/eV	Ir^{6+}/eV
2 h	60.7	–	–
10 h	60.4	–	–
100 h	–	61.5	–

Table S10. Ag Auger electron spectrum fitting results of $Ag_{0.19}Ru_1Ir_{0.48}$ after chronoamperometric testing at 1.74 V for different times (spectra, Fig. S14 and S27).

$Ag_{0.19}Ru_1Ir_{0.48}$	Ag^0/eV	Ag^+/eV
2 h	358.2	–
10 h	357.9	–
100 h	–	355

2. It is undeniable that the results for $Ag_{0.19}Ru_1Ir_{0.48}$ nanocages in terms of OER activity and stability are very good, and considering the no preservation of metallic bulk from XPS data, the authors should consider that high activity and stability could be related to other mechanism rather than to metallic preservation. Recent work (Nature Communications 2025, DOI: 10.1038/s41467-025-55910-1) shows that Ru–Ir atomic mixing in rutile-type oxides can deliver similar performance via optimized oxygen adsorption. The authors should consider this alternative mechanism since the PEMWE activity results shown in this article are very similar: 3 A/cm² @ 2 V vs. 3.17 A/cm² from the previously cited Nat Comm 2025.

Response: We thank the reviewer for these constructive comments and for directing our attention to the recent work in *Nature Communications* (2025). We agree that atomic mixing is a paramount factor in achieving high structural stability and catalytic durability. However, we believe there is a fundamental mechanistic distinction between the rutile oxide system and our AgRuIr alloy nanocages.

As discussed in our response to Point 1, our catalyst exhibits exceptional OER activity during the initial 2–10 hours of operation—a period during which XPS, Raman, and TEM/STEM confirm the absence of bulk oxides. This indicates that the metallic alloy framework, rather than a developed oxide lattice, serves as the primary active phase for the exceptional OER performance observed.

While the PEMWE performance metrics (current density at 2 V) are indeed comparable to the cited rutile-type oxide, the underlying stabilization mechanisms differ significantly. In the rutile oxide system, Ru–Ir mixing serves to stabilize a pre-existing oxide lattice against further degradation. In contrast, the stability of our catalyst is rooted in the intrinsic oxidation resistance of a unique metastable alloy phase. This metallic state effectively suppresses the transition into a metal oxide phase, fundamentally preventing the over-oxidation.

We agree that atomic mixing is a critical factor for stability, albeit through a different path in our system. The comprehensive atomic mixing of Ag, Ru, and Ir—facilitated by our unique vacancy-diffusion-assisted alloying mechanism—maximizes inter-component electronic interactions. This ensures homogeneous physicochemical properties throughout the nanocrystal, preventing localized leaching and contributing to enhanced OER durability. We have incorporated this discussion into the revised manuscript to acknowledge these alternative mechanisms and clarify our findings:

Page 6: "The atomic mixing of the alloy components, facilitated by the unique vacancy-diffusion-assisted alloying mechanism, maximizes inter-component electronic interactions and ensures homogeneous physicochemical properties throughout the nanocages, thereby enhancing catalytic performance (a similar case in RuIr oxide system, see ref. 48)."

[48] Park, Y. *et al.* Atomic-level Ru-Ir mixing in rutile-type $(RuIr)O_2$ for efficient and durable oxygen evolution catalysis. *Nat. Commun.* **16**, 579 (2025).

3. The authors use carbon cloth for OER electrochemical tests at potentials up to 1.74 V vs RHE and high current densities (200 mA/cm^2). Carbon substrates are not electrochemically inert under such acid OER conditions, they can oxidize, and contribute with parasitic currents, thereby confounding activity/stability attribution to the catalyst. I strongly recommend repeating key measurements on a corrosion-resistant substrate, such as Ti mesh, and/or provide blank carbon-cloth controls at 1.74 V to quantify substrate contributions.

Response: We thank the reviewer for these insightful comments regarding the stability of carbon-based substrates under harsh acidic OER conditions. To rigorously exclude any parasitic current contributions from the carbon cloth, we conducted the requested control experiments.

First, a blank carbon cloth was evaluated as the working electrode. LSV testing in O_2-saturated 0.1 M $HClO_4$ revealed a negligible current density (< 1 mA cm^{-2}) within the potential range of 1.2–1.8 V. Furthermore, chronoamperometric testing at 1.74 V showed that the current density dropped from an initial ~0.3 mA cm^{-2} to less than 0.012 mA cm^{-2} after only 0.5 h. These results, now provided in **Fig. S24**, confirm that the substrate contribution to the overall catalytic activity is negligible.

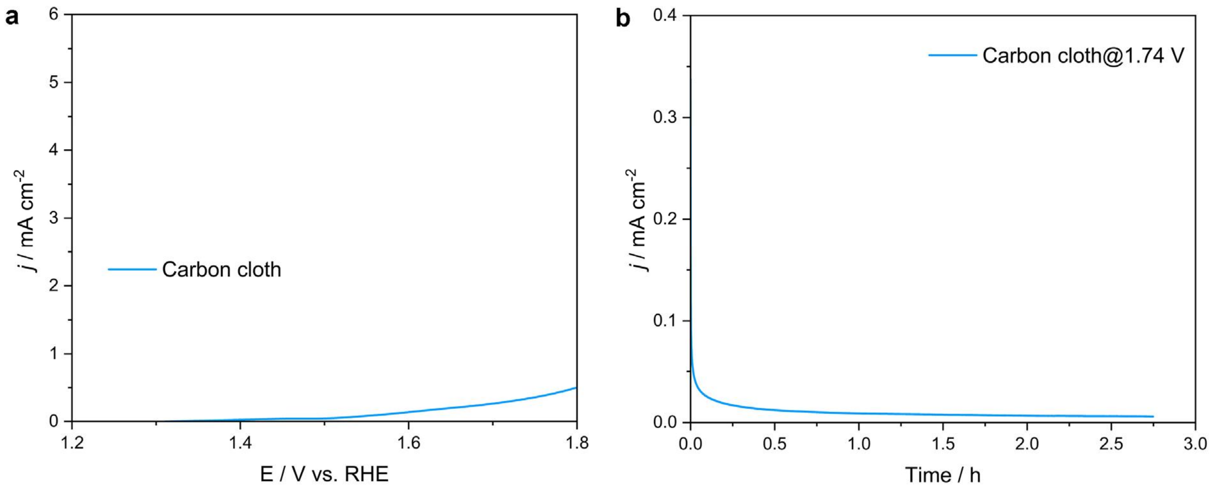

Fig. S24. Electrochemical OER activity of the carbon cloth substrate. (a) LSV curve in O_2-saturated 0.1 M $HClO_4$ with 95% *iR* compensation. (b) Chronoamperometric curve recorded at 1.74 V. The minimal current response confirms the negligible contribution of the substrate to the measured catalytic activity.

Second, we repeated the stability measurements using titanium (Ti) fiber felt as a corrosion-resistant alternative substrate. The chronoamperometric curve for the $Ag_{0.19}Ru_1Ir_{0.48}$ alloy nanocages on Ti felt at 1.74 V (loading, ~0.5 mg cm^{-2}) yielded a stable current density of ~213.7 mA cm^{-2} over 100 h of testing. This is highly consistent with the results obtained on carbon cloth (~215.5 mA cm^{-2}, **Fig. 4b**), again confirming that the observed performance is intrinsic to the AgRuIr catalyst and not a result of substrate oxidation. These data have been added to **Fig. S25** of the revised Supporting Information.

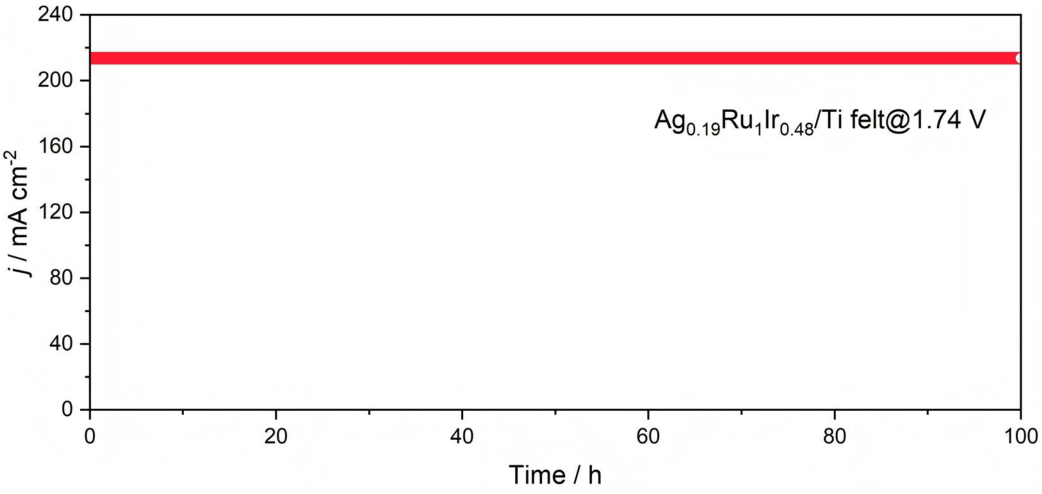

Fig. S25. Electrocatalytic OER performance of the $Ag_{0.19}Ru_1Ir_{0.48}$ alloy nanocages on a titanium fiber felt substrate. Chronoamperometric curve recorded at 1.74 V with a catalyst loading of ~0.5 mg cm^{-2}. The stable current density of ~213.7 mA cm^{-2} over 100 h of testing matches the performance on carbon cloth (215.5 mA cm^{-2}), validating the substrate-independent nature of the catalyst's durability.

A clarification has been made in the revised manuscript:
Page 15: "Notably, control experiments confirmed that the carbon cloth substrate provided negligible contribution to the observed OER current densities (**Fig. S24** and **S25**)."

4. The PEMWE results substantially strengthen the manuscript by demonstrating that the exceptional half-cell stability of $Ag_{0.19}Ru_1Ir_{0.48}$ does translate into record-level single-cell durability at 1 A cm^{-2}, supported by impressively low dissolution rates. Nevertheless, the claims of "practical applicability" would be more convincing if tempered by a clearer discussion of operating conditions, catalyst loading targets, and durability under dynamic operation. Rather than definitively establishing AgRuIr as a drop-in industrial anode catalyst, these results should be viewed as a compelling proof of concept for a new stabilization strategy for Ru-based PEMWE anodes, warranting further validation under more demanding, application-relevant conditions. To further validate practical PEMWE durability, the catalyst should be evaluated under dynamic operation protocols that mimic renewable-driven electrolysis, including start–stop cycling (e.g., alternating 10–20 min operation at 1 A cm^{-2} with 10 min at open-circuit or load cycling between low and high current densities as corrosion stress tests, followed by recovery under steady operation. Also, monitoring voltage drift, high-frequency resistance (HFR), together with post-mortem MEA analysis (SEM, TEM, XPS) would directly demonstrate whether the exceptional stability persists under realistic, non-steady-state PEMWE conditions.

Response: We sincerely thank the reviewer for the constructive assessment of our single-cell performance and for the insightful suggestion to evaluate the catalyst under dynamic operation protocols. We agree that demonstrating stability under non-steady-state conditions, such as renewable-driven load fluctuations, is essential for validating the practical potential of our stabilization strategy. We admit that these results should be viewed as a compelling proof of concept for a new stabilization strategy for Ru-based PEMWE anodes, and we acknowledge that full-scale practical applicability

warrants further exploration in the future.

To address the reviewer's specific suggestions, we implemented a start-stop cycling protocol alternating between 1-hour operation at 1 A cm^{-2} and 1-hour periods at OCP, totaling a 10-hour stress test. The results demonstrate the exceptional robustness of the $Ag_{0.19}Ru_{1}Ir_{0.48}$ catalyst:

(1) The cell voltage remained highly stable across cycles, and real-time monitoring of the high-frequency resistance (HFR) showed minimal fluctuations, indicating that the MEA maintained excellent mechanical and electrical contact during dynamic operation.

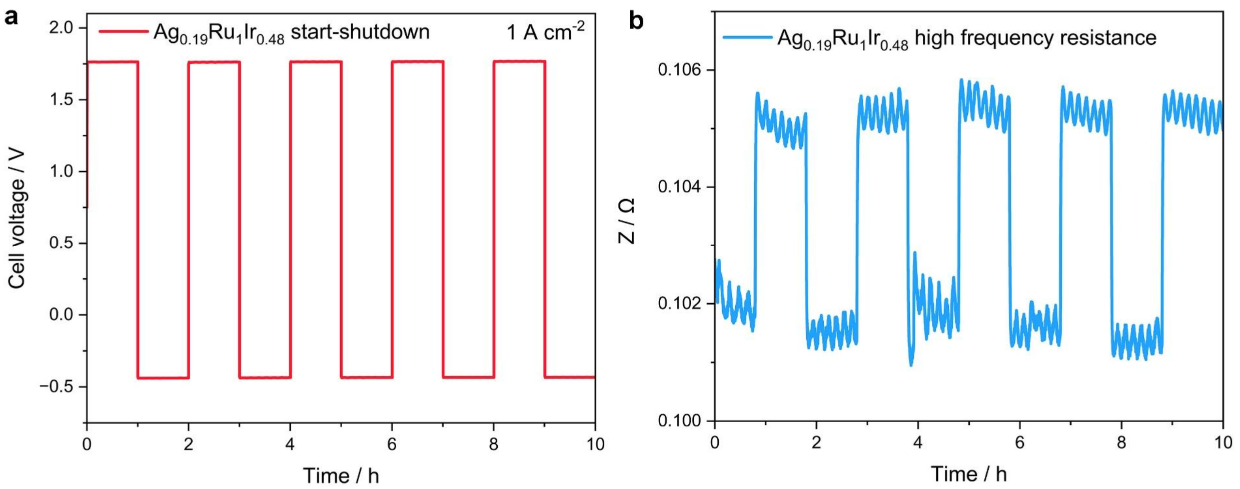

Fig. S49. Dynamic start-stop cycling performance of $Ag_{0.19}Ru_{1}Ir_{0.48}$ alloy nanocages in a PEMWE single cell. (a) Cell voltage profile during start-stop cycles (1 h at 1 A cm^{-2} followed by 1 h at OCP). (b) Real-time monitoring of high-frequency resistance (HFR) during the cycling test, demonstrating the structural and electrical stability of the MEA.

(2) TEM and HRTEM imaging of the catalyst harvested from the MEA after the start-stop test confirmed that the hollow nanocage morphology and metallic lattice fringes were well preserved. EDS mapping further verified that Ag, Ru, and Ir remained homogeneously mixed with no evidence of phase segregation or structural collapse.

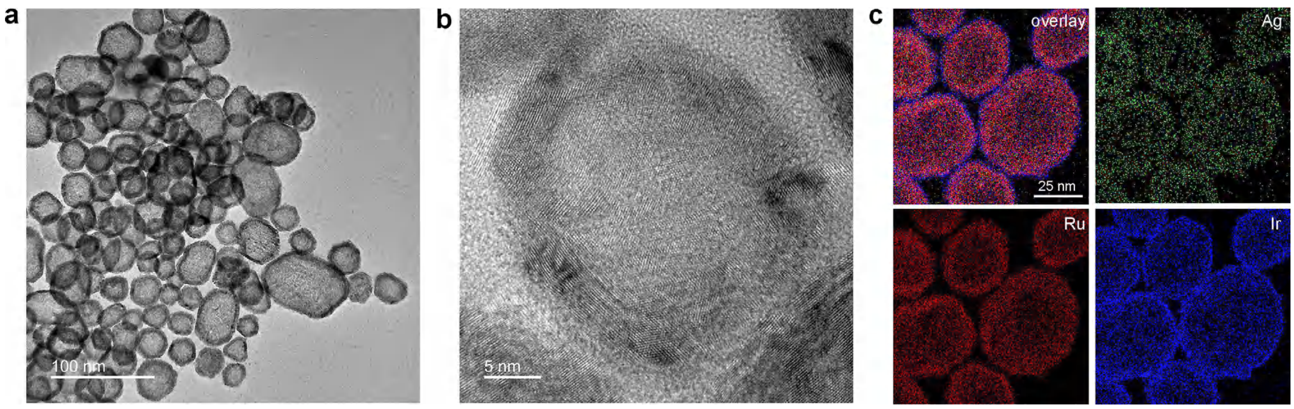

Fig. S50. Structural and compositional characterization of $Ag_{0.19}Ru_{1}Ir_{0.48}$ alloy nanocages after the start-stop testing. (a) TEM image showing the preservation of the hollow nanocage morphology. (b)

HRTEM image resolving metallic lattice fringes, confirming the stability of the alloy framework. (c) EDS mapping showing the homogeneous distribution of Ag, Ru, and Ir after dynamic operation.

(3) XPS analysis revealed that the Ru and Ir components remained predominantly in their metallic states. The lattice oxygen (M-O-M) content was determined to be only 9.62%, with Ru^{4+} and Ir^{4+} percentages at 2.78% and 7.03%, respectively, demonstrating significantly suppressed oxidation under dynamic conditions.

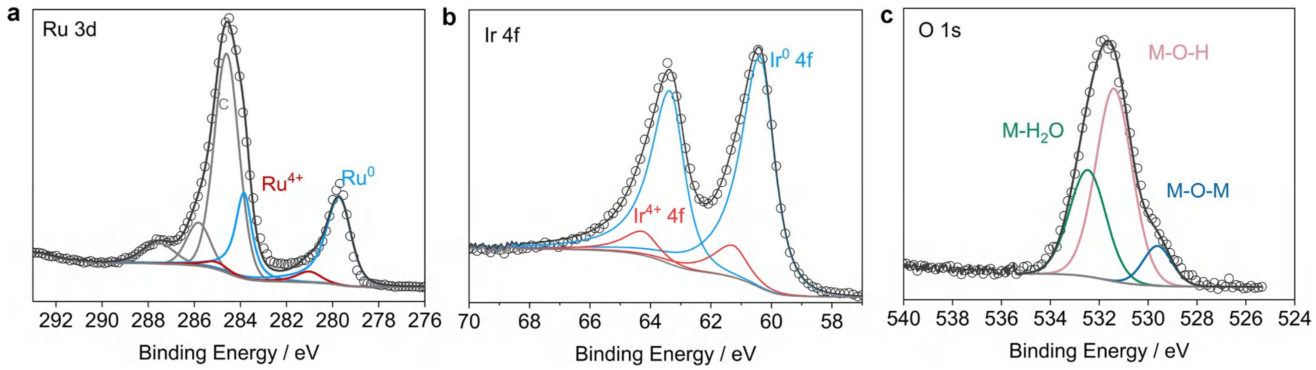

Fig. S51. XPS analysis of the $Ag_{0.19}Ru_1Ir_{0.48}$ alloy nanocages harvested from the MEA after the start-stop testing. (a) Ru 3d. (b) Ir 4f. (c) O 1s. The spectra confirm the dominant metallic state and suppressed oxidation of the catalyst under non-steady-state conditions.

Table S20. Summary of the relative percentages of lattice oxygen (M-O-M), oxidized Ru (Ru^{4+}), and oxidized Ir (Ir^{4+}) derived from XPS after the start-stop testing.

$Ag_{0.19}Ru_1Ir_{0.48}$	M-O-M/%	Ru^{4+}/%	Ir^{4+}/%
After start-stop cycles	9.6	2.8	7.0

These findings, now included in the revised manuscript (copied below) and Supporting Information (**Fig. S49–S51, Table S20**), suggest that the $Ag_{0.19}Ru_1Ir_{0.48}$ alloy nanocages possess significantly enhanced oxidation resistance even under demanding, non-steady-state PEMWE conditions.

Page 21: "To evaluate practical potential, the $Ag_{0.19}Ru_1Ir_{0.48}$ alloy nanocages were subjected to a dynamic start-stop protocol in a PEMWE single cell, alternating between 1 A cm^{-2} and open-circuit potential to mimic renewable energy fluctuations (**Fig. S49-S51**, **Table S20**). The catalyst demonstrated exceptional resilience, maintaining stable cell voltage and high-frequency resistance, which confirms the structural and electrical integrity of the MEA. Post-cycling analysis via TEM and EDS mapping verified that the hollow morphology and atomic mixing were fully preserved, while XPS showed that the metals remained predominantly metallic with a low lattice oxygen content of 9.6%. These results provide a compelling proof of concept for an oxidation-resistant metallic catalyst for the OER capable of withstanding non-steady-state operational stress."

5. Provide mass-activity normalization (A mg^{-1}(Ru+Ir)) at 2.0 V for PEMWE tests. Benchmark against the (RuIr)O_2 Nature Communications study (DOI: 10.1038/s41467-025-55910-1): ≈3.17 A cm^{-2} at 2.0 V, mass activity ≈19.84 A mg^{-1}(Ru+Ir) at 2.0V.

Response: We thank the reviewer for the helpful suggestion to include mass-activity normalization for a more comprehensive benchmarking of our PEMWE results. Following this recommendation, we conducted PEMWE testing at 80 °C to match the operating conditions reported in the *Nature Communications* study. At 2.0 V, our $Ag_{0.19}Ru_{1}Ir_{0.48}$ catalyst achieved a current density of 4.99 A cm^{-2} and mass activity of 5.67 A mg_{Ru+Ir}^{-1}, demonstrating high PEMWE performance. We have added these comparison data to the revised Supporting Information.

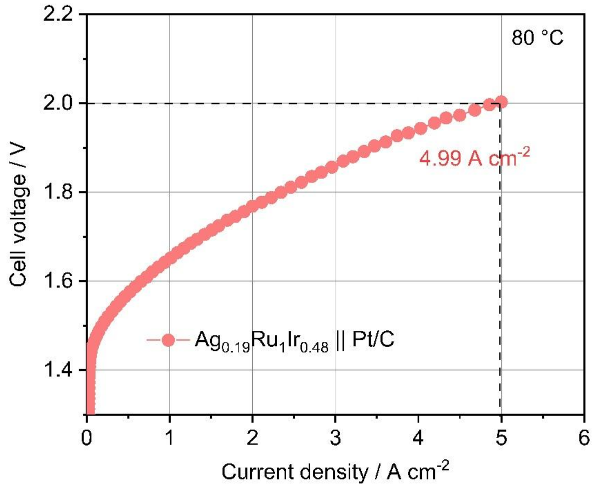

Fig. S48. PEMWE performance at 80 °C. Polarization curve using $Ag_{0.19}Ru_{1}Ir_{0.48}$ as the anode catalyst and commercial Pt/C as the cathode catalyst. Testing was performed at 80 °C without *iR* compensation.

Table S16. Comparison of current density and mass activity at 2.0 V PEMWE with different anode catalysts operated at 80 °C.

Catalyst	Current density at 2 V (A cm^{-2})	Mass activity at 2 V (A mg_{Ru+Ir}^{-1})	Reference
$Ag_{0.19}Ru_{1}Ir_{0.48}$	4.99	5.67	This work
$(RuIr)O_2/C$	4.96	19.84	37
Com.IrO_2	3.17	12.68	

6. I think the work on optimizing the catalyst preparation and composition in terms of the Ru/Ir ratio and shell thickness is very good and deserves to be detailed in the main text, with reference to Figs. S4–S8.

Response: We thank the reviewer for this constructive suggestion. We agree that the optimization of the shell thickness and the Ir/Ru atomic ratio is fundamental to the high performance of our catalyst. In accordance with this recommendation, we have added the detailed discussion of these parameters in the revised manuscript (copied below). This addition provides a clearer rationale for our selection of the $Ag_{0.19}Ru_{1}Ir_{0.48}$ composition with a 3.8 nm shell thickness as the primary catalyst.

Page 9: "The metastable $Ag_{0.19}Ru_1Ir_{0.48}$ alloy nanocages demonstrate exceptional catalytic activity for the acidic OER (**Fig. 3**a–e), outperforming Ag-deficient $Ag_{0.06}Ru_1Ir_{0.47}$ alloy nanocages (**Fig. S7**), $Ru_1Ir_{0.49}$ alloy nanocrystals (**Fig. S8**), and well-established commercial RuO_2 and IrO_2 benchmarks (**Fig. S8**). Optimization of the shell thickness (≈ 3.8 nm) and the Ir/Ru ratio (≈ 0.5) is critical to ensuring the optimal OER performance. Specifically, a sufficient shell thickness is required to maintain a robust nanocage architecture throughout the catalytic process. A control sample with a thinner shell (≈1.6 nm) exhibited rapidly degrading catalytic activity within 30 h during chronopotentiometric testing at 50 mA cm^{-2} (**Fig. S9** and **S10**). Additionally, the Ir/Ru ratio within the AgRuIr nanocages significantly influences their OER activity. Control experiments revealed a volcano-shaped dependence of OER activity on the Ir/Ru ratio, with maximum activity achieved at Ir/Ru ≈ 0.5 (**Fig. S11–S13**). This trend can be attributed to the electronic interactions between Ru and Ir, which effectively modulate the adsorption energies of oxygenated intermediates and optimize the overall OER kinetics. Consequently, $Ag_{0.19}Ru_1Ir_{0.48}$ alloy nanocages with an average shell thickness of 3.8 nm were selected to demonstrate the superior OER performance of this AgRuIr catalyst system."

Response to Reviewer #4:

Reviewer's comments: I co-reviewed this manuscript with one of the reviewers who provided the listed reports. This is part of the Nature Communications initiative to facilitate training in peer review and to provide appropriate recognition for Early Career Researchers who co-review manuscripts.

Response: We thank both the primary reviewer and the co-reviewer for their time and careful evaluation of our manuscript. We are pleased to participate in this initiative and appreciate the constructive feedback provided through this collaborative effort, which has helped us further improve the quality of our work.

Response to Reviewer #5:

Reviewer's comments: The manuscript titled "Oxidation-Resistant AgRuIr Alloy Nanocages for Efficient and Enduring Oxygen Evolution in PEM Water Electrolysis" reports the development of metastable AgRuIr alloy nanocages with high intrinsic OER activity and exceptional long-term stability under acidic PEMWE conditions. The authors propose that Ag lowers oxophilicity, while Ir enhances oxidation resistance through electronic hybridization. Notably, the nanocages exhibited 1500 hours of durability at 1 A/cm^2 in a full PEMWE device. If fully validated, this result would represent a meaningful advance toward durable, non-oxide OER catalysts. Overall, the work is timely and potentially impactful. However, to meet the standards of Nature Communications, several aspects of the mechanistic interpretation and experimental evidence require clarification.

Response: We thank the reviewer for their positive assessment of our metastable AgRuIr alloy nanocages and for recognizing the 1500-hour durability achieved at 1 A cm^{-2}. We appreciate the

recognition of our work as a “meaningful advance toward durable, non-oxide OER catalysts” and for the constructive feedback regarding our mechanistic interpretations. In accordance with these suggestions, we have significantly strengthened the manuscript by providing additional supporting experimental data. Below, we provide a point-by-point response to the specific technical concerns.

1. The authors attribute the improved oxidation resistance to Ir “elevating the reduction potentials” of Ru and Ag within the alloy through electronic hybridization. This is an important claim, yet it is not currently substantiated by direct measurements or calculated electronic descriptors. The authors should provide quantitative evidence demonstrating how Ir modifies redox energetics in the alloy environment. Without such analysis, the mechanistic conclusion remains speculative.

Response: We thank the reviewer for this insightful comment. To substantiate our claim that Ir elevates the reduction potentials of the alloy components through electronic hybridization, we performed potential-dependent in situ Raman spectroscopy.

For clarity and to avoid the spectral complexity associated with multi-component systems, we simplified our validation by comparing a binary RuIr alloy to pure Ru. This approach allows us to isolate and directly observe the stabilizing effect of Ir on a Ru-based lattice, which serves as a representative model for the broader AgRuIr system. The results, now provided in **Fig. S43** of the revised Supporting Information, reveal a significant difference in the onset of surface oxidation:

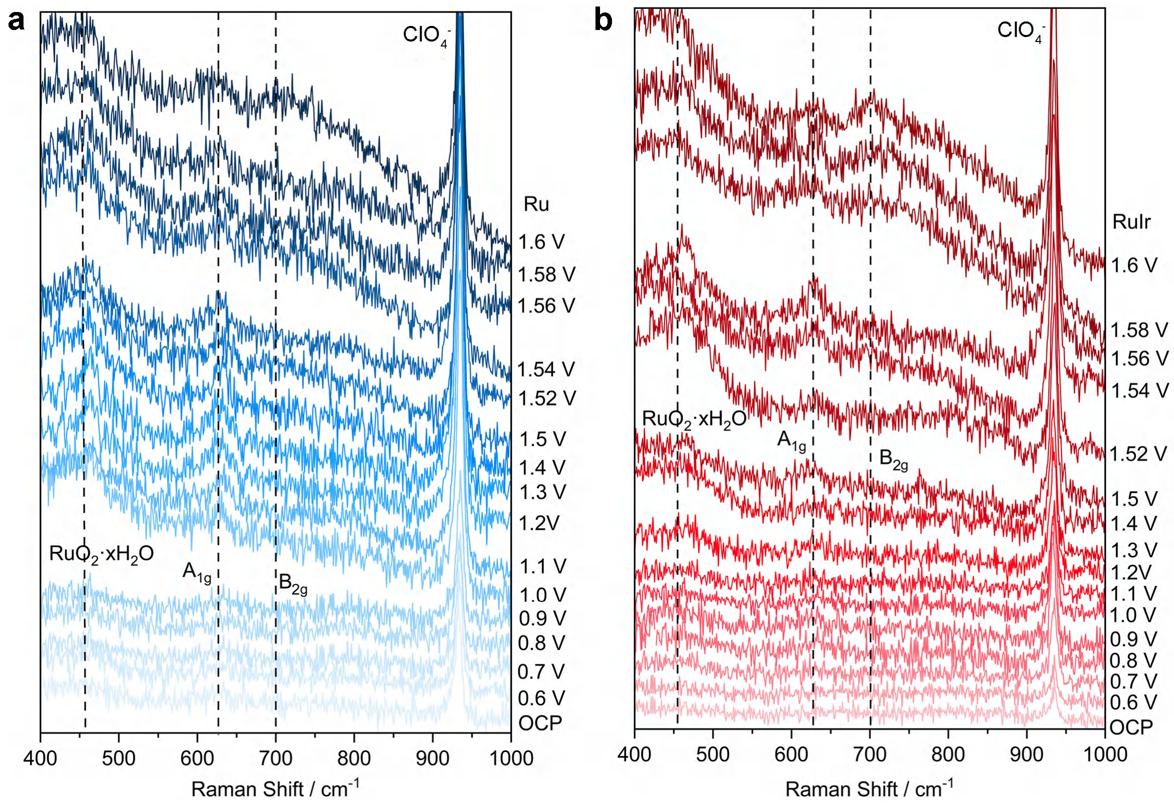

Fig. S43. Validating the role of Ir in elevating the reduction potentials of metals in the alloy system. (a) Potential-dependent in situ Raman spectra of Ru/C in O2-saturated 0.1 M HclO4 from OCP to 1.6 V. (b) Corresponding in situ Raman spectra for RuIr/C under identical conditions. The dashed

lines indicate the positions of characteristic vibrational modes for rutile-type oxides amorphous hydrated RuO_2 ($RuO_2 \cdot H_2O$). The delayed emergence of these features in RuIr compared to pure Ru demonstrates that Ir elevates the oxidation potential of the alloy, providing direct evidence for enhanced oxidation resistance.

For the Ru/C catalyst, characteristic peaks corresponding to rutile-type oxide species (specifically the A_{1g} and B_{2g} modes of RuO_2) and amorphous hydrated RuO_2 ($RuO_2 \cdot H_2O$) begin to emerge at a significantly lower potential (~1.0 V vs. RHE). In contrast, the RuIr/C catalyst remains relatively featureless until higher potentials are reached (~1.5 V vs. RHE), effectively delaying the formation of the oxide species. This shift to higher potentials confirms that the presence of Ir in the alloy environment elevates the reduction/oxidation potentials of the surface, making the metallic state more thermodynamically stable under OER conditions.

Corresponding discussion have been supplemented to the revised manuscript:

Page 16: "We propose that Ir elevates the reduction potentials of the alloy components through electronic hybridization (standard reduction potentials for pure Ru, Ag, and Ir are 0.455, 0.800, and 1.156 V, respectively), thus synergistically enhancing the oxidation resistance of the alloy nanocages. To validate this mechanism, we utilized in situ Raman spectroscopy to monitor the potential-dependent surface oxidation of a binary RuIr alloy system compared to pure Ru (**Fig. S43**). For Ru/C, characteristic vibrational modes corresponding to rutile-type oxide species and amorphous $RuO_2 \cdot H_2O$ began to emerge at potentials as low as ~1.0 V vs. RHE. In contrast, the RuIr/C catalyst remained relatively featureless until significantly higher potentials are reached (~1.5 V vs. RHE). This clear delay in the formation of surface oxides provides direct experimental evidence that Ir elevates the reduction potentials of the alloy surface, maintaining superior thermodynamic stability under demanding OER conditions."

2. XPS and Raman spectroscopy suggest an absence of bulk oxide formation. However, both techniques probe only limited depths and may not completely eliminate the possibility of subsurface oxidation, especially considering the ~3.8 nm shell thickness. The authors should explicitly discuss these limitations and, if possible, reference operando XAS or other, more bulk-sensitive evidence to reinforce their claim that the alloy remains metallic under elevated potentials.

Response: We thank the reviewer for the insightful comments. We acknowledge that while XPS is an excellent surface-sensitive tool, its limited probing depth makes it primarily sensitive to the catalyst-electrolyte interface. To address the possibility of subsurface or bulk oxidation within the 3.8 nm walls, we have integrated a suite of bulk-sensitive characterizations:

(1) Raman spectroscopy: As a relatively bulk-sensitive technique for these nanocrystals, Raman spectroscopy (**Fig. S28**) shows no characteristic peaks for rutile-type or amorphous metal oxides even after 100 h of operation at 200 mA cm^{-2}.

(2) HAADF-STEM imaging: We have provided high-resolution STEM images (**Fig. 4**k and **Fig. S30**) that clearly resolve continuous metallic lattice fringes throughout the nanocage walls after 200 h of testing at 200 mA cm^{-2}, confirming that the structural skeleton remains an alloy.

(3) Ru FT-EXAFS: Following the reviewer's suggestion to include X-ray absorption spectroscopy, we have added Ru FT-EXAFS data (**Fig. S32**) of the nanocages after catalysis. The Fourier-transformed spectra of the post-OER samples are nearly identical to the pristine metallic nanocages, providing definitive bulk-sensitive evidence that the Ru coordination environment remains metallic.

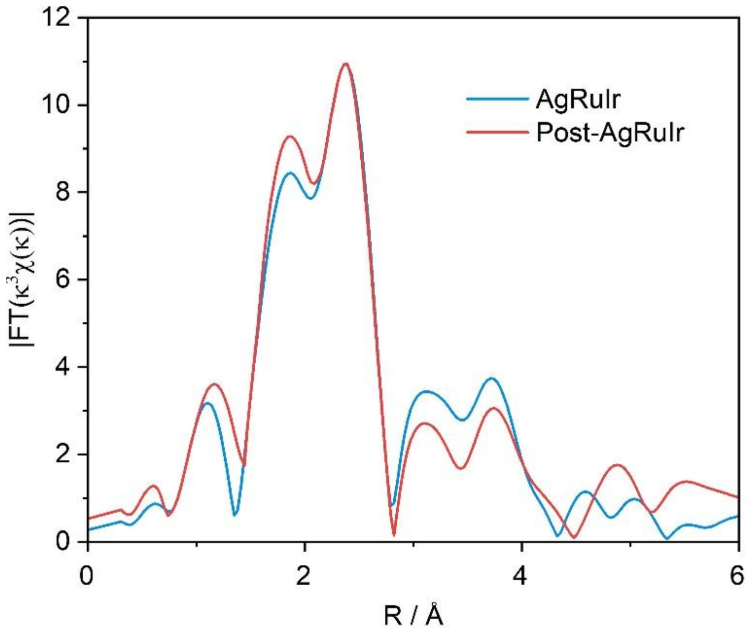

Fig. S32. Ru FT-EXAFS of the $Ag_{0.19}Ru_1Ir_{0.48}$ alloy nanocages before and after OER catalysis. As a bulk-sensitive technique, X-ray absorption spectroscopy confirms the retention of the bulk metallic state in the $Ag_{0.19}Ru_1Ir_{0.48}$ alloy nanocages during the OER process.

Following the reviewer's suggestion, we have discussed the limitations of XPS analysis and supplemented more bulk-sensitive evidence (including XAS) to reinforce our discussion on the retention of the metallic alloy state under elevated potentials:

Page 15: "While XPS as a surface-sensitive technique detects the thin surface layer, bulk-sensitive characterizations confirm the persistence of the metallic framework. The Raman spectrum of the $Ag_{0.19}Ru_1Ir_{0.48}$ catalyst after chronopotentiometric testing at 200 mA cm^{-2} for 100 h exhibited no oxide-related peaks (i.e., surface hydrated or rutile RuO_2), confirming high oxidation resistance at the high OER potentials (**Fig. S28**). Post-catalytic HAADF-STEM and EDS mapping further confirmed the preservation the metallic crystalline structure and uniform elemental distribution (**Fig. 4**k, l, **Fig. S29–S31**). Furthermore, post-catalytic Ru FT-EXAFS provided definitive bulk-sensitive evidence for the retention of the metallic alloy state during the OER process (**Fig. S32**)."

3. The explanation for the enhanced performance focuses on the low oxophilicity of Ag. However, introducing Ag also imposes lattice strain and alters local coordination, both of which can significantly influence the d-band structure and active-site energetics. Therefore, the authors should discuss whether strain or structural disorder contributes comparably to the observed improvements.

Response: We thank the reviewer for this insightful comment. We agree that the introduction of Ag into the RuIr lattice induces lattice strain and structural disorder, which can significantly modulate the d-band structure. In our system, the larger atomic radius of Ag (144 pm) compared to Ru (134 pm) and Ir (136 pm) is expected to introduce tensile strain.

To decouple the effects of strain from oxophilicity, we performed comparative studies using CuRuIr counterpart. Given that Cu (128 pm) is smaller than Ru and Ir, this system is expected to introduce compressive strain. Interestingly, the Cu-incorporated catalysts also exhibited enhanced OER activity compared to pure RuIr (**Fig. S41**). This observation—that both tensile and compressive strains lead to improved performance—suggests that the specific nature of the lattice strain is not the primary driver of the OER activity in this system.

However, we acknowledge that structural disorder (or lattice distortion) inherent in these metastable alloy nanocrystals likely contributes to the enhanced OER activities. Nevertheless, while both alloy systems (AgRuIr and CuRuIr) may introduce lattice distortion, only AgRuIr exhibited exceptional OER durability (**Fig. S42**). This points to the conclusion that the modulation of surface oxophilicity remains the primary mechanism for the enhanced stability observed in our catalyst. We have added a discussion of these points to the revised manuscript to provide a more nuanced mechanistic interpretation.

Page 17: "Beyond oxophilicity, we considered the influence of geometric factors like lattice strain and structural disorder. While Ag introduces tensile strain, our comparative studies with CuRuIr, which impose compressive strain due to the smaller radii of Cu, also showed enhanced OER activity compared with RuIr (**Fig. S41**). This suggests that the specific nature of lattice strain is not the primary driver of performance. Furthermore, while the structural disorder inherent in these metastable alloys likely provides a beneficial distribution of active sites, only the AgRuIr system demonstrated exceptional durability (**Fig. S42**). These results indicate that while lattice distortion may contribute to the enhanced activity, the modulation of surface oxophilicity remains the decisive mechanism for long-term stability."

4. The reported breakdown of the conventional *OH/*OOH scaling is intriguing. However, more detail is needed to determine if it is robust or model-dependent. The authors should clarify how sensitive the results are to surface *O coverage and determine whether the quasi-random alloy structure accurately represents realistic surface heterogeneity. Providing such clarification would improve confidence in the generality of the mechanistic conclusions.

Response: We thank the reviewer for the insightful comments regarding the robustness of our DFT modeling. We have addressed the concerns about model dependency and surface heterogeneity as follows:

(1) An $Ag_8Ru_{38}Ir_{18}$ alloy structure was generated to simulate the $Ag_{0.19}Ru_1Ir_{0.48}$ alloy composition. The model was constructed using the Alloy-Theoretic Automated Toolkit (ATAT) code. The core of ATAT is the generation of Special Quasi-random Structures, which are designed to mimic the atomic correlation functions of a true random solid solution within a finite supercell. This approach effectively captures the surface heterogeneity and diverse local coordination environments of the experimental metastable alloy.

(2) Following the theoretical framework established by Nørskov regarding oxygen coverage (θ) on metal surfaces (ref. 40), we investigated the influence of θ on OER energetics. Our results indicate that on a pristine surface, the oxidation of *O to *OOH is the potential-determining step (PDS). Our DFT calculations show that increasing the oxygen coverage from θ = 0 to 3/4 causes the reaction energy of the PDS to decrease significantly from 1.30 eV to 0.87 eV at U = 1.23 V. This coverage-dependent reduction in the energy barrier suggests that the catalyst surface readily evolves toward a high-coverage state that facilitates spontaneous OER. By explicitly accounting for these coverage effects, our model more accurately reflects the "oxygenated metallic surface" identified in our experiments, providing confidence in the mechanistic conclusions.

We have included the detailed data in the Supporting Information (**Fig. S46**) and revised the corresponding discussion in the revised manuscript.

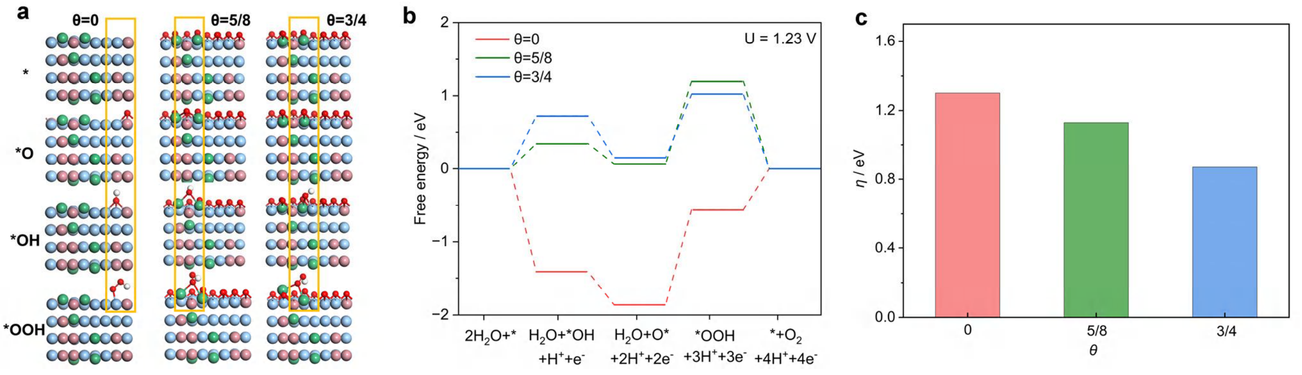

Fig. S46. Effect of surface oxygen coverage (θ) on AgRuIr alloy surface on OER kinetics. (a) Adsorption configurations of oxygenated OER intermediates on alloy surfaces at different surface oxygen coverages (θ = 0, 5/8, and 3/4). (b) Gibbs free energy profiles of the OER on AgRuIr with varying surface oxygen coverages. (c) Energy increase of the potential-determining step as a function of surface oxygen coverage.

Page 17: "Additionally, surface oxygen coverage (θ_{*O}) was also found to significantly influence OER kinetics, aligning with the theoretical framework established by Nørskov and co-workers[40]. Given the high energy increase required for *O → *OOH, *O species tend to accumulate on the metal surface,

reaching high coverage before the OER proceeds. Our DFT calculations demonstrate that with increasing θ_{*O}, the energy increase for the PDS (*O → *OOH) markedly decreases (**Fig. S46**). Specifically, at $\theta_{*O} = 3/4$, the energy increase for this step reaches 2.10 eV at $U = 0$ and 0.87 eV at $U = 1.23$ V. This reduction upon oxygen coverage stems from the differential modulation of adsorption energies: increasing surface oxygen coverage destabilizes *O more significantly than *OOH. This relative destabilization reduces the energy increase required for *OOH formation, thereby promoting the overall OER kinetics. These results demonstrate that AgRuIr, characterized by a spontaneously formed surface *O layer at operating potentials, is highly efficient for OER catalysis."

Again, we would like to thank all the reviewers for their insightful comments and constructive suggestions, which have been invaluable in improving the quality of this work. We also thank the editor for the time and effort spent in handling our manuscript.

Chuanbo Gao
On behalf of all authors

Response to Reviewers

Reviewer #3: I would like to thank the authors for their detailed reply and for addressing all of the improvements suggested, as well as for including new characterizations that further strengthen the conclusions of the article. The manuscript is acceptable for publication, however, I would like to highlight two points:

Response: We are sincerely grateful to the Reviewer for the thoughtful evaluation and the positive assessment of our revised manuscript. We appreciate the recognition that our additional characterizations have further strengthened the conclusions of this study. The reviewer's insights have been invaluable in refining our mechanistic understanding and improving the overall quality of this work. We have carefully addressed the two specific points highlighted below and have updated the manuscript accordingly.

1) I would like to emphasize that, indeed, during the first hours of operation, the activity of the AgIrRu catalyst is associated with the metallic character of Ru and Ir. However, after 100 hours, the XPS data show that all Iridium has become oxidized, which is reasonable considering that the nanocage shell thickness is 3.8 nm, approximately the depth probed by XPS. What is important, and should be explicitly stated in the manuscript, is that the metallic state of Ru is preserved even after 1000 hours of operation, as confirmed by several techniques (XPS and FT EXAFS). The interaction between this metallic Ru and IrO2 may be responsible for the catalyst's activity and long term stability.

Response: We fully agree with the reviewer's perspective regarding the evolution of the surface state and the resulting synergy between the components. While both Ru and Ir are initially metallic, our XPS data indeed suggest that Ir on the surface (and potentially throughout the 3.8 nm shell) evolves into an oxidized state (IrO_2) after extended catalysis. However, as the reviewer emphasized, the most striking finding is the persistence of the metallic Ru^0 state even after 1000 h of operation, as confirmed by both surface-sensitive (e.g., XPS) and bulk-sensitive (e.g., FT-EXAFS) techniques. We have followed the reviewer's suggestion and explicitly stated in the revised manuscript that the exceptional long-term stability and activity likely arise from the unique interaction between the preserved metallic Ru and the in situ formed IrO_2 layer. This synergistic effect helps maintain high OER activity while preventing the excessive oxidation and dissolution of Ru.

The following revision has been made to the manuscript (**Pages 15-16**):

"Notably, the metallic Ru^0 state was preserved even after 1000 h of chronopotentiometric testing at 200 mA cm^{-2} (Fig. S28) ……Crucially, the remarkable persistence of metallic Ru alongside the in situ formation of IrO_2 after extended catalysis suggests a unique synergistic mechanism. The electronic interaction at the interface of the stable metallic Ru and the protective IrO_2 layer likely provides the necessary environment to sustain high OER activity while effectively shielding the Ru from excessive oxidation and subsequent dissolution."

2) Regarding the PEMWE experiments suggested in my Question 4, I appreciate that the authors

followed all of our recommendations. Nevertheless, I would like to request further clarification on the following point to be addressed by the authors: In the dynamic start-stop cycling experiments shown in Fig. S49, it is surprising how stable the voltage remains throughout the different cycles, with no apparent changes even during start cycle after OCP periods where a potential "jump" is usually observed, followed by a gradual decrease until stabilization. Could the authors provide a zoom-in of these results and/or the raw data?

Response: We appreciate the reviewer's careful inspection of the start-stop cycling data. We would like to clarify the unusual stability of the potential profile and provide the requested raw data.

First, we sincerely apologize for a technical error in the data representation of Fig. S49 in the previous version. During the preparation of the figure, an unnecessary potential offset was subtracted from the shutdown potential values. We have now corrected this in the revised Supporting Information by replacing the figure with the original, unadjusted instrumental data directly from the electrochemical workstation.

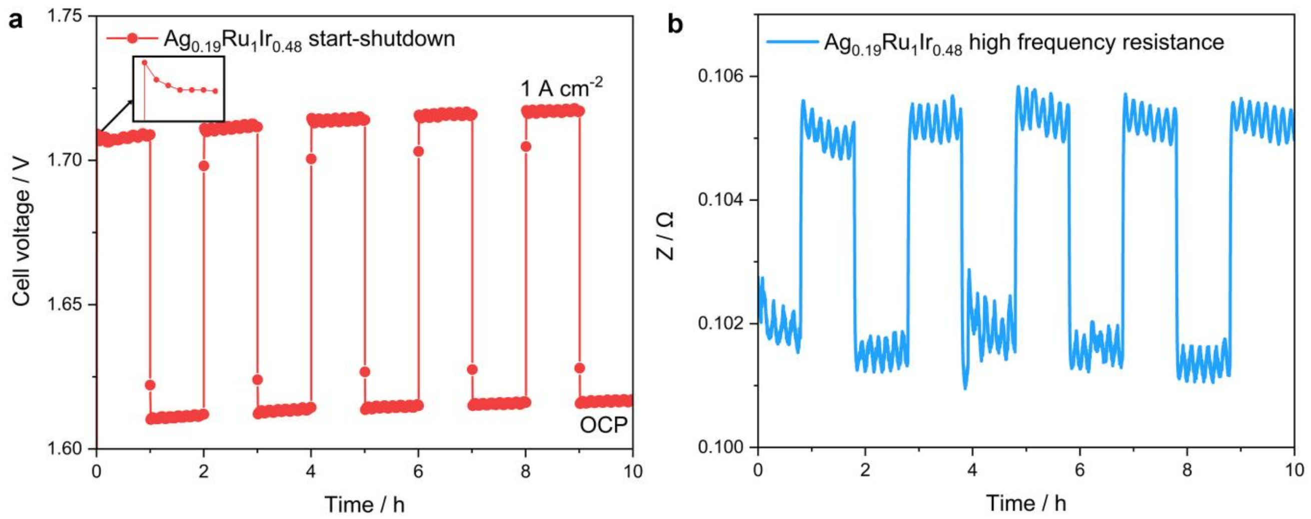

Fig. S49. Dynamic start-stop cycling performance of $Ag_{0.19}Ru_1Ir_{0.48}$ alloy nanocages in a PEMWE single cell. (a) Cell voltage profile during intermittent start-stop cycles, consisting of alternating periods of 1 h operation at 1 A cm^{-2} and 1 h at open-circuit potential (OCP). Inset: A zoom-in view of the cell-voltage profile during the transition from OCP to the start of the next cycle, demonstrating the rapid stabilization of the potential without significant voltage transients or "jumps." This behavior suggests that as a metallic catalyst, the surface oxygenated species of $Ag_{0.19}Ru_1Ir_{0.48}$ can reach electrochemical equilibrium almost instantaneously at the onset of catalysis. This effectively avoids the energy-consuming reduction/re-oxidation cycles typical of conventional oxide catalysts, where a potential jump is usually observed during the re-activation of the surface. (b) Real-time monitoring of high-frequency resistance (HFR) during the cycling test, confirming the robust structural and electrical integrity of the MEA under dynamic operating conditions.

Regarding the absence of the typical potential "jump" and the rapid stabilization, we believe this is an intrinsic characteristic of our metallic alloy catalyst. In many conventional oxide-based systems, a significant potential jump occurs primarily because the catalyst surface undergoes partial reduction or

structural relaxation during the OCP period, necessitating a re-oxidation or "activation" period upon restarting. In contrast, our $Ag_{0.19}Ru_1Ir_{0.48}$ catalyst maintains a robust metallic framework. Because the surface oxygenated species can reach electrochemical equilibrium almost instantaneously at the onset of catalysis, the system avoids the energy-consuming reduction/re-oxidation cycles typical of conventional oxide catalysts. This rapid equilibrium ensures a near-seamless transition from OCP back to high-current operation, effectively eliminating the potential transients observed in other systems.

As requested, we have included a zoom-in of the cycles in Fig. S49 and provided the raw data for reference (uploaded separately). The corresponding discussion has been added to the caption of Fig. S49.

Again, we would like to thank all reviewers for their insightful comments and constructive suggestions.

Chuanbo Gao
On behalf of all authors

Response to Reviewers

Reviewer #3: The authors have addressed the issues raised by the reviewers. The manuscript is ready for publication at the present state.

Response: We thank the reviewer for the positive evaluation and support for publication.

Reviewer #4: I co-reviewed this manuscript with one of the reviewers who provided the listed reports. This is part of the Nature Communications initiative to facilitate training in peer review and to provide appropriate recognition for Early Career Researchers who co-review manuscripts.

Response: We thank the reviewer for their contribution to the peer review process and for the positive evaluation of our manuscript.

We express our sincere appreciation to all reviewers involved in the peer-review process for their insightful comments, which have greatly improved the quality of this work.

Chuanbo Gao
On behalf of all authors
